# Transcriptome and metabolome reveal distinct carbon allocation patterns during internode sugar accumulation in different sorghum genotypes

**DOI:** 10.1111/pbi.12991

**Published:** 2018-09-15

**Authors:** Yin Li, Wenqin Wang, Yaping Feng, Min Tu, Peter E. Wittich, Nicholas J. Bate, Joachim Messing

**Affiliations:** ^1^ Waksman Institute of Microbiology Rutgers, The State University of New Jersey Piscataway NJ USA; ^2^ Syngenta Crop Protection, LLC Greensboro North Carolina USA; ^3^ Present address: School of Agriculture and Biology Shanghai Jiaotong University Shanghai China

**Keywords:** RNA‐seq, metabolomics, gene expression, sugar accumulation, trehalose‐6‐phosphate signalling, introgression, sorghum, internode

## Abstract

Sweet sorghum accumulates large amounts of soluble sugar in its stem. However, a system‐based understanding of this carbohydrate allocation process is lacking. Here, we compared the dynamic transcriptome and metabolome between the conversion line R9188 and its two parents, sweet sorghum RIO and grain sorghum BTx406 that have contrasting sugar‐accumulating phenotypes. We identified two features of sucrose metabolism, stable concentrations of sugar phosphates in RIO and opposite trend of trehalose‐6‐phosphate (T6P) between RIO vs R9188/BTx406. Integration of transcriptome and metabolome revealed R9188 is partially active in starch metabolism together with medium sucrose level, whereas sweet sorghum had the highest sucrose concentration and remained highly active in sucrose, starch, and cell wall metabolism post‐anthesis. Similar expression pattern of genes involved in sucrose degradation decreased the pool of sugar phosphates for precursors of starch and cell wall synthesis in R9188 and BTx406. Differential T6P signal between RIO vs R9188/BTx406 is associated with introgression of T6P regulators from BTx406 into R9188, including C‐group bZIP and trehalose 6‐phosphate phosphatase (TPP). The inverted T6P signalling in R9188 appears to down‐regulate sucrose and starch metabolism partly through transcriptome reprogramming, whereas introgressed metabolic genes could be related to reduced cell wall metabolism. Our results show that coordinated primary metabolic pathways lead to high sucrose demand and accumulation in sweet sorghum, providing us with targets for genetic improvements of carbohydrate allocation in bioenergy crops.

## Introduction

Sorghum (*Sorghum bicolor* L. Moench) is a C4 crop plant widely used for food, fodder, fibre and fuel. Its use for fuel has emerged because of several advantages, such as high biomass yield, abiotic stress tolerance, high water and nitrogen use efficiency, and rich genomic resources (Calvino and Messing, 2012; McCormick *et al*., 2018; Mullet *et al*., 2014; Paterson *et al*., 2009). The sweet sorghum ideotype accumulates increased soluble sugars in the stem with a high biomass yield, making it not only a desirable bioenergy crop, but also a genetic model for related species like sugarcane (Wang *et al*., 2013).

Therefore, stem sugar accumulation in sorghum has gained attention. Carbohydrates, primarily sucrose, are produced during photosynthesis and transported to other parts of the plant for energy consumption and storage. In the source‐sink context, the photosynthetically active leaves are considered source organs, whereas both the stem and developing seeds of sweet sorghum that utilize and store energy are considered sink organs, and the balance between source and sink is tightly controlled (Griffiths *et al*., 2016; Yu *et al*., 2015b). Accumulation of stem sucrose is associated with cessation of internode growth, which coincides with anthesis for sorghum varieties not very sensitive to photoperiod (Gutjahr *et al*., 2013a,b; McKinley *et al*., 2016; Tovignan *et al*., 2016). Sucrose is unloaded apoplastically from stem phloem, taken up by stem storage parenchyma cells, and stored in vacuoles (Bihmidine *et al*., 2015; Tarpley and Vietor, 2007). Quantitative trait loci (QTL) mapping and genome‐wide association studies (GWAS) identified several QTLs contributing to sugar‐related traits (Brenton *et al*., 2016; Burks *et al*., 2015; Guan *et al*., 2011; Murray *et al*., 2008; Natoli *et al*., 2002; Ritter *et al*., 2008; Shiringani *et al*., 2010). These studies showed that genetic and genotype X environment effects were significant for sugar–related traits, whereas physiological and phenological studies revealed that drought stress and sowing date could influence stalk sugar yield, possibly by directly affecting internode growth, soluble sugar/lignin ratio, and by indirectly changing leaf area and photosynthesis (Perrier *et al*., 2017; Tovignan *et al*., 2016; Trouche *et al*., 2014). A correlation between plant height (PHT) and stalk sugar yield was repeatedly observed even though the correlation varied between studies. Several studies detected weak correlations of PHT with sucrose concentration (Brix) but strong correlations with juice yield/weight (Guan *et al*., 2011; Murray *et al*., 2008; Ritter *et al*., 2008). Indeed, a GWAS study showed that sweet sorghum had significantly higher juice volume, stem juiciness and sugar yield compared with non‐sweet landraces, but there was no statistical difference in Brix between the two groups (Burks *et al*., 2015). Experiments using sorghum lines with distinct PHT (caused by the *dwarf3* locus) or maturity (photoperiod–sensitive vs converted) further support that at least some dwarfism or maturity loci might not affect stem sugar concentration (Shukla *et al*., 2017). Genetics breaks the trait of stalk sugar yield into two quantitative traits, sugar concentration and juice yield, with the latter affected by both juiciness and biomass. Additionally, based on field experiments stalk weight and moisture independently affect juice yield (Carvalho and Rooney, 2017).

Efforts have also been made to understand stem sugar accumulation at phenotypic diversity, physiological and molecular levels with various approaches including targeted gene expression analysis (Li *et al*., 2018; Milne *et al*., 2013; Mizuno *et al*., 2016; Qazi *et al*., 2012), whole genome resequencing (Zheng *et al*., 2011), microarray (Calvino *et al*., 2008; Jiang *et al*., 2013), small RNA sequencing (Calvino *et al*., 2011; Yu *et al*., 2015a). Previous studies showed that neither sucrose metabolizing enzymes nor expression difference of *Sucrose Transporters* (*SUTs*) between sweet and grain sorghum were correlated with sugar accumulation (Bihmidine *et al*., 2015; Hoffmann‐Thoma *et al*., 1999; Milne *et al*., 2013). *Tonoplast Sugar Transporters* (TST) might play roles due to their differential expression between sweet and grain sorghum stems (Bihmidine *et al*., 2016). Time‐course RNA‐seq revealed correlation of sucrose accumulation with down‐regulation of vacuolar invertase (SbVIN1) and sucrose synthase (SbSUS4; McKinley *et al*., 2016). Another RNA‐seq study proposed *Sugars Will Eventually be Exported Transporters* (SWEET) as candidates for sucrose efflux from leaf and stem phloem (Mizuno *et al*., 2016). Transcriptome analysis on its own, however, has limitations in predicting central metabolism (Fernie and Stitt, 2012; Schwender *et al*., 2014). Therefore, integration of the transcriptomic and metabolomic profiles over a time course will increase our understanding of sugar accumulation in sorghum.

In contrast with sweet sorghum, grain sorghum genotypes in the US are dwarf and flower early, but R9188 is a sweet sorghum‐converted line in which the genetic loci controlling dwarfism and early flowering were introgressed from grain sorghum BTx406 (Ritter *et al*., 2008). To distinguish between key factors controlling sugar accumulation and other molecular changes during reproductive stages, we took advantage of the relationships between R9188 and their parents RIO and BTx406, and characterized their distinct sugar phenotypes under field conditions. Here, we report the first integration of dynamic transcriptome and metabolome from sucrose‐accumulating internodes of the three genotypes with the following objectives: (i) to document gene expression patterns and changes of metabolites over the time course of sugar accumulation; (ii) to compare differences between the three genotypes at the metabolome and transcriptome levels; (iii) to obtain a system understanding of metabolic and molecular mechanisms underlying the sugar‐accumulation process; (iv) to identify candidate genes involved in this process for breeding improvement and genetic engineering.

## Results

### Spatiotemporal dynamics of sugar accumulation in sorghum stems

**Figure 1:**
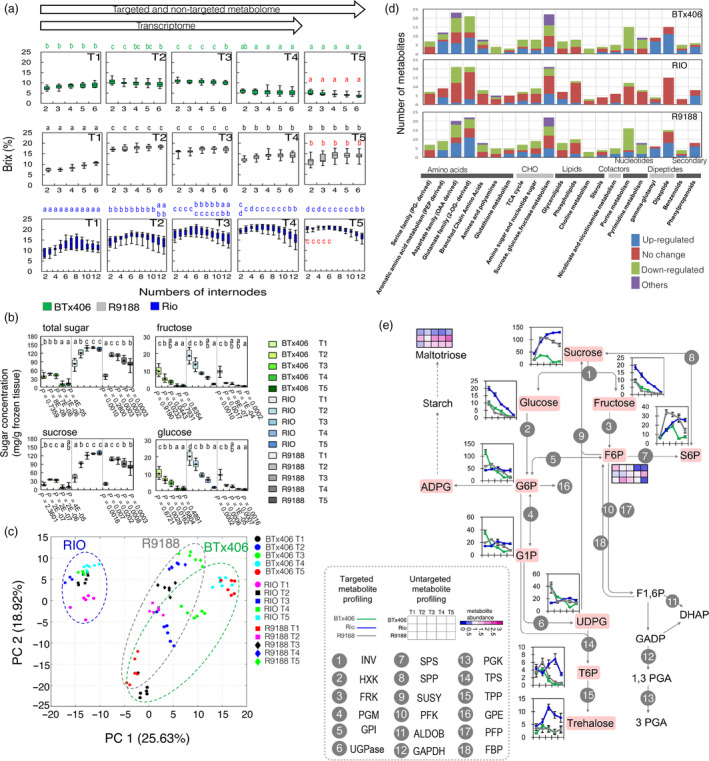
Characterization of metabolism in sorghum upper internodes: (a) Changes in internode Brix over time (*n* = 9). Differences in the same internodes between stages are displayed by letter within genotypes, while red letter indicates significant difference between genotypes at T5. (b) Dynamics of sucrose, glucose, fructose, and total sugar (sum of the three sugars) in upper internodes (*n* = 6); Differences of upper‐internode sugar concentrations are indicated by letter, while *P* values for the statistical comparisons of BTx406‐R9188 and RIO‐R9188 are shown below the BTx406 and R9188 figures, respectively (Welch *t* test). (c) PCA analysis of metabolome samples; (d) Comparison of differential metabolites between genotypes. (e) Metabolite dynamics in the sucrose metabolism (*n* = 6; error bar = SEM). All statistical differences displayed by letter were determined by ANOVA and Tukey test (*P *<* *0.05).

We assessed the spatiotemporal progression of sugar accumulation in sorghum stems by measuring the internode total solute levels (Brix) from RIO, BTx406 and R9188 at five developmental time points, ranging from flag leaf stage to 30 days after flowering (designed as T1 to T5; Experimental Procedures; Figures 1a, S1 and S2; Table S1). BTx406 and R9188 reached their highest Brix during flowering (T2), but then their Brix values decreased, whereas the Brix values of RIO kept increasing until T5 with the highest Brix values detected in the upper internodes, internodes No. 2 to No. 6. In contrast to RIO, the Brix values from BTx406 and R9188 were similar between internodes.

The concentration of several sugars and sugar phosphates from the upper internodes were quantified by using targeted metabolic profiling (Data S1 and Table S2). The total sugar concentration was highly correlated with the Brix values in all three genotypes, and sucrose accounted for most soluble sugars (Figure 1b). Sucrose was increased at T2 followed by a decrease after T3 in R9188 and significantly different over time compared to RIO, suggesting distinct sucrose accumulation patterns between genotypes. Glucose and fructose concentrations in upper internodes rapidly decreased over time in all three genotypes regardless of sucrose patterns.

### Metabolic features of sucrose metabolism in sweet sorghum stem

To systematically profile metabolic changes in stems, we performed an untargeted metabolome analysis and detected a total of 507 biochemicals, of which 300 were known metabolites identified in all three genotypes (Data S2 and Table S3). Metabolites covered eight major categories, including amino acids, carbohydrates, cofactors, lipids, nucleotides, peptides, secondary metabolites and hormones (Figure S3). The quality of non‐targeted metabolome data was high, as evidenced by the high correlation of eight metabolites detected by both targeted and non‐targeted methods (Figure S4). Principal component analysis (PCA) showed that ~44% of metabolic variances between samples were explained by principal components 1 and 2 (Figure 1c), corresponding to genotype and time point. RIO samples formed a distinct separation from samples of BTx406 and R9188 and metabolome data of R9188 and BTx406 were relatively close to each other at T1 and T2, and gradually separated from T3 to T5.

We plotted significant differential metabolites in each genotype based on their categories to obtain an overview of metabolic changes (Figure 1d). Consistent with the PCA results, metabolic changes in amino acids, carbohydrates, lipids and peptides were similar between R9188 and BTx406, but different from RIO. Particularly, in R9188 metabolite changes in several pathways resembled those in BTx406, such as aromatic amino acid, aspartate, glutamate, branched‐chain amino acid, TCA cycle and sucrose metabolic pathways, whereas in RIO many metabolites were not significantly changed, indicating a relatively stable metabolic status over time. Sixty‐seven metabolites were subsequently plotted onto a representative map of central metabolism (Figure S5). Correlation of amino acids revealed that most of them showed coordinated changes in R9188 and BTx406 but not in RIO, especially between the aspartate, glutamate family, and branched‐chain amino acids (BCAA) (Figure S6).

For sucrose metabolism, RIO differed from BTx406 and R9188 in two metabolic features (Figure 1e). First, sweet sorghum had stable concentrations of several sugar phosphates, including glucose 6‐phosphate (G6P), fructose 6‐phosphate (F6P), glucose 1‐phosphate (G1P) and uridine diphosphate glucose (UDPG), compared with gradual decrease of these metabolites in R9188/BTx406. Second, the trehalose pathway showed the opposite trend between RIO and R9188/BTx406. T6P was increased at anthesis followed by a drastic decrease post‐anthesis in R9188/BTx406, whereas it showed a slight decrease at anthesis and a gradual increase afterwards in RIO.

### Transcriptome analyses

**Figure 2:**
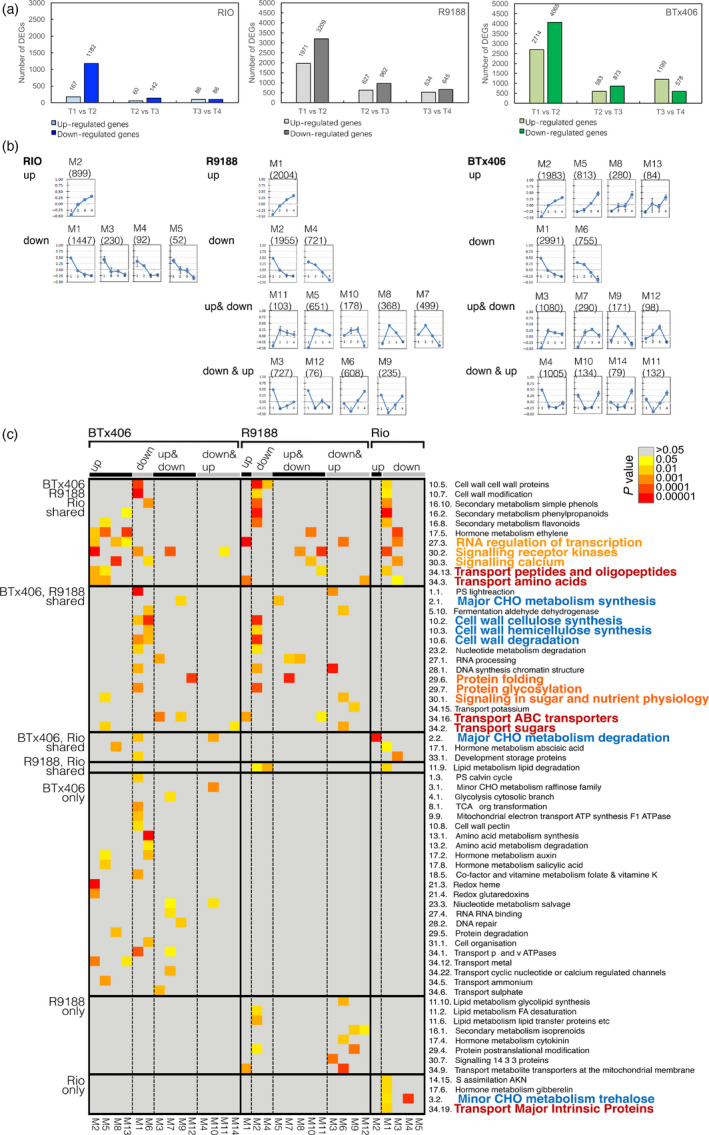
Overview of DEGs (a). Expression patterns of eigengenes from co‐expression networks for RIO, BTx406 and R9188 (b). Enriched MapMan functional categories in the co‐expression modules (top 5 enriched categories, *P* value <0.05 and *q* value <0.2). Columns are ordered first by genotype and then by module, with rows ordered by whether the categories are shared in different genotypes. MapMan categories belong to central metabolism; transport and regulation are colored in blue, red and orange, respectively.

Time‐course RNA‐seq were performed using the same stem samples for metabolome analysis from flag leaf stage to 15 days after flowering (T1 to T4; Tables S4, S5). 18 275, 19 727 and 19 102 genes were expressed in RIO, BTx406 and R9188, respectively (Table S6, Appendix S1). In all three genotypes, the number of DEGs marked decreased from T1‐T2 comparison to the other comparisons (T2‐T3 and T3‐T4), suggesting drastic transcriptome changes in stem tissue from flag leaf to flowering stages (Figure 2a). Additionally, the number of genes up‐ or down‐regulated in RIO was much smaller than those in R9188 and BTx406, indicating that the transcriptome of R9188 and BTx406 changed drastically compared to RIO. This is consistent with metabolic changes in R9188/BTx406 and the relatively stable metabolic status of RIO. The transcriptome dynamics of the three genotypes were analysed with two complementary approaches: (i) We constructed co‐expression networks for each genotype and compared the enriched biological functions of co‐expression modules between genotypes; (ii) We identified DEGs between genotypes at the same time point and dissected differences in RIO vs R9188/BTx406 and RIO/R9188 vs BTx406.

In the first approach, 3250, 8336 and 9996 genes were differentially expressed over time by pair‐wise comparison of RIO, R9188 and BTx406, respectively. The DEGs were then used for weighted gene co‐expression network analysis (WGCNA). The transcriptome networks were constructed for each genotype individually, yielding 5, 14 and 12 co‐expression modules for RIO, BTx406 and R9188, respectively (Figure 2b, Data S3). These modules represent clusters of interconnected genes that share highly similar expression patterns. Then, we calculated and plotted the module eigengenes (ME, the first principal component of a module), which served as representatives of the modules’ gene expression profiles (Figures 2b, S7). We found that these modules were highly robust and reproducible by module preservation analysis (Figure S8). Based on expression trends, DEGs in RIO could only be grouped as up‐ and down‐regulated, whereas the modules in BTx406 and R9188 could be divided into four groups, up‐regulated, down‐regulated, up‐and‐down regulated, and down‐and‐up regulated.

To investigate how genes, biological processes and metabolic pathways change during stem sugar accumulation, we compared the enriched functions of each module between genotypes for MapMan categories, gene ontology biological processes (GOBP), and GO molecular functions (GOMF) (Figures 2c, S9, S10; Data S4). The differentially enriched functions fell into three categories, central metabolism, regulation and transport. MapMan and GO enrichment analyses identified functions associated with major carbohydrate (CHO), cell wall and trehalose metabolic pathways that were overrepresented in modules with distinct expression patterns between genotypes. Enrichment analysis showed that functions associated with transcriptional regulation, signalling transduction and protein modification were either shared in modules with different expression profiles between genotypes or overrepresented in RIO‐ or BTx406/R9188‐specific modules, indicating that regulations at both transcriptional and protein level could be related to reprogramming in stems. Genes were also differentially enriched for sugar, amino acid and oligopeptide transport between genotypes. We next performed enrichment analysis with KEGG and Plant Metabolic Network (PMN) annotations to focus on metabolism (Figure S10; Data S5). These results not only validated the differences in starch, cellulose and trehalose biosynthesis between genotypes, but also showed differential overrepresentation in several metabolic pathways, including glycine, phenylalanine, tyrosine, tryptophan, phenylpropanoid biosynthesis and isoleucine degradation, matching variations in amino acid metabolism between genotypes.

**Figure 3:**
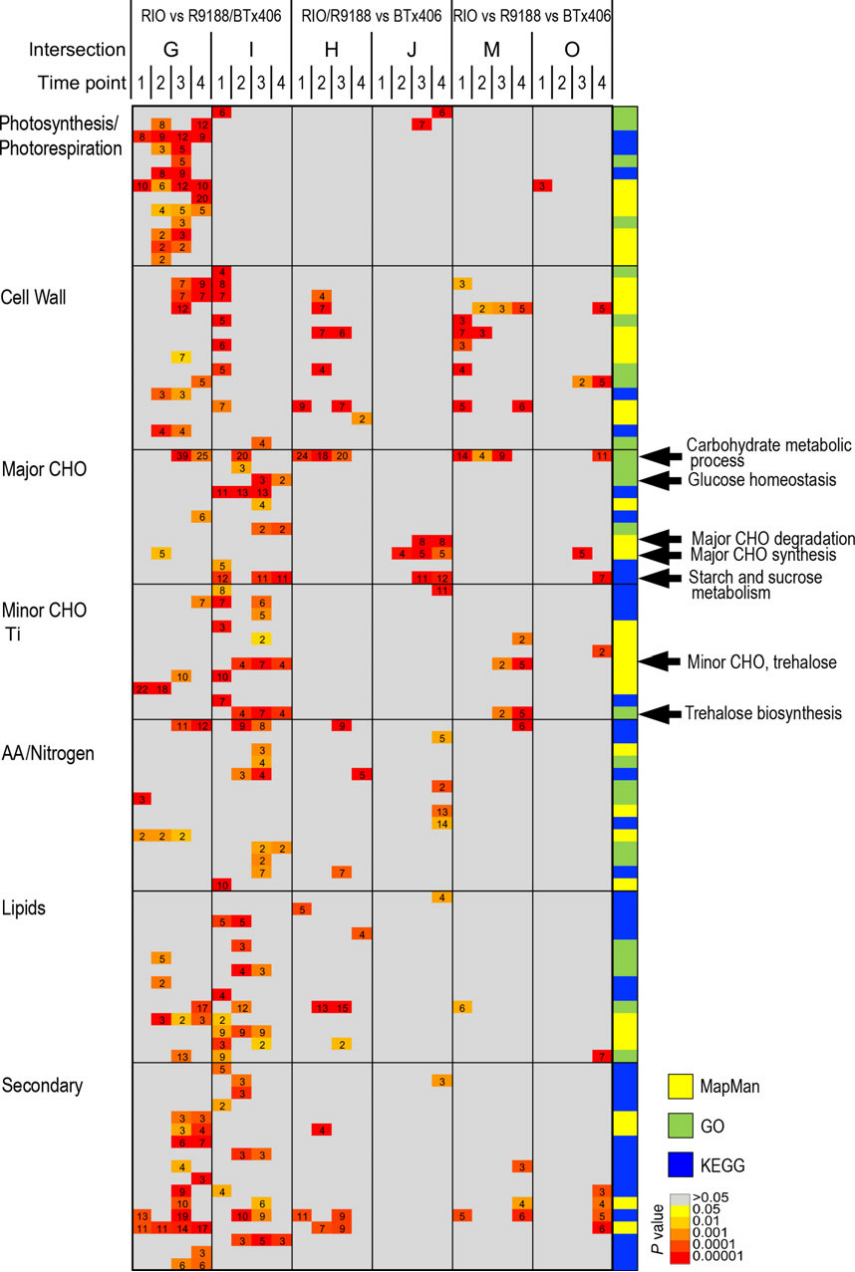
Functional enrichment of genes responsible for genotypic differences (*P* value <0.05 and *q* value <0.2). The intersections were defined as shared sets of DEGs between genotype comparisons, and they represented the differences in RIO vs R9188/BTx406 (intersections G, I), RIO/R9188 vs BTx406 (intersections H, J) and differences between the three genotypes (intersections M, O; Figure S11).

In the second approach, up‐ and down‐regulated genes for the three comparisons (RIO vs R1988, RIO vs BTx406 and R9188 vs BTx406) were intersected, identified the gene sets responsible for differences in RIO vs R9188/BTx406 (intersection G, I) and RIO/R9188 vs BTx406 (intersection H, J; Figure S11). Genes corresponding to the RIO vs R9188/BTx406 difference were enriched by many primary and secondary metabolic pathways ranging from photosynthesis, lipid metabolism to secondary metabolism, whereas genes corresponding to the RIO/R9188 vs BTx406 difference were particularly enriched in carbohydrate metabolic processes including cell wall‐ and major CHO metabolism (Figure 3). Trehalose metabolism was also found to be different between the three genotypes. Hierarchical clustering of the genes enriched in these carbohydrate metabolic processes showed that the RIO/R9188 vs BTx406 difference focused on sucrose metabolism and starch synthesis related genes (Cluster G), whereas the RIO vs R9188/BTx406 difference reflected glycolysis, starch synthesis, sucrose metabolism, as well as cell wall degradation (Cluster A to E in Figure S12).

### T6P/SnRK1 regulated genes

**Figure 4:**
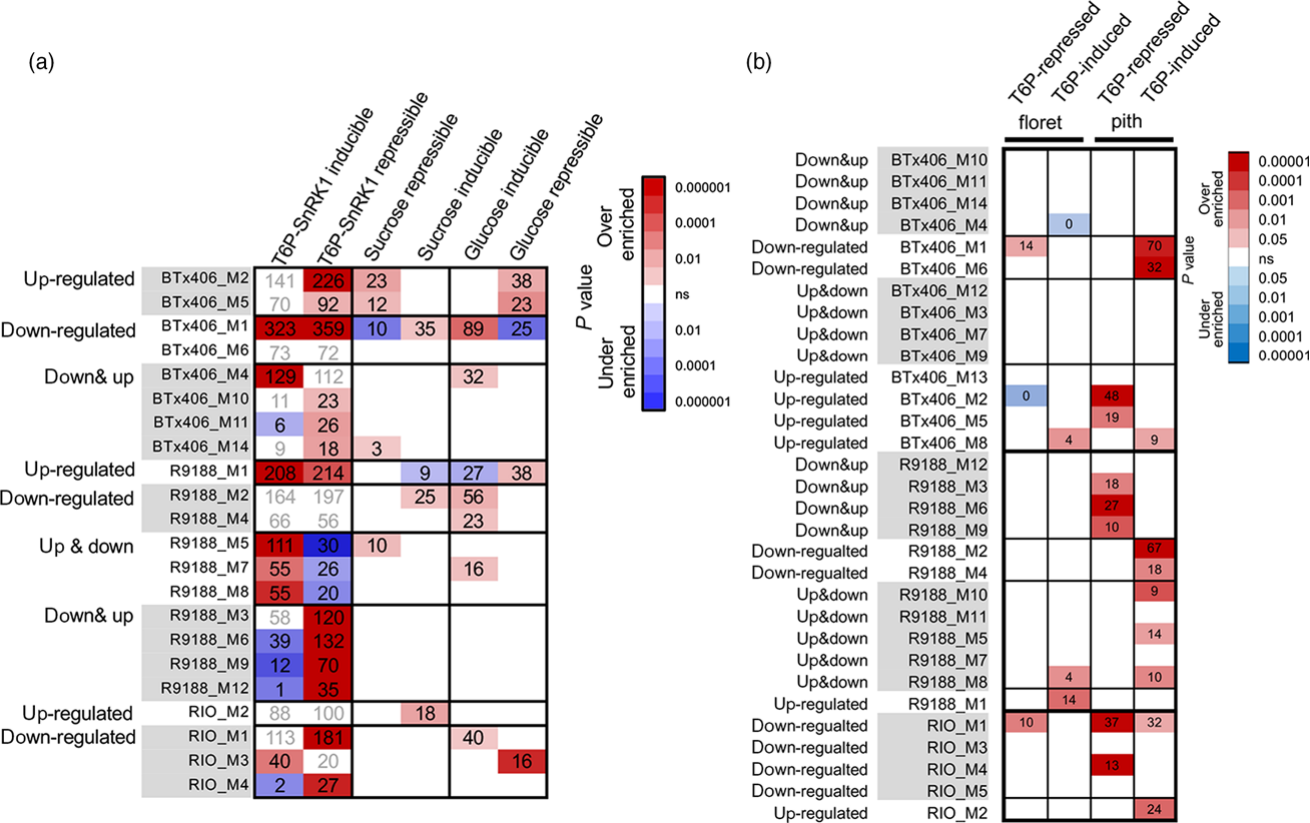
Highlighted overlaps between R9188 and BTx406 co‐expression modules and T6P/SnRK1 regulated gene sets from *Arabidopsis* (a) and maize (b) (*P*_hypergeomtric_ < 0.05).

As shown in transcriptome analysis, the trehalose pathway was differentially regulated between RIO and R9188/BTx406 (Figures 2 and 3). Given the role of T6P as a sucrose signal and a feedback regulator, and its regulation of SnRK1 (sucrose non‐fermenting 1‐related kinase 1) (Lunn *et al*., 2014), we asked whether the distinct patterns of transcriptome reprogramming between these genotypes could be partly contributed to T6P‐mediated signalling. Therefore, we compared sorghum co‐expression modules with several *Arabidopsis* gene sets (Data S6) and discovered that several modules in R9188 significantly overlapped with *Arabidopsis* gene sets (Figure S13). These public gene sets were generated independently by comparing transgenic events or mutants of *Arabidopsis* plants with various sugar starvation treatment conditions. Interestingly, the R9188 up‐and‐down regulated modules were significantly over‐enriched with T6P/SnRK1‐inducible genes but under‐enriched with T6P/SnRK1‐repressible genes. On the contrary, the down‐and‐up regulated modules in R9188 were over‐enriched with T6P/SnRK1‐repressible genes, but under‐enriched with T6P/SnRK1‐inducible genes (*P*_hypergeometric_ < 0.05; Figure 4a). Such relationships were not observed with *Arabidopsis* sucrose‐ and glucose‐responsive gene sets, demonstrating that the R9188 transcriptome was partially reprogrammed by the T6P/SnRK1‐mediated signalling pathway. We also compared sorghum co‐expression modules with maize gene sets generated from floret and pith of developing cob, which were generated with ectopic expression of rice *TREHALOSE PHOSPHATE PHOSPHATASE1* (*TPP1*; Oszvald *et al*., 2018). Significant overlaps were observed with the maize pith but not with floret gene sets (Figure 4b). Maize T6P‐inducible genes were enriched in down‐regulated modules from R9188/BTx406. In contrast with their enrichment in down‐regulated modules from RIO, Maize T6P‐repressible genes were enriched in the up‐ or down & up‐regulated modules of R9188/BTx406. Therefore, comparison of the sorghum co‐expression modules with several *Arabidopsis* and maize gene sets suggested that transcriptomes of R9188 and BTx406 were partly reprogrammed by the T6P signal. Given the known impacts of SnRK1 on metabolic genes (reviewed in Figueroa *et al*., 2016), we investigated changes of previously identified SnRK1 marker genes (Baena‐Gonzalez *et al*., 2007; Martinez‐Barajas *et al*., 2011; Oszvald *et al*., 2018; Zhang *et al*., 2009). Differential expression patterns of SnRK1 marker genes were similar between R9188 and BTx406 but not detected in RIO (Figure S14). Hierarchical clustering of metabolic‐related genes that are potentially regulated by T6P/SnRK1 identified several groups of genes with similar up‐ or down‐regulated patterns in R9188/BTx406 but different from their expression in RIO, suggesting T6P/SnRK1–mediated effects on metabolic pathways (Figure S15).

### Primary metabolic differences

**Figure 5:**
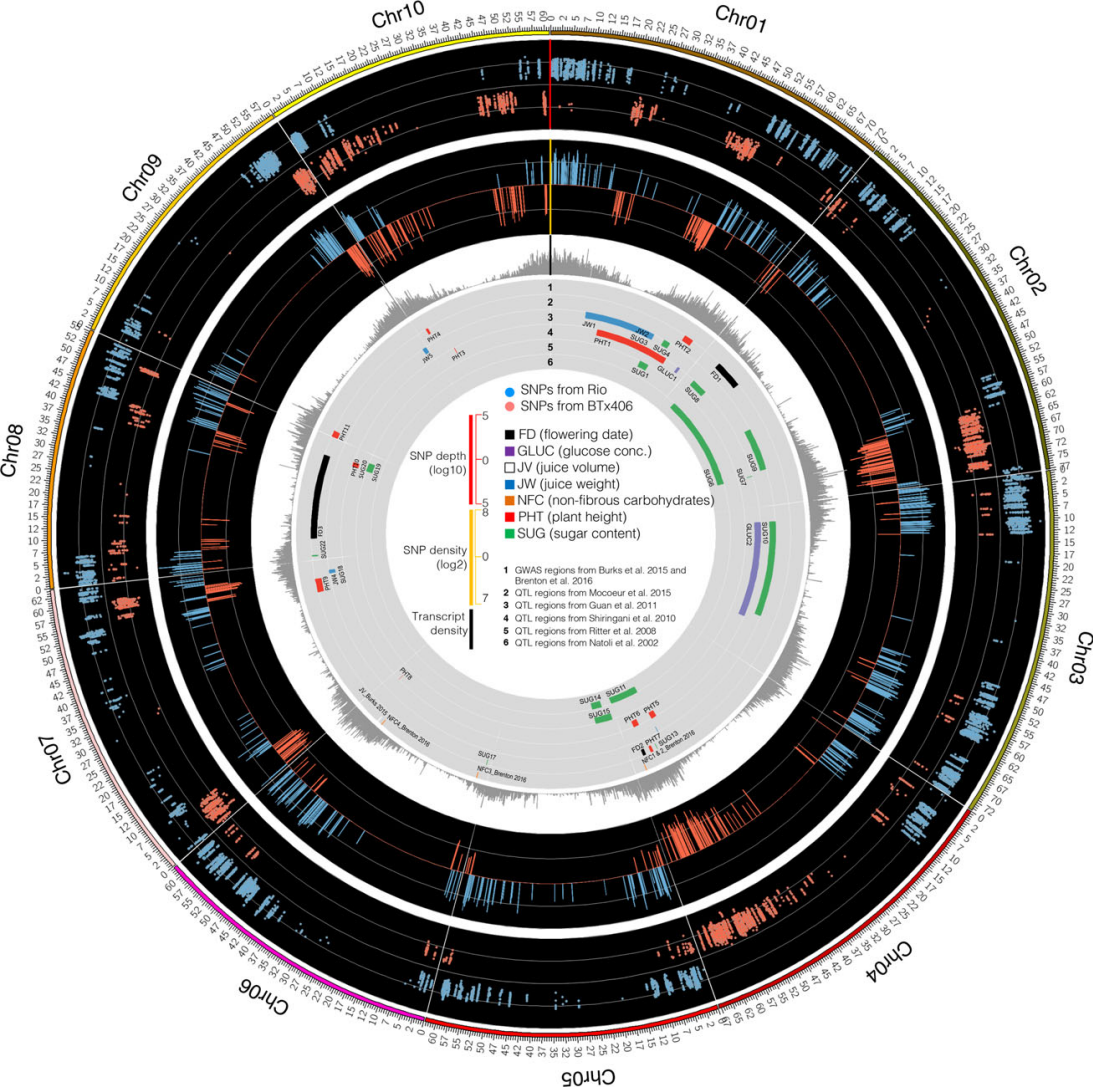
Overview of the introgressed regions in R9188 genome. Tracks (outer to inner) indicate SNP depths, parental genomic regions (100‐kb bin), transcript density, QTL/GWAS regions associated with sorghum traits previously identified (Table S11).

We further analysed expression dynamics of genes involved in sucrose, starch and cell wall metabolic pathways. Taking advantage of the genetic relationship between R9188 and their parents RIO and BTx406, we called single nucleotide polymorphisms (SNPs) using RNA‐seq data to identify BTx406 chromosomal regions introgressed in the R9188 genome. Comparing SNPs of R9188 with those of RIO and BTx406, we separated SNPs into two groups, group‐1 SNPs have the same alleles as RIO, whereas group‐2 SNPs have the same alleles as BTx406. Circos plot revealed multiple introgressions in R9188, mostly located on chromosome 1, 2, 4, 7, 9 and 10 (Figure 5; Data S7). Introgression bins covered 1805 genes, 1769 of them were expressed, with 1081 of them being DEGs. Comparison of introgressed DEGs with 1637 genes potentially regulated by T6P/SnRK1 pathway showed T6P‐regulated genes were significantly enriched (271 T6P‐regulated genes; *P*_hypergeometric_ = 1.79 e−30; Table S7). Therefore, introgressed alleles could explain differential T6P signalling. A total of 19 and 35 SNPs was detected from BTx406 and RIO, respectively, which were categorized as high‐effect variants. Aside from regions related to primary metabolic functions, a large number of 2596 and 2607 SNPs from BTx406 and RIO could have predicted moderate effects, respectively (Table S8).

**Figure 6:**
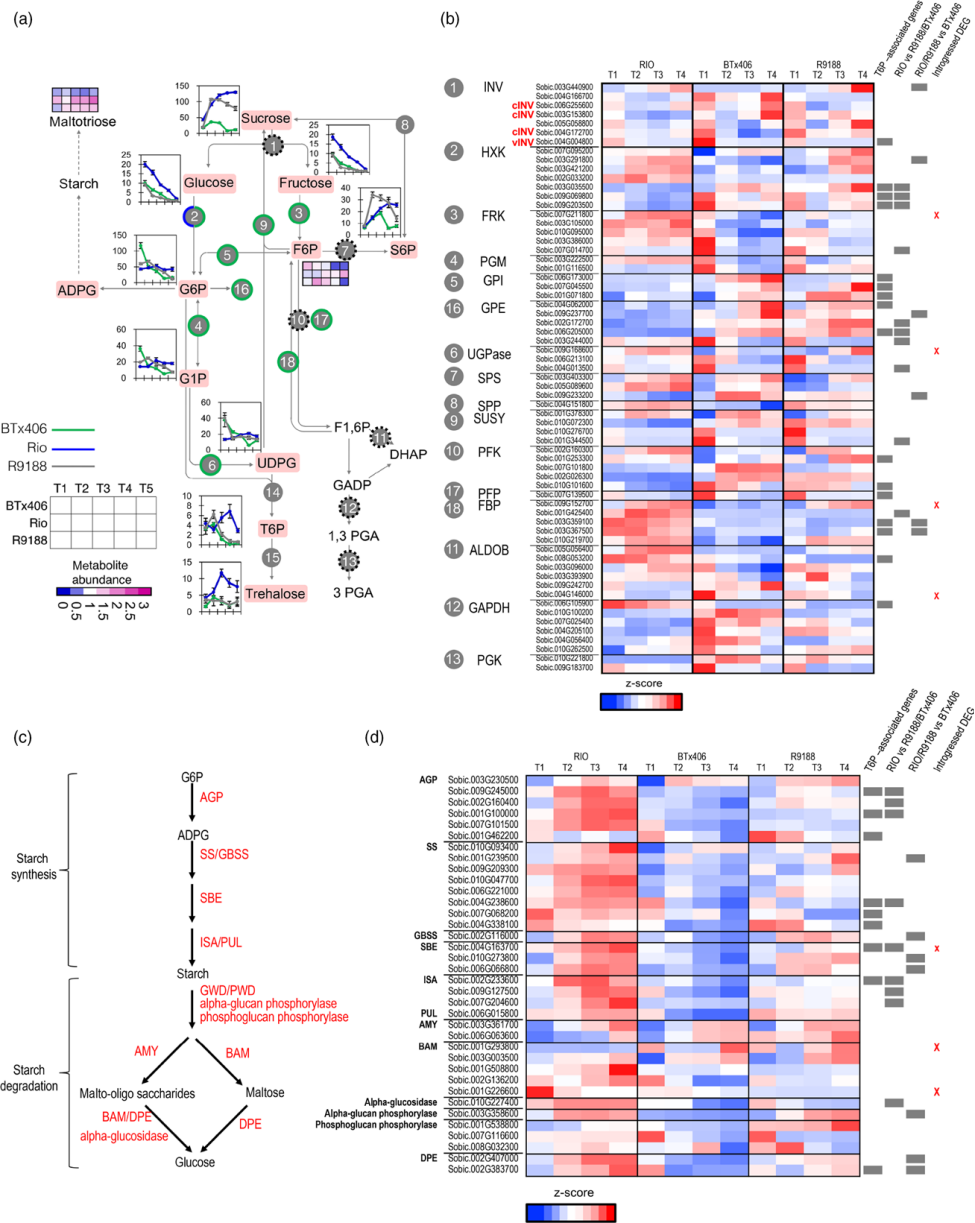
Transcriptomic and metabolic comparisons of sucrose (a, b) and starch metabolisms (c, d). Green circle: metabolic gene family in R9188 with expression patterns similar to BTx406. Half blue and half green circle indicates several *HXKs* in R9188 showing expression patterns similar to BTx406, whereas the remaining *HXKs* exhibited expression patterns similar to RIO. Black broken circle: the gene family showed distinct expression patterns between RIO, R9188 and BTx406. Gene expression levels were normalized by *z*‐score transformation across genotypes and time points.

Comparison of sucrose metabolic functions showed that several gene families involved in sugar phosphates metabolism exhibited similar patterns in R9188 and BTx406 but distinct patterns in RIO, including fructokinase (FRK), phosphoglucomutase (PGM), glucose‐6‐phosphate isomerase (GPI), glucose‐6‐phosphate 1‐epimerase (GPE) and UDP‐glucose pyrophosphorylase (UGPase) (Figure 6). Moreover, genes encoding diphosphate‐fructose‐6‐phosphate, 1‐phospho‐transferase (PFP) and fructose 1,6‐bisphosphatase (FBP) also had similar expression profiles in R9188 and BTx406, different from those in RIO. PFP and FBP are involved in glycolysis and gluconeogenesis, which are important for maintaining stable F6P pools. Indeed, these differences in gene expression pattern are consistent with the metabolome data. First, F6P was gradually increased in RIO but decreased in R9188 and BTx406 post‐anthesis, which could be explained by up‐regulated expression of FRK in RIO and decreased expression in R9188/BTx406 (Figure 6a). Second, G6P and G1P were stable over time in RIO, but were increased at T2 in R9188 before a decrease, with G6P and G1P sharply decreased in BTx406. Interestingly, among the seven differentially expressed *hexokinase* (*HXK*) genes, four *HXK*s had similar expression patterns between R9188 and BTx406, but distinct from RIO. Three of these *HXK*s could be regulated by T6P, similar like genes encoding GPI, GPE, PFK and FBP.

Genes involved in starch synthesis and degradation were up‐regulated and highly expressed in RIO, whereas only some starch synthetic (GBSS and SBE) and degradation genes (AMY, BAM, a‐glucan phosphorylase and phosphoglucan phosphorylase) were up‐regulated to some extent in R9188 (Figure 6c,d). Expression levels of most starch metabolic genes were down‐regulated in BTx406 compared with RIO and R9188. Expression data indicated that starch turnover was highly active in RIO followed by R9188, supported by maltotriose profiles (Figure 6a). Activation of starch metabolism could not be explained by introgressions because only three starch metabolic genes are in introgressed regions with low expression levels relative to their gene families. Still, eight genes in starch synthesis could be T6P‐regulated.

We also compared expression patterns of genes involved in primary cell wall synthesis, phenyl‐propanoid, mono‐lignol pathways, cell wall reassembly and degradation pathways (Rai *et al*., 2016; Figure S16a). Overall, R9188 showed similar expression patterns compared to BTx406 for most cell wall genes (Figure S16b). In contrast, RIO maintained higher or up‐regulated expression levels for several gene families. Particularly, compared to R9188/BTx406, up‐regulated or higher expression levels were observed in RIO for genes that belong to cellulose, hemicellulose, pectin synthesis and mono‐lignol pathways (HCT, CCR, C3H, C4H, F5H). Genes involved in cell wall (gene families GH9, GH17 and GH26) and polysaccharide degradation (beta‐glucosidases) were also highly expressed in RIO but decreased in R9188/BTx406. Introgressed DEGs tend to map to biosynthetic genes, such as hemicellulose, phenyl‐propanoid and mono‐lignol pathways, suggesting that cell wall synthesis might be repressed partly due BTx406 alleles. Furthermore, T6P‐regulated genes mostly belong to lignin synthesis and cell wall degradation families (GH17, GH28, GH35). Therefore, RIO, but not R9188 and BTx406, maintained highly active cell wall synthesis and degradation.

### Candidate genes regulating T6P pathway

In our analysis, co‐expression modules and data on T6P‐regulated genes in *Arabidopsis* and maize are consistent with the roles of T6P signalling on sucrose degradation, starch synthesis and cell wall metabolism. Then, which genes could cause the opposite trend of T6P between RIO and R9188/BTx406? T6P is converted from UDPG and G6P by T6P synthase (TPS) and then catalysed into trehalose by TPP (Lunn *et al*., 2014). We identified 11 *SbTPS* and 12 *SbTPP* genes (Figures S15, S17). Phylogenetic analysis showed that *TPS* genes were divided into two groups, the catalytic class‐I group (clade B) and non‐catalytic class‐II group (clade A; Figure S17). Based on the consistency between functional complementation experiments in yeast and the TPS domain analyses in *Arabidopsis*, rice and kiwifruit (Vandesteene *et al*., 2012; Voogd *et al*., 2016; Zang *et al*., 2011), domain analysis showed that only one *SbTPS* gene (Sobic.009G200200) was likely to be functional, because all its amino acids essential for substrate binding were highly conserved in *Arabidopsis*, rice and maize (Vandesteene *et al*., 2010; Zang *et al*., 2011), whereas the important amino acids in the remaining nine *SbTPS*s had numerous substitutions (Table S9). All *SbTPP* genes were highly conserved both in the three motifs required for TPP activity and other catalytically important amino acids (Figure S18), suggesting that they are catalytically active. In addition, no SNPs with predicted high and moderate effects were found in *TPSs* and *TPPs*.

Although the catalytic *TPS* gene displayed expression patterns not correlated with T6P dynamics, lowly expressed and down‐regulated in RIO, but up‐regulated in BTx406/R9188 (Figure 1g), TPS also could be regulated at the protein level. First, TPSs are subjected to differential phosphorylation in *Arabidopsis* (Glinski and Weckwerth, 2005) and *in vivo* phosphorylation of regulatory TPSs controls their interactions with the 14‐3‐3 protein (Harthill *et al*., 2006). Second, TPSs are involved in protein complex formation both in *Arabidopsis* and rice (Geelen *et al*., 2007; Zang *et al*., 2011). By contrast, the C/S1 bZIP regulates the TPP genes at the transcriptional level in *Arabidopsis*, hence controlling catalysis of T6P into trehalose to adjust levels of sucrose signalling (Ma *et al*., 2011). Indeed, expression levels of several *TPP*s were in accordance with T6P abundance comparing RIO with BTx406/R9188 (*TPPA.2*, Sobic.002G303900; *TPPB2.4*, Sobic.001G353300; *TPPB1.1*, Sobic.007G124200; Figure S19). Moreover, we found a *TPP* allele introgressed from BTx406 (*TPPA.2*). Distinct expression patterns of *TPP* genes could well explain the opposite trend of T6P.

We also searched for candidate genes that could have metabolic impacts in R9188/BTx406. If genes that participate in sucrose signalling (T6P) and/or primary metabolism are differentially regulated between grain and sweet sorghum, then introgression of these genes from grain sorghum (BTx406) into R9188 should reflect changes in primary metabolism or signalling networks. We applied stringent filtering steps to find differentially responsive DEGs that were not only introgressed into R9188 but also well‐correlated with T6P signal. A candidate gene list was generated containing 32 T6P‐associated genes with distinct patterns between RIO and R9188/BTx406 (Table S10), with 29 of them annotated. We grouped the 29 genes into seven categories, of which 12 were related to metabolism, eight involved in transcriptional and post‐transcriptional regulation, and three involved in transport. Candidate genes comprise transcription factors (seven TFs in 32 genes, *P*_hypergeometric_ = 0.009), including one C‐group *bZIP* (Sobic.008G157100) that mapped to the introgression regions of R9188 (Wang *et al*., 2011). It has been reported that the C/S1 groups of bZIP proteins form heterodimers and trans‐activate downstream genes, which control plant energy metabolism and nutrient allocation (Dietrich *et al*., 2011; Hartmann *et al*., 2015; Mair *et al*., 2015; Weltmeier *et al*., 2006, 2009; Zhang *et al*., 2015, 2016). *TPP* genes are regulated by C/S1 bZIP at the transcriptional level in *Arabidopsis* (Ma *et al*., 2011). Indeed, we identified two C‐group *bZIPs* (Sobic.010G218500, Sobic.008G157100), of which the expression profiles were correlated with *TPP* genes (Ehlert *et al*., 2006; Peviani *et al*., 2016; Figure S19). Besides, one *MYB* and two *NAC*s were introgressed and differentially expressed between RIO and BTx406/R9188. Both *MYB* and *NAC* homologs in *Arabidopsis* and maize regulate secondary cell wall deposition and components (Zhong *et al*., 2008, 2011). Their homologs in maize also affect starch metabolism in endosperm (Huang *et al*., 2016). Furthermore, several candidate genes involved in metabolism were introgressed and associated with the T6P signal in R9188/BTx406. This includes a cellulose synthase‐like (CSL) gene that was highly expressed and up‐regulated in RIO but not in R9188/BTx406, possibly controlling mixed‐linkage glucan synthesis in mature stems (Vega‐Sanchez *et al*., 2012). Another candidate gene functioning in BCAA and asparagine catabolism was homologous to the *Arabidopsis 3‐methylcrotonyl‐CoA carboxylase alpha* (*MCCA*), which is not only directly involved in BCAA catabolism but also induce pleiotropic changes in amino acid metabolism (Ding *et al*., 2012). We also found two candidates related to nitrogen metabolism, a *RWP‐RK* transcription factor and a β*‐ureidopropionase* gene; the former could transactivate downstream targets to control nitrate signalling and assimilation (Konishi and Yanagisawa, 2013), whereas the latter functioned in recycling of pyrimidine nitrogen to general nitrogen metabolism (Zrenner *et al*., 2009). Lastly, an introgressed and T6P‐associated gene encoding a G6P translocator, was highly expressed and up‐regulated in RIO but up‐ and down‐regulated in R9188/BTx406, of which the homolog in *Arabidopsis* transports G6P from cytoplasm into chloroplast to fuel starch synthesis (Dingenen *et al*., 2016; Dyson *et al*., 2014, 2015).

### Sugar transporters

Three gene families encoding sucrose transporters have been implicated in sugar accumulation in sorghum, including H^+^/sucrose antiporters located on tonoplast TSTs (Bihmidine *et al*., 2016), H^+^/sucrose symporters SUTs (Bihmidine *et al*., 2015; Leach *et al*., 2017; Milne *et al*., 2013), and the clade III of SWEET (Eom *et al*., 2015; Mizuno *et al*., 2016). We compared expression patterns of these gene families between the three genotypes (Figure S20). Among the differentially expressed SUTs, *SbSUT1* (Sobic.001G488700) and *SbSUT2* (Sobic.008G193300) were highly expressed with *SbSUT2* expression in RIO being significantly higher than in R9188/BTx406. For TST, *SbTST1* (Sobic.001G312900) and *SbTST4‐1* (Sobic.004G099300) were highly expressed. Particularly, *SbTST4‐1* expression in RIO was dramatically higher than those in R9188/BTx406. Comparison of *SbSUT2* and *SbTST4‐1* expression patterns between the three genotypes, together with similar expression profiles in other sweet sorghum lines (Bihmidine *et al*., 2015; Mizuno *et al*., 2016), strongly indicated roles in sucrose import for stem storage sink (Figure S20). For *SWEET*s, 16 genes were expressed with Sb*SWEET7‐1* (Sobic.007G191200) introgressed from BTx406 into R9188. Surprisingly, expression levels of seven *SWEET* genes in RIO were lower than those in R9188/BTx406, including three highly expressed ones (Sobic.004G136600, Sobic.008G094000 and Sobic.009G080900). *SbSWEET3‐7* (Sobic.003G269300) was the only one showing higher and up‐regulated expression in RIO than in R9188/BTx406. Generally, most of the *SWEET*s in R9188 showed similar expression patterns as BTx406 but not RIO. Considering substrate specificity of clade III SWEETs and previously‐reported expression profiles, we highlighted a few potential candidates that need further investigation.

## Discussion

Nonstructural carbohydrate accumulation in sweet sorghum provides a valuable resource for low‐cost, high‐efficiency conversion of biomass to biofuels and bioproducts. Therefore, a systematic understanding of metabolic and molecular mechanisms controlling this process is of great significance. Still, several difficulties could hinder progress. Transcriptome itself might be insufficient to predict primary metabolism as transcriptome‐metabolome discordance has been shown due to complex layers of regulations (Fernie and Stitt, 2012; Schwender *et al*., 2014). Useful comparisons between sweet sorghum and other sorghum ideotypes (e.g., grain sorghum) are lacking, but they could be complicated by the fact that sweet sorghum experiences considerably different environment, especially day length, due to late flowering. Furthermore, comparison between two phenotypically distinct sorghum varieties would solely rely on correlation with sugar traits. We overcame these problems, sampling stem tissues from different internodes for simultaneous transcriptome and metabolome analysis of features associated with sugar accumulation. In addition, samples were derived from three genetically related genotypes (BTx406, RIO and R9188). To obtain a systematic overview of primary metabolism and identify candidate genes effectively, we used two independent but complementary approaches to analyse the data. Considering different environmental effects on gene expression between genotypes, the first approach displayed expression dynamics for each genotype by co‐expression networks, which permitted categorizing modules of enriched biological functions. The second approach comparing expression between genotypes within the same time points could directly highlight differences in RIO vs R9188/BTx406 and in RIO/R9188 vs BTx406. Nevertheless, it is worth mentioning that caution should be taken when results were interpreted from direct comparisons of gene expression between RIO and R9188/BTx406 at T4 or T5 due to the colder environment that late‐flowering RIO experienced.

**Figure 7:**
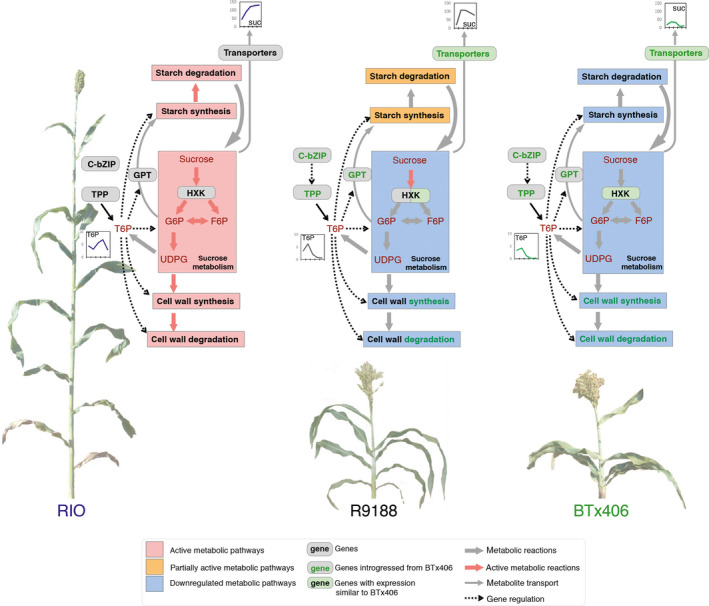
Schematic model of sugar accumulation and sink‐strength establishment in internodes between RIO, BTx406 and R9188. Starch metabolism is fully active in RIO, partially active in R9188 and down‐regulated in BTx406. Cell wall metabolism is active in RIO, but not in R9188 and BTx406, partly due to T6P signal and introgressed haplotypes of metabolic genes. Sucrose metabolism is active in RIO to maintain sugar phosphate pools, but down‐regulated in R9188 and BTx406 except for the HXK family. Metabolites are labeled in scarlet font. Genes and metabolic pathways are shown in black font, introgression in green font.

Combination of aforementioned strategies and approaches led to a representative graph (Figure 7). RIO is active in sucrose degradation, and starch and cell wall synthesis and degradation, whereas R9188 maintains partially active starch but decreased cell wall metabolism. The BTx406 internode has the lowest primary metabolic activities. Decreased concentrations of glucose and fructose and stable expression of cytosolic INVs in all three genotypes suggests carbohydrates tend to transfer from glucose/fructose to sucrose possibly from SPS and SUSY (Figure 6). This trend is consistent with the difference in sucrose concentration, which could be explained from three aspects. (i) RIO has higher glucose/fructose concentration. (ii) RIO has higher TST expression to uptake sucrose into vacuoles. (iii) RIO has higher flow of sugar phosphates to maintain sucrose demand and support active downstream metabolism. Indeed, most sucrose degradation genes in R9188 maintaining sugar phosphates pools resembled expression patterns in BTx406 except for three HXKs, showing similar expression dynamics in RIO (Figure 6b). These data are consistent with metabolic profiles, where RIO maintained stable sugar phosphate pools, whereas concentrations of several sugar phosphates were gradually decreased in R9188/BTx406. However, this observation is hardly explained by introgression of only four BTx406 haplotypes encoding sucrose‐metabolic enzymes, expression at low levels. Differences in sugar phosphate pools between genotypes are in line with starch and cell wall metabolisms, as ADPG and UDPG serve as precursors for starch and cellulose/hemicellulose synthesis, respectively. Starch synthesis and degradation appear to be active in RIO and partially active in R9188, supported by gene families of starch synthesis and degradation in R9188, with intermediate expression levels compared to RIO and BTx406 (Figure 6). Furthermore, starch catabolic product maltotriose was highest in RIO, followed by R9188 and significantly lower in BTx406 (Figure 6). It seems that decreased expression of several starch synthetic genes in R9188 could be explained by opposite T6P signals between RIO vs R9188/BTx406. R9188 and BTx406 exhibited similar expression trends for cell wall metabolism (Figure S16). Post‐anthesis down‐regulation of genes controlling cellulose, hemicellulose, phenyl‐propanoid and mono‐lignol synthesis in R9188/BTx406 suggests active cell wall metabolism in mature internodes may be associated with sugar accumulation in RIO. Indeed, a GWAS study using a panel of sweet and biomass accessions indicated genetics trade‐off between structural and non‐structural carbohydrates allocation (Brenton *et al*., 2016). In short, the schematic model depicts an orchestration of carbohydrate allocation and metabolic pathways due to different internode sink strength in the three genotypes, with sucrose accumulation as reflection of the strongest sink strength in RIO.

We observed opposite T6P signals and differences in hundreds of genes potentially regulated by T6P between RIO and R9188/BTx406 (Figures 4, S14, S15). We therefore sought to identify key genes responsible for the inverse T6P signal and found differential regulation of the trehalose pathway as one of the control points in sink‐strength regulation of sweet sorghum. T6P has come to be recognized as an endogenous signal and negative feedback regulator of sucrose levels. Modifying T6P content changes sucrose levels, induces transcriptome reprogramming, alters sugar, starch and amino acid metabolisms, and influences plant development by affecting carbon allocation (Schluepmann *et al*. 2003, reviewed in Figueroa and Lunn, 2016). To date, the proposed scenario for the T6P‐sucrose nexus in sugar signalling is that T6P exhibits bidirectional regulation with sucrose and the SnRK1 pathway. T6P can inhibit SnRK1 activity and T6P could have a synergistic inhibition together with G6P and/or G1P (Nunes *et al*., 2013). Evidence suggests that SnRK1 could regulate TPS by phosphorylation and activate TPP transcription through the C/S1 group bZIPs (Harthill *et al*., 2006; Ma *et al*., 2011). T6P might also affect its own downstream genes, which partly overlaps with SnRK1 downstream genes. Decreasing T6P in maize by *OsTPP1* ecotopic expression led to down‐regulation of primary metabolism and up‐regulation of secondary metabolism in a tissue‐dependent manner (Nuccio *et al*., 2015; Oszvald *et al*., 2018). Although the T6P‐sucrose nexus has been proposed to be critical for maintaining sucrose homeostasis in a spatial‐temporal specific manner (Figueroa *et al*., 2016; Lunn *et al*., 2014), genetic approaches have not been able to obtain a full understanding of its mode‐of‐action because of dissecting primary from secondary, long‐term effects of T6P.

Here, results suggest that action of a T6P‐sucrose nexus is distinct between RIO and R9188/BTx406. Differences in T6P dynamics could determine in part sink strength and sugar accumulation in sweet sorghum stems. Furthermore, our observation in R9188, based on overlapping patterns with *Arabidopsis* and maize gene sets (Figures 4, S13), implies that the T6P signal in R9188 was turned over possibly by either BTx406‐introgressed candidates, such as the *TPP* and *bZIP* genes, or differentially expressed *TPS*,* TPP* and C‐group *bZIP*s. Still, this analysis might possibly underestimate the numbers of genes regulated by the T6P/SnRK1‐pathways due to (i) limited numbers of gene IDs convertible to *Arabidopsis* gene IDs, (ii) evolutionary convergence and divergence in sugar signalling between the three species, (iii) tissue‐dependent effects of T6P and the different tissues/systems used in the present study (Oszvald *et al*., 2018; Wingler *et al*., 2012; Zhang *et al*., 2009), (iv) technical difficulties in separating primary effects from secondary, long‐term or adaptive effects.

Differences in primary metabolism between RIO and BTx406/R9188 could be attributed to introgressed genes covering several functional categories, including cell wall metabolism, translocator for starch synthesis, nitrogen and amino acid metabolism, and regulation of T6P signalling. For example, homologs of MCCA and a G6P translocator in *Arabidopsis* have been proposed to impact amino acid and starch metabolism, respectively (Ding *et al*., 2012; Dingenen *et al*., 2016; Dyson *et al*., 2014, 2015). Many genes involved in cell wall synthesis and degradation were introgressed from BTx406 into R9188, coinciding with down‐regulation in both genotypes (Figure S16). Still, metabolic contributions and associations with T6P signal need further investigation. In addition, it is yet to be established whether the T6P‐signal candidate genes (TPPs and C‐group bZIPs) and the enrichment of T6P‐regulated genes in introgressed regions could result in the T6P‐mediated transcriptome responses between RIO and R9188/BTx406.

The systematic view of internode sugar accumulation and the candidate genes highlighted here provide the starting point for validation of their functions and modification of stem composition. The orchestration of several metabolic pathways and involvement of T6P‐signalling suggest new strategies for engineering primary metabolism in sorghum stem. Given the broad genetic diversity in sorghum germplasm, targeted sequencing of the candidate genes suggested in the present study will reveal their allelic diversity and possible associations with bioenergy traits, and hence may facilitate breeding and engineering for stem composition.

## Experimental procedures

### Plant materials and field experiments

Three sorghum genotypes, BTx406, RIO and R9188, were used. BTx406 is early‐flowering and dwarf, whereas RIO is late‐flowering and tall with high contents of soluble sugars accumulating in its stem. R9188 is a dwarf converted line of which the early maturing and dwarf loci were introgressed from BTx406 (Ritter, 2007; Figure S2; Appendix S1). The field experiments were conducted at Rutgers in 2014, using a split plot design with three replicates. The genotypes were randomly assigned to plots within each block, with subplot randomly assigned to sampling time points. Five time points were used: flag leaf stage (T1), 100% flowering (T2), 10 days after flowering (T3), 15 days after flowering (T4) and 30 days after flowering (T5).

### Metabolomics

For targeted metabolic profiling, quantitative results of fourteen compounds including sugars and sugar phosphates were analysed by three different panels over four different chromatographic systems developed by Metabolon, Inc. under a contract with Syngenta. The untargeted metabolomic analysis platform is composed of four independent platforms: UHPLC‐MS/MS optimized for basic species, UHPLC‐MS/MS optimized for acidic species, polar LC platform (UHPLC(HILIC)‐MS/MS) and GC‐MS (Evans *et al*., 2009; Ohta *et al*., 2009; Appendix S1).

### Transcriptome analyses

Twelve internode samples representing three genotypes with four time points (T1, T2, T3 and T4), three biological replicates were used for RNA‐seq analysis. Total RNA was extracted with TRIzol reagent and RNA‐seq (150‐bp, pair‐ends) were performed using standard Illumina Nextseq 500 platform protocols. Two complementary approaches comparing transcriptome data between the three genotypes were used. Co‐expression networks for each genotype were constructed independently using the R package WGCNA (Langfelder and Horvath, 2008, 2012; Langfelder *et al*., 2011). Gene functional enrichment analysis was performed using R package ‘clusterProfiler’ (*P *<* *0.05 and *q *<* *0.2; Yu *et al*., 2012) with sorghum annotation information collected from several sources, gene ontology (GO; Du *et al*., 2010), MapMan (Usadel *et al*., 2009), KEGG and Plant Metabolic Network (PMN; Chae *et al*., 2014), Sorghum Functional Genomics Database (Tian *et al*., 2016), transcription factor and targeted gene annotations (Jin *et al*., 2014; Riano‐Pachon *et al*., 2007; Yilmaz *et al*., 2009), and curated starch/sucrose metabolism genes (Campbell *et al*., 2016). The TPS and TPP gene families in sorghum were identified by searching Phytozome database and BLAST with maize sequences (Henry *et al*., 2014). The SNPs were called for each genotype by comparing with the reference genome (BTx623, Sbicolor_v2.1_255), using SAMTools with default parameters (Li, 2011). After filtering, homogeneous, homozygous SNPs were compared between the three genotypes to determine the introgressed regions in R9188 (Details in Appendix S1). The SNPs and sugar traits‐related QTL regions identified previously (Table S11, Appendix S3) were visualized in Figure 5.

## Conflict of interest

The authors declare no conflict of interest.

## Authors’ contributions

Y.L., W.W., P.W., N.B. and J.M. conceived and designed the experiments. Y.L. and W.W. planned and performed the experiments. P.W. and N.B. contributed to metabolome analysis. Y.L., W.W., M.T. and Y.F. contributed to data analysis and interpretation. Y.L. and J.M. drafted the manuscript and all authors critically revised and approved the final version of the manuscript.

## Supporting information


**Figure S1** Diagram of a representative plot in the field experiment.
**Figure S2** Photos of the three genotypes used in this study.
**Figure S3** Classification of the identified metabolites in non‐targeted metabolome analysis.
**Figure S4** PRelative metabolite abundances showed high correlations with the abundances measured by targeted method.
**Figure S5** A representative map of central metabolism in sorghum stem.
**Figure S6** Correlations of amino acids abundances in BTx406 (a), RIO (b) and R9188 (c).
**Figure S7** Characterization of co‐expression network modules.
**Figure S8** Robustness analysis of gene modules.
**Figure S9** Distributions of module membership (kME) for genes within each module.
**Figure S10** Functional enrichment analyses of co‐expression modules for RIO, R9188 and BTx406 by using GO (a, b), KEGG (c) and Plant Metabolic Network (d) annotations.
**Figure S11** Identification of DEGs representing the genotypic differences of RIO vs R9188/BTx406, RIO/R9188 vs BTx406 and RIO vs R9188 vs BTx406 by using upset plot visualization.
**Figure S12** Hierarchical clustering of the genes associated with enriched major CHO functional terms by using the second analysis approach.
**Figure S13** Pair‐wise comparison of sorghum co‐expression modules with public available gene sets responsive/regulated by sugar signalling in *Arabidopsis*.
**Figure S14** SnRK1 marker gene expression in RIO compared to BTx406 and R9188.
**Figure S15** (a) Heat map of the DEGs in trehalose biosynthetic pathway. (b) Hierarchical clustering of T6P‐regulated genes which are associated with primary metabolism and sugar transport.
**Figure S16** Expression profiles of cell wall related genes in sorghum.
**Figure S17** Phylogenetic analysis of TPS (a) and TPP (b) genes from sorghum (blue), maize (red), rice (green) and *Arabidopsis* (black).
**Figure S18** Multiple sequence alignment for the TPP gene family revealed high conservation in the TPP domain.
**Figure S19** Expression profiles of the C/S1 groups of bZIP and correlation analysis of the TPP family and C/S1 groups of bZIP in sorghum.
**Figure S20** Expression profiles of sugar transporter genes during sugar accumulation (a) and a hypothetic model illustrates the roles of different sugar transporters in sorghum internode (b).
**Table S1** Sorghum trait descriptions.
**Table S2** Quality control (QC) precision values of the 14 compounds analysed by targeted metabolic profiling.
**Table S3** Correlation of bioreplicates of the metabolome samples.
**Table S4** Summary of RNA‐seq mapping results.
**Table S5** Pairwise correlations of bioreplicates used in the RNA‐seq analysis.
**Table S6** Numbers of expressed genes detected in each genotype and time point.
**Table S7** Number of overlapping genes between introgressed DEGs and R9188 DEGs which might potentially be regulated by the T6P/SnRK1 signalling network.
**Table S8** Distribution of the SNP effects predicted from BTx406 SNPs and RIO SNPs.
**Appendix S1** Supplemental methods and references.

**Table S9** Analysis of the TPS and Haloacid Dehalogenase (HAD) domains and conserved amino acids required for TPS activities in deduced TPS proteins from *E. coli*, yeast, *Arabidopsis*, rice, maize and sorghum.
**Table S10** Details of forty‐seven differentially expressed candidate genes identified in the introgressed regions of R9188 with expression levels and annotation information.
**Table S11** Information about QTLs associated with plant height, flowering time, glucose concentration, juice volume, juice weight, non‐fibrous carbohydrates and sugar contents, which were identified by previous studies.

**Data S1** Raw data of sugars and sugar phosphates from the stems of three sorghum genotypes (RIO, BTx406 and R9188) measured by targeted metabolic profiling.
**Data S2** Median normalized metabolome data from the stem of three sorghum genotypes (RIO, BTx406 and R9188) measured by untargeted metabolome platforms.
**Data S3** Gene expression levels used for differential expression analysis and WGCNA, including log2‐transformed (RPKM+1) values, fold change of gene expression between time points and q value determined by both DEseq and edgeR.
**Data S4** An integrated sorghum genome annotation file based on sorghum genome v2.1. This annotation file includes an integrated, non‐redundant GO annotations from Phytozome and AgriGO, a hierarchical MapMan annotation, a transcription factor annotation, and annotations for cell wall related genes, hormone –related genes.
**Data S5** Comparison of annotation for starch metabolic genes between three different sources, including KEGG, Plant Metabolic Networks (PMN), and researcher curated gene list. Based on the comparison, a consensus annotation for starch metabolic genes were generated and used for RNAseq data analysis.
**Data S6** Arabidopsis gene sets and merged non‐redundant gene sets which were responsive to or regulated by T6P/SnRK1, sucrose and glucose.
**Data S7** The SNPs for defining introgression regions in R9188.
