# Supplementary material for: Transcriptome and metabolome reveal distinct carbon allocation patterns during internode sugar accumulation in different sorghum genotypes
Source: Plant Biotechnol J. 2018 Sep 15;17(2):472–87. doi: 10.1111/pbi.12991 (PMC6335075; doi:10.1111/pbi.12991)
Supplement: Supplementary file 1 — Figure S1 Diagram of a representative plot in the field experiment. Figure S2 Photos of the three genotypes used in this study. Figure S3 Classification of the identified metabolites in non‐targeted metabolome analysis. Figure S4 PRelative metabolite abundances showed high correlations with the abundances measured by targeted method. Figure S5 A representative map of central metabolism in sorghum stem. Figure S6 Correlations of amino acids abundances in BTx406 (a), RIO (b) and R9188 (c). Figure S7 Characterization of co‐expression network modules. Figure S8 Robustness analysis of gene modules. Figure S9 Distributions of module membership (kME) for genes within each module. Figure S10 Functional enrichment analyses of co‐expression modules for RIO, R9188 and BTx406 by using GO (a, b), KEGG (c) and Plant Metabolic Network (d) annotations. Figure S11 Identification of DEGs representing the genotypic differences of RIO vs R9188/BTx406, RIO/R9188 vs BTx406 and RIO vs R9188 vs BTx406 by using upset plot visualization. Figure S12 Hierarchical clustering of the genes associated with enriched major CHO functional terms by using the second analysis approach. Figure S13 Pair‐wise comparison of sorghum co‐expression modules with public available gene sets responsive/regulated by sugar signalling in Arabidopsis. Figure S14 SnRK1 marker gene expression in RIO compared to BTx406 and R9188. Figure S15 (a) Heat map of the DEGs in trehalose biosynthetic pathway. (b) Hierarchical clustering of T6P‐regulated genes which are associated with primary metabolism and sugar transport. Figure S16 Expression profiles of cell wall related genes in sorghum. Figure S17 Phylogenetic analysis of TPS (a) and TPP (b) genes from sorghum (blue), maize (red), rice (green) and Arabidopsis (black). Figure S18 Multiple sequence alignment for the TPP gene family revealed high conservation in the TPP domain. Figure S19 Expression profiles of the C/S1 groups of bZIP and correlation analysis of the TPP family [file PBI-17-472-s002.docx]

**Transcriptome and metabolome reveal distinct carbon allocation patterns during internode sugar accumulation in different sorghum genotypes**

Yin Li^1^, WenqinWang^1,2^, Yaping Feng^1^, Min Tu^1^, Peter E. Wittich^3^, Nicholas J. Bate^3^, Joachim Messing^1,*^

^1^Waksman Institute of Microbiology, Rutgers, The State University of New Jersey, Piscataway, NJ 08854, USA.

^2^present address: School of Agriculture and Biology, Shanghai Jiaotong University, 800 Dong Chuan Road, Shanghai 200240, China.

^3^Syngenta Crop Protection, LLC, 9 Davis Drive, Research Triangle Park, North Carolina 27709, USA.

* Correspondence: messing@waksman.rutgers.edu

**Running head:** transcriptome and metabolome in sorghum internode

**Supplemental Figures:**

**Figure S1** Diagram of a representative plot in the field experiment.

**Figure S2** Photos of the three genotypes used in this study.

**Figure S3** Classification of the identified metabolites in non-targeted metabolome analysis.

**Figure S4** Relative metabolite abundances showed high correlations with the abundances measured by targeted method.

**Figure S5** A representative map of central metabolism in sorghum stem.

**Figure S6** Correlations of amino acids abundances in BTx406 (A), RIO (B) and R9188 (C).

**Figure S7** Characterization of co-expression network modules.

**Figure S8** Robustness analysis of gene modules.

**Figure S9** The distributions of module membership (kME) for genes within each module.

**Figure S10** Functional enrichment analyses of co-expression modules for RIO, R9188 and BTx406 by using GO (a, b), KEGG (c) and Plant Metabolic Network (d) annotations.

**Figure S11** Identification of DEGs representing the genotypic differences of RIO vs R9188/BTx406, RIO/R9188 vs BTx406 and RIO vs R9188 vs BTx406 by using upset plot visualization.

**Figure S12** Hierarchical clustering of the genes associated with enriched major CHO functional terms by using the second analysis approach.

**Figure S13** Pair-wise comparison of the sorghum co-expression modules with public available gene sets responsive/regulated by sugar signaling in *Arabidopsis*.

**Figure S14** SnRK1 marker gene expression in RIO compared to BTx406 and R9188.

**Figure S15** Heat map of the DEGs in trehalose biosynthetic pathway. **(b)** Hierarchical clustering of T6P-regulated genes which are associated with primary metabolism and sugar transport.

**Figure S16** Expression profiles of cell wall related genes in sorghum.

**Figure S17.** Phylogenetic analysis of TPS (a) and TPP (b) genes from sorghum (blue), maize (red), rice (green) and *Arabidopsis* (black).

**Figure S18.** Multiple sequence alignment for the TPP gene family revealed high conservation in the TPP domain.

**Figure S19.** Expression profiles of the C/S1 groups of bZIP and correlation analysis of the TPP family and C/S1 groups of bZIP in sorghum.

**Figure S20** Expression profiles of sugar transporter genes during sugar accumulation (a) and a hypothetic model illustrates the roles of different sugar transporters in sorghum internode (b).

**Supplemental Tables:**

**Table S1** Sorghum trait descriptions.

**Table S2** Quality control (QC) precision values of the 14 compounds analyzed by targeted metabolic profiling.

**Table S3** Correlation of bioreplicates of the metabolome samples.

**Table S4** Summary of RNA-seq mapping results.

**Table S5** Pairwise correlations of bioreplicates used in the RNA-seq analysis.

**Table S6** Numbers of expressed genes detected in each genotype and time point.

**Table S7** Number of overlapping genes between introgressed DEGs and R9188 DEGs which might potentially be regulated by the T6P/SnRK1 signaling network.

**Table S8** Distribution of the SNP effects predicted from BTx406 SNPs and RIO SNPs.

**Table S9** Analysis of the TPS and Haloacid Dehalogenase (HAD) domains and conserved amino acids required for TPS activities in the deduced TPS proteins from *E.coli*, yeast, Arabidopsis, rice, maize and sorghum.

**Table S10** Details of twenty-nine differentially-expressed candidate genes that were associate with T6P and located in the introgression region of R9188.

**Table S11** Information about QTLs associated with plant height, flowering time, glucose concentration, juice volume, juice weight, non-fibrous carbohydrates and sugar contents, which were identified by previous studies.

**Supplemental Datasets.**

**Data S1** raw data of sugars and sugar phosphates from the stems of three sorghum genotypes (RIO, BTx406 and R9188) measured by targeted metabolic profiling.

**Data S2** median normalized metabolome data from the stem of three sorghum genotypes (RIO, BTx406 and R9188) measured by untargeted metabolome platforms.

**Data S3** Gene expression levels used for differential expression analysis and WGCNA, including log2-transformed (RPKM+1) values, fold change of gene expression between time points and q value determined by both DEseq and edgeR.

**Data S4** An integrated sorghum genome annotation file based on sorghum genome v2.1. This annotation file includes an integrated, non-redundant GO annotations from Phytozome and AgriGO, a hierarchical MapMan annotation, a transcription factor annotation, and annotations for cell wall related genes, hormone –related genes.

**Data S5** Comparison of annotation for starch metabolic genes between three different sources, including KEGG, Plant Metabolic Networks (PMN), and researcher curated gene list. Based on the comparison, a consensus annotation for starch metabolic genes were generated and used for RNAseq data analysis.

**Data S6** Arabidopsis gene sets and merged non-redundant gene sets which were responsive to or regulated by T6P/SnRK1, sucrose and glucose.

**Data S7** The SNPs for defining introgression regions in R9188.

**Supplemental Figures.**


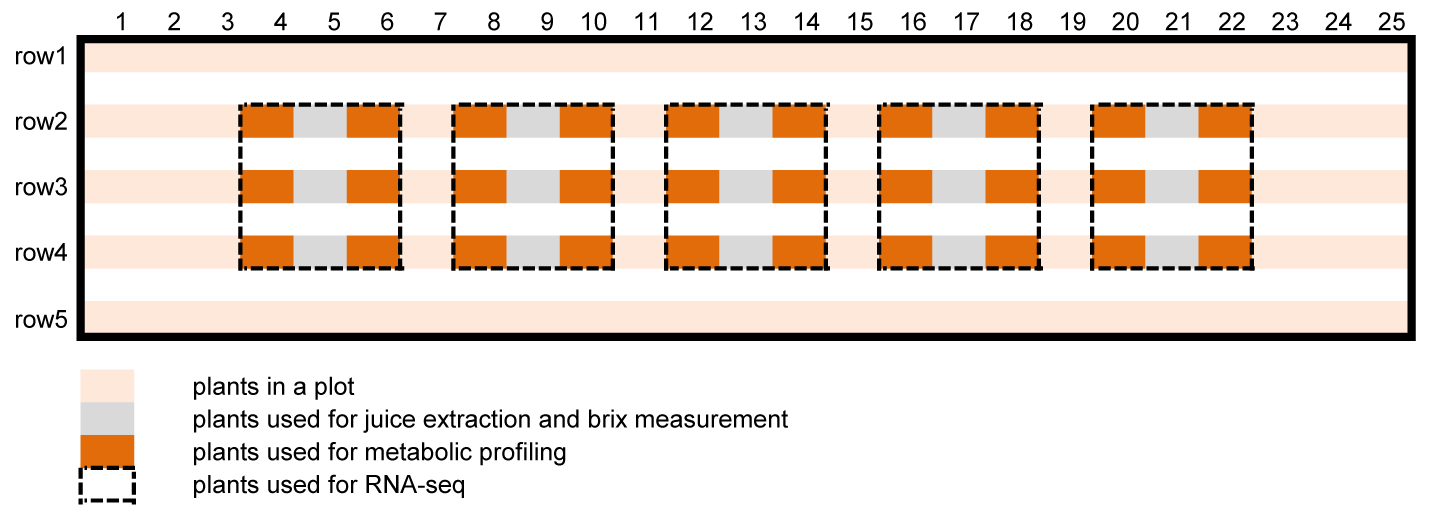


**Figure S1. Diagram of a representative plot in the field experiment.**

One plot consisted of 5 rows, with 25 plants per row. The central 3 rows were divided into 5 subplots and used for collecting samples for metabolome and transcriptome analyses. Among the 9 plants from each subplot, stems from the central 3 plants were harvested and used for Brix measurement.


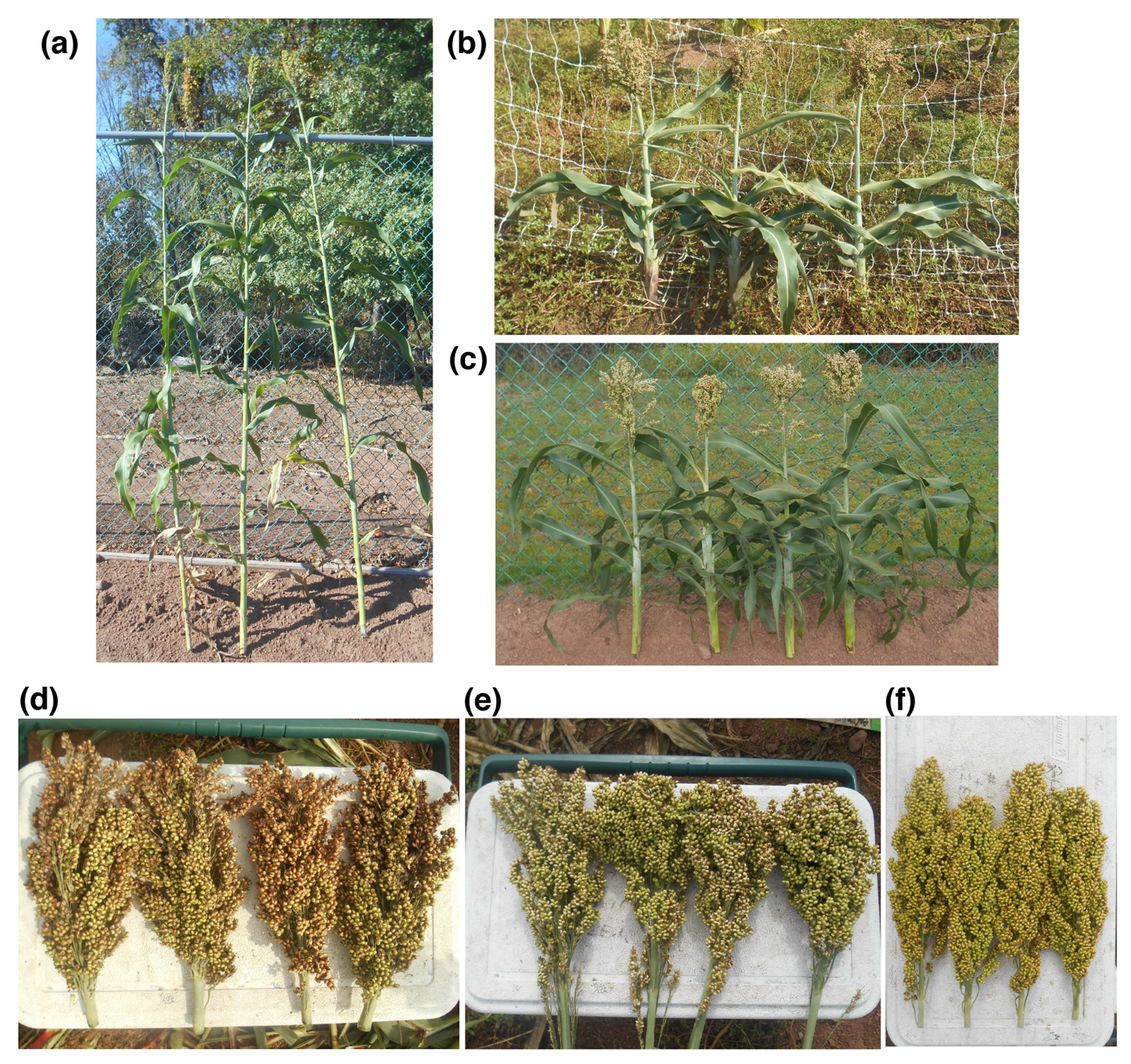


**Figure S2. Photos of the three genotypes used in this study: sweet sorghum RIO at 15 days after flowering (a), BTx406 at 30 days after flowering (b), R9188 at 30 days after flowering (c), and the panicles of BTx406 (d), R9188 (e) and RIO (f) at 30 days after flowering.**


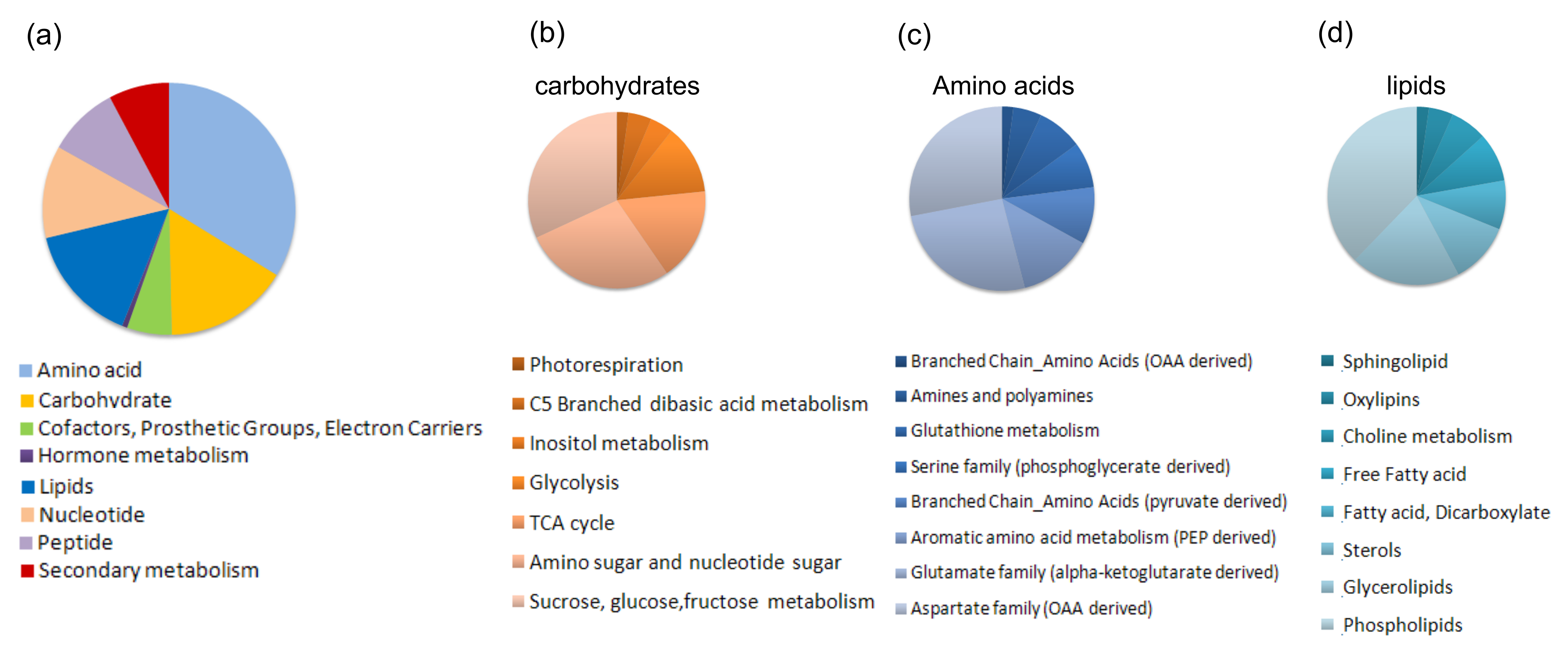


**Figure S3. Classification of the identified metabolites in non-targeted metabolome analysis.**

**(a)** The known metabolites fell into eight categories, including amino acids, carbohydrates, cofactors, hormones, lipids, nucleotides, peptides and secondary metabolites. Pie charts showed the pathways covered by the metabolites from the three major categories: carbohydrates **(b)**, amino acids **(c)** and lipids **(d)**.


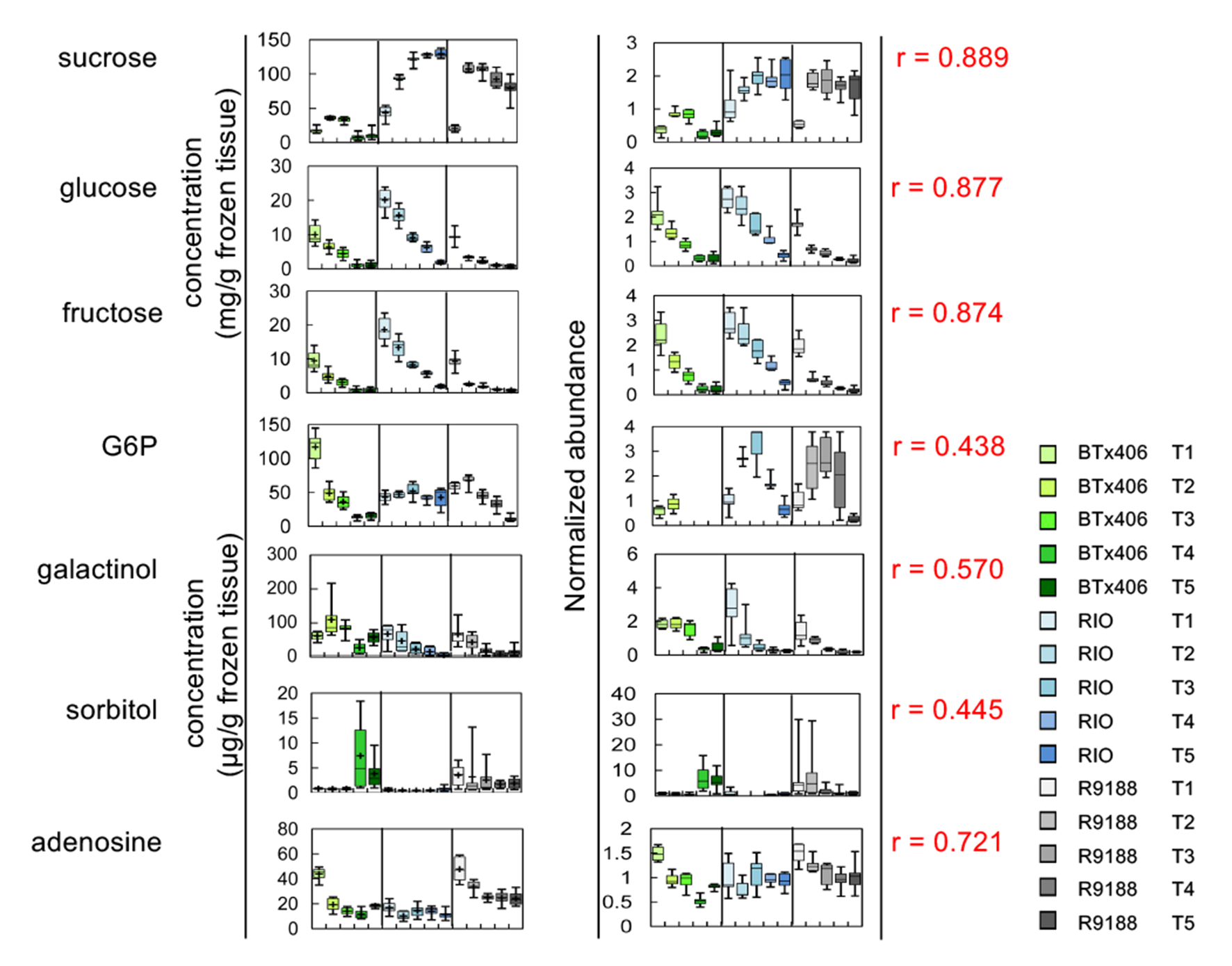


**Figure S4. Relative metabolite abundances showed high correlations with the abundances measured by targeted method.**

Seven metabolites which were measured by both targeted metabolic profiling and untargeted metabolome were used as an indication for result consistency. The concentrations of metabolites measured by targeted metabolic profiling are shown on the left panel, while the levels of the metabolites determined by untargeted metabolomics are shown on the right panel. Pearson’s correlations between targeted metabolic profiles and untargeted metabolome data were calculated using the averages of metabolite abundance per time point and genotype over all the samples. The abundance of metabolites from BTx406, RIO, R9188 are shown in green, blue and grey, respectively. (G6P, glucose 6-phosphate)


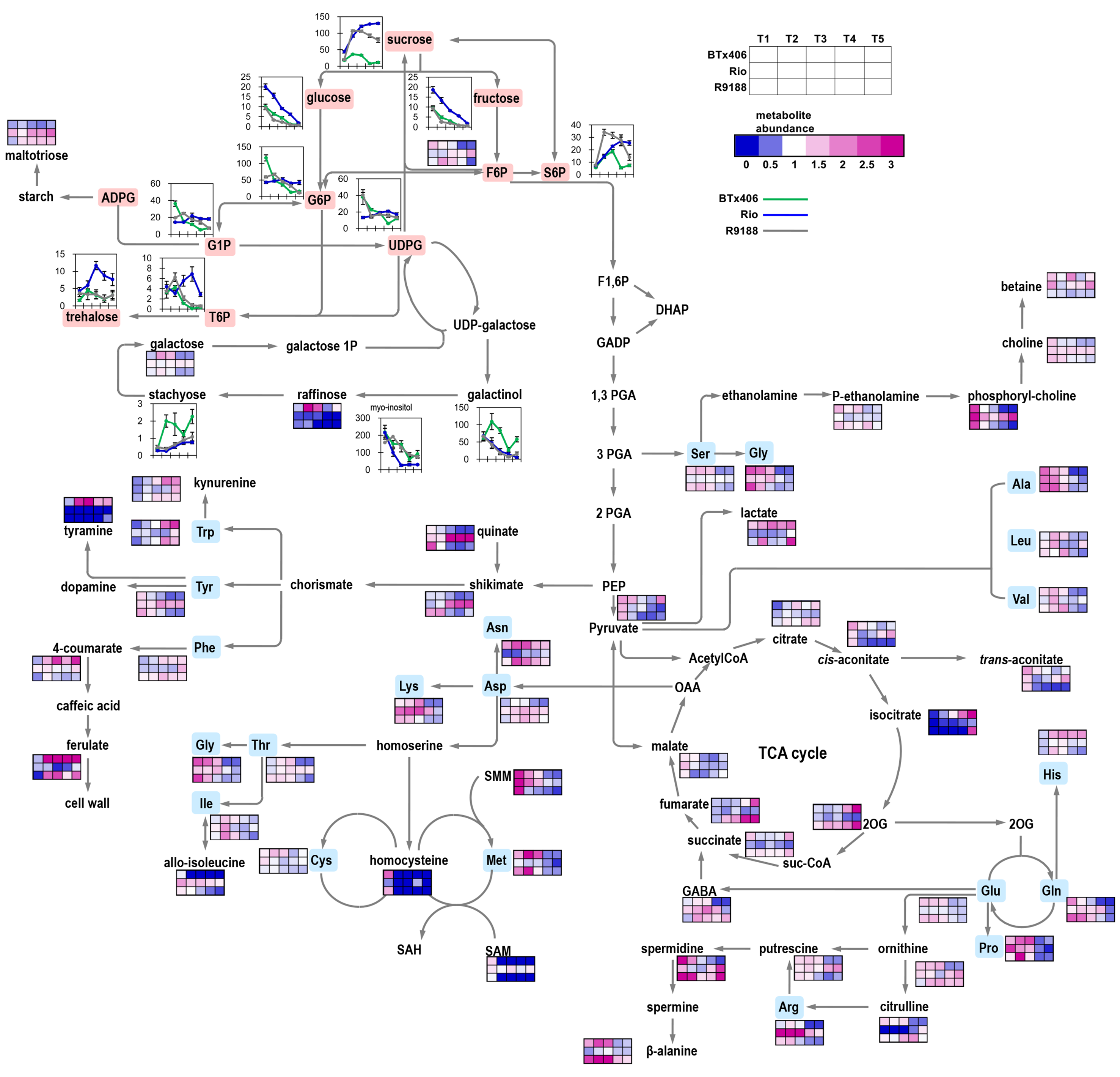


**Figure S5. A representative map of central metabolism in sorghum stem.**

A total of 67 metabolites were measure by targeted metabolic profiling and untargeted metabololome method, consisting of major sugars and sugar intermediates (highlighted in red box), amino acids (highlighted in blue box) and organic acids in tricarboxylic acid (TCA) cycle. The abundances of metabolites measured by targeted method were shown in line charts, with the same color codes to indicate different genotypes as in Figure 1. The abundances of metabolites measured by untargeted method were shown in heatmap.

Metabolite abbreviations: 1,3-PGA, 1,3-bisphosphoglyceric acid; 2-OG, 2-oxoglutarate; 3-PGA, glycerate 3-phosphate; 2-PGA, glycerate 2-phosphate; ADPG, adenosine diphosphate glucose; DHAP, dihydroxyacetone phosphate; F6P, fructose 6-phosphate; F1,6P, fructose 1,6-bisphosphate; GABA, gamma-aminobutyric acid; GADP, glyceraldehyde 3-phosphate; G1P, glucose 1-phosphate; G6P, glucose 6-phosphate; OAA, oxaloacetic acid; PEP, phosphoenolpyruvate; S6P, sucrose 6-phosphate; SAH, S-Adenosyl homocysteine; SAM, S-Adenosyl methionine; SMM, S-Methylmethionine; Suc-CoA, Succinyl-Coenzyme A; T6P, trehalose 6-phosphate; UDPG, uridine diphosphate glucose.


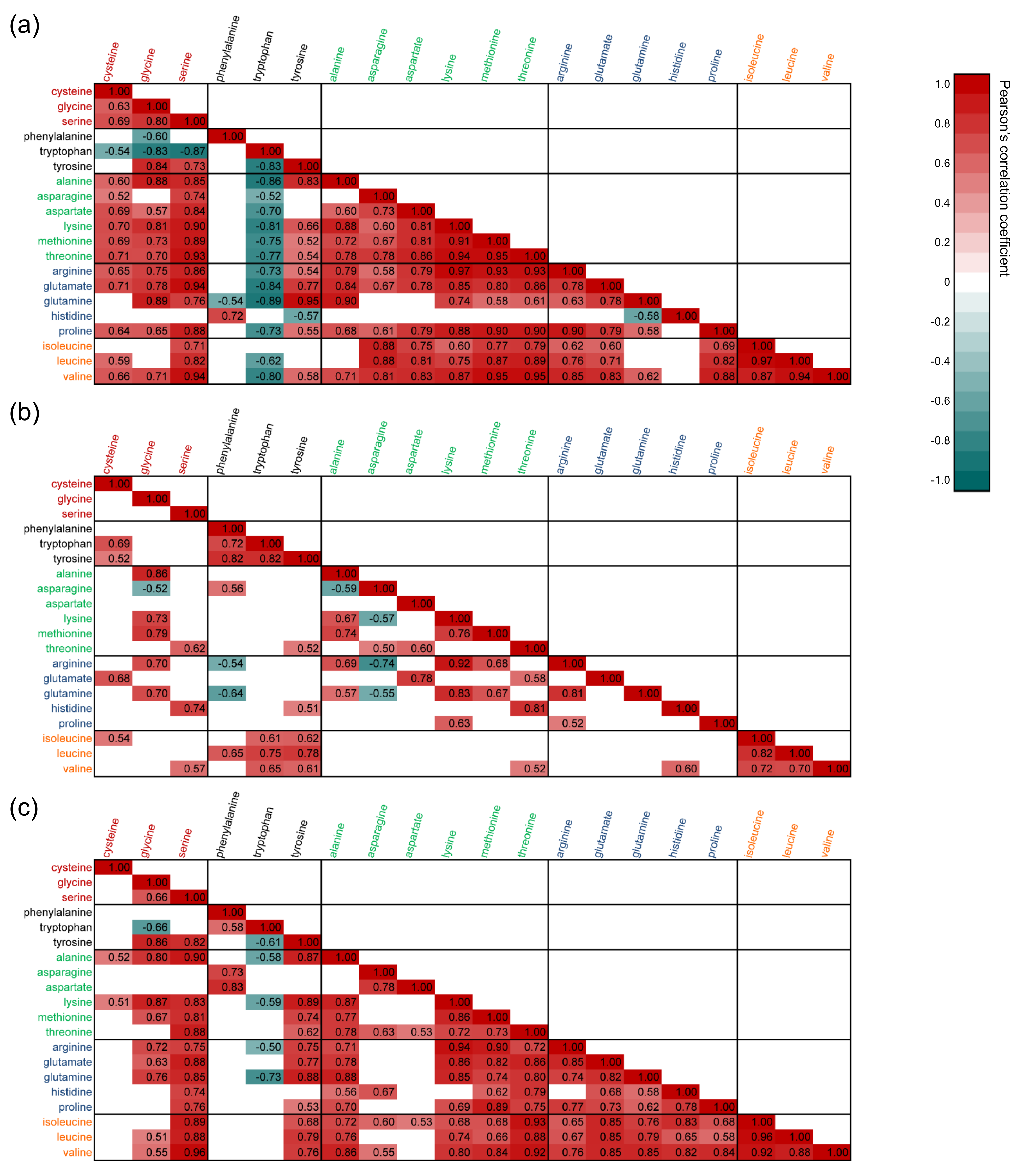


**Figure S6. Correlations of amino acids abundances in BTx406 (a), RIO (b) and R9188 (c).**

Amino acids are color-coded based on their metabolic pathways. Serine family (cysteine, glycine and serine) are presented in dark red; Aromatic amino acids (phenylalanine, tryptophan and tyrosine) are presented in black; aspartate family (alanine, asparagine, aspartate, lysine, methionine and threonine) are presented in green; glutamate family (arginine, glutamate, glutamine, histidine and proline) are presented in blue; branched-chain amino acids (isoleucine, leucine and valine) are presented in orange. Only those with absolute values of correlation coefficients more than 0.5 and significantly correlated are shown, while white cells indicate the correlation were not significant or the absolute values of correlation coefficient <0.5.


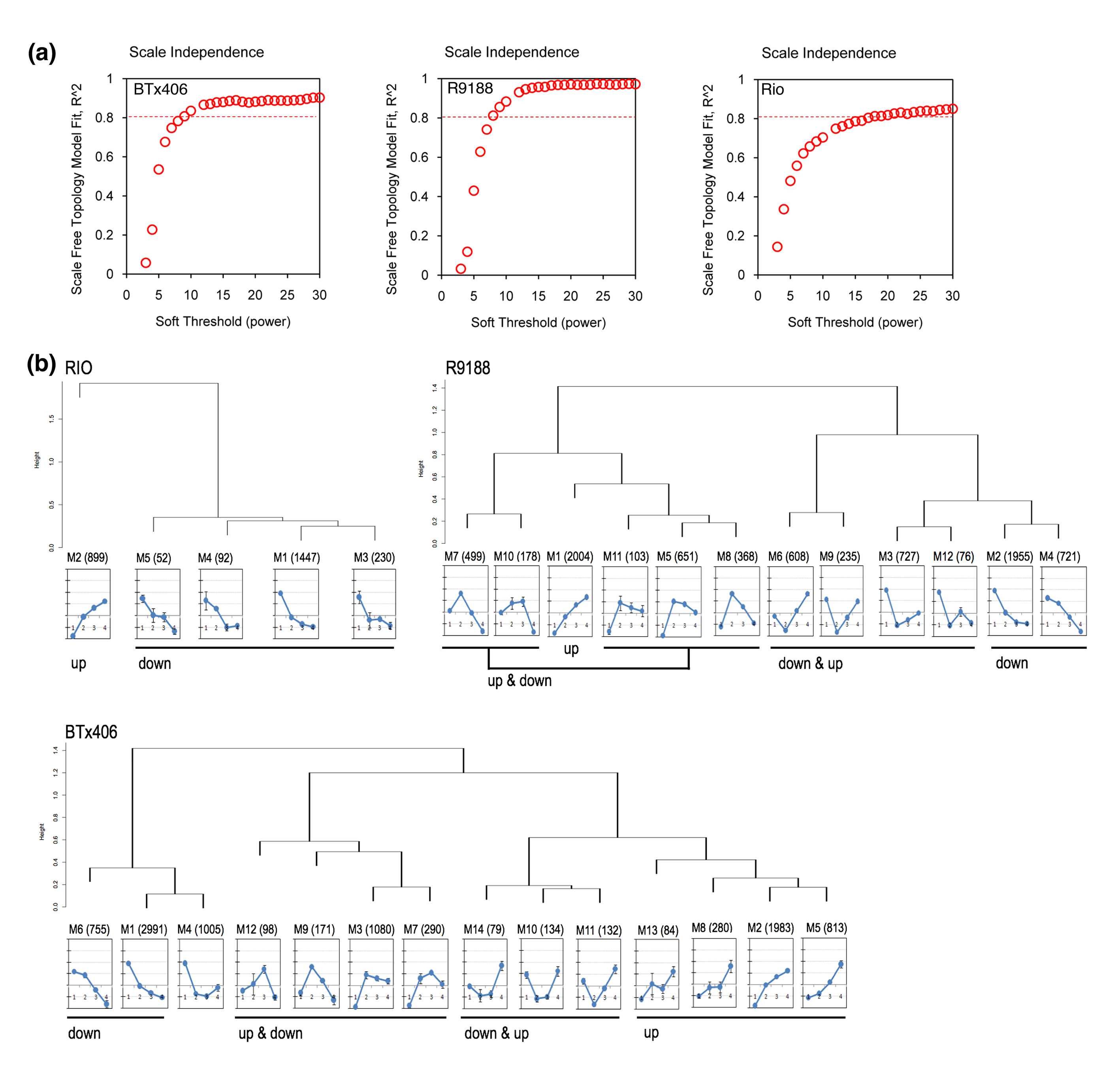


**Figure S7. Characterization of co-expression network modules.**

**(a)** Selection of soft threshold (power) for the construction of weighted gene co-expression network for each genotype. After raising the co-expression network to a power threshold, the network topology is approximately scale-free, with a coefficient of determination to scale-free topology being over 0.8.

**(b)** Hierarchical clustering of the co-expression modules in each genotype. Based on the coefficients of correlation between module eigengenes and hierarchical clustering, modules were further grouped into four expression tendencies: up-regulation, down-regulation, up and down- regulation, and down and up-regulation.


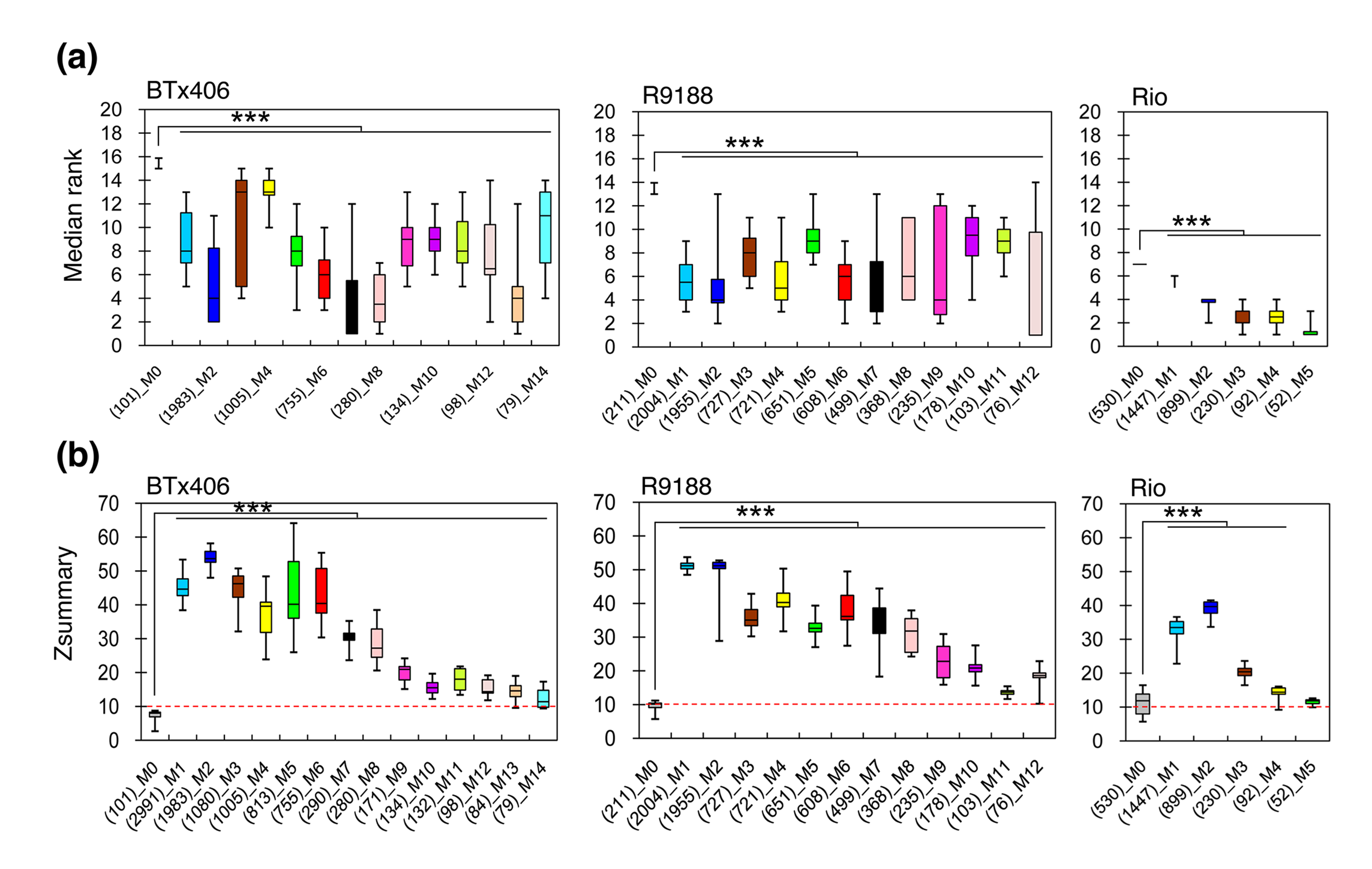


**Figure S8. Robustness analysis of gene modules.**

**(A)** Median rank, a module size-independent robustness parameter, were calculated and plotted with 20 times random resampling of 83% of the RNA-seq datasets in each genotype. Statistical analysis showed that the median rank of each module is significantly lower (*P* < 0.001) than that of the non-module genes (M0) in all three genotypes.

**(B)** Z_summary_, a module size-dependent robustness parameter in which module density preservation and connectivity preservation are both taken into consideration, were calculated and plotted with 20 times random resampling of 83% of the RNA-seq datasets in each genotype. Results showed that the Z_summary_ of each module implied strong module preservation (Z_summary_ > 10). Statistical analysis showed that the Z_summary_ of each module is significantly higher (*P* < 0.001) than that of the non-module genes (M0) in all three genotypes except for the smallest module in RIO (M5).


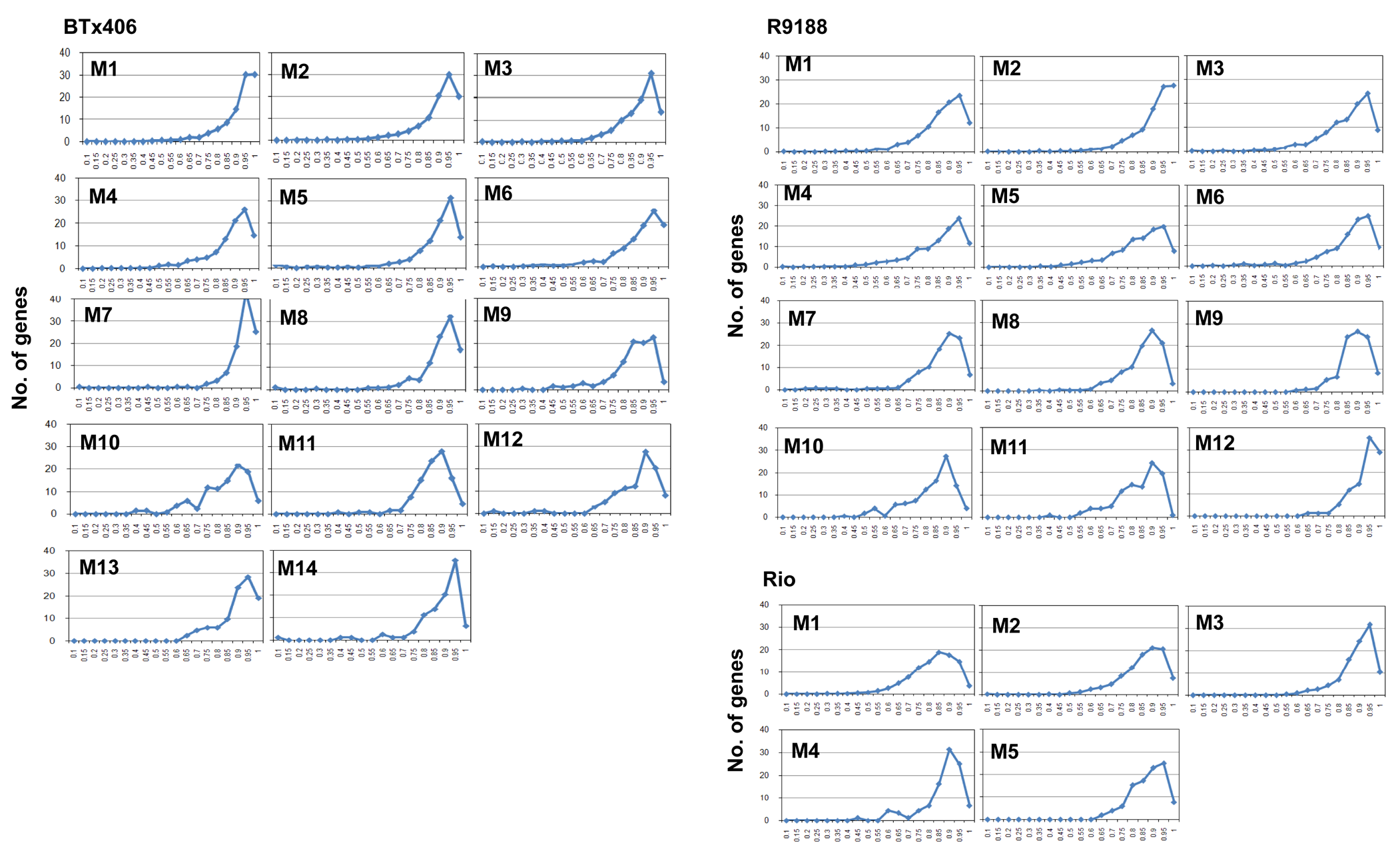


**Figure S9. The distributions of module membership (kME) for genes within each module.**

Module membership is also known as correlation-based intramodular connectivity. Visualization of kME distribution in each module allowed us to select the representative genes in each module, which were defined as modular genes, had a kME ≥ 0.7 and were well correlated with module eigengene. The selected modular genes were further used for functional enrichment analysis.


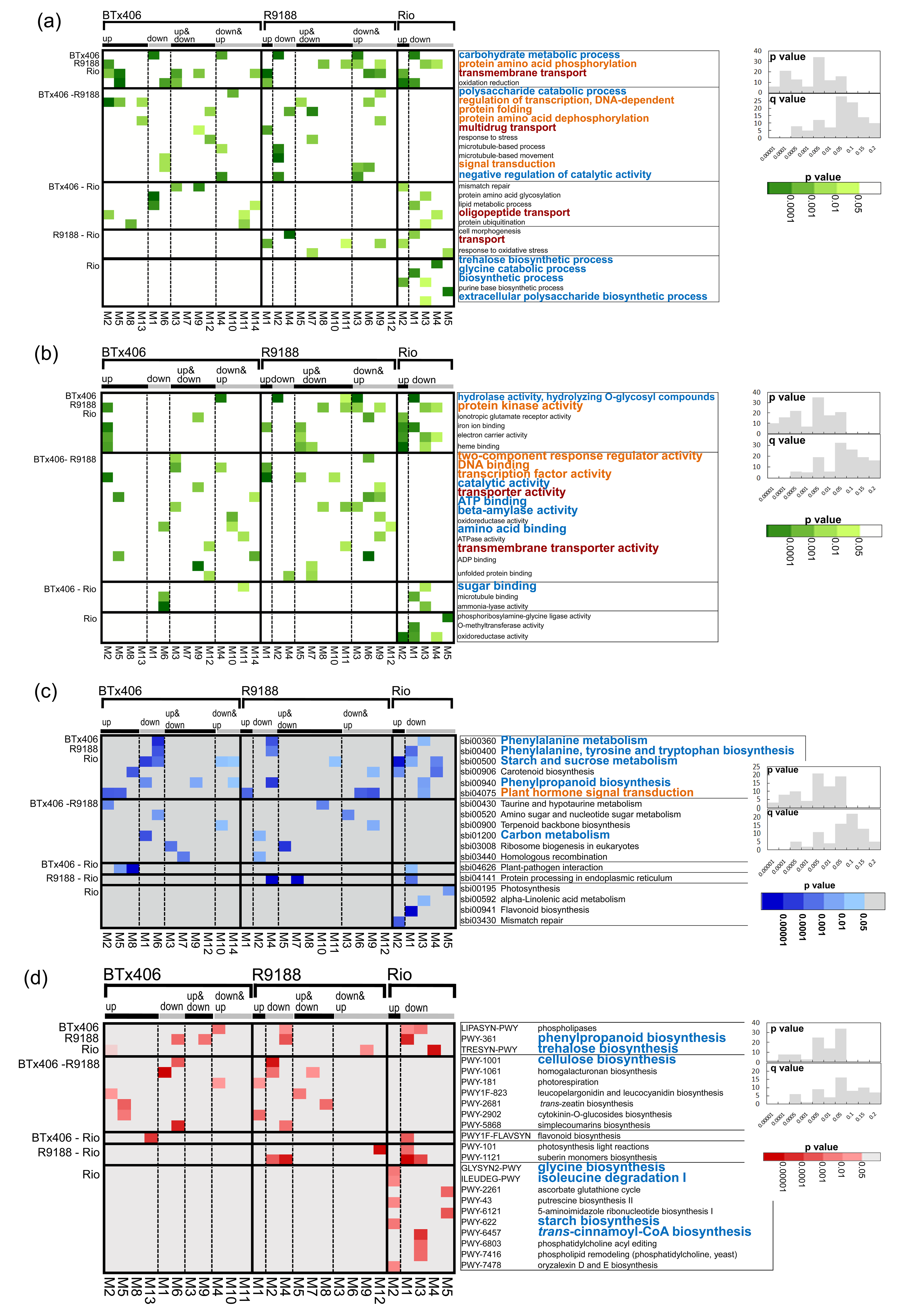


**Figure S10 Functional enrichment analyses of co-expression modules for RIO, R9188 and BTx406 by using GO (a, b), KEGG (c) and Plant Metabolic Network (d) annotations.**

Four types of functional terms significantly and differentially enriched between RIO and BTx406/R9188, including GO biological process (GOBP) **(a)**, GO molecular function (GOMF) **(b)**, KEGG pathways **(c)** and Plant Metabolic Network (PMN) pathways **(d)**, are shown in heatmap. The functional terms were sorted by the genotypes, the expression trends and modules in which they are enriched. The modules were grouped first according to genotypes and then expression tendencies. Significance of enrichment was defined as *P* < 0.05 and *q* value < 0.2 (see Appendix S1). The differentially enriched functions are categorized into three groups (central metabolism, transport and regulation and signaling) and are highlighted in blue, dark red and orange, respectively. The distributions of *P* values and *q* values of the enriched GOBP, GOMF, KEGG pathways and PMN pathways are shown in histogram.


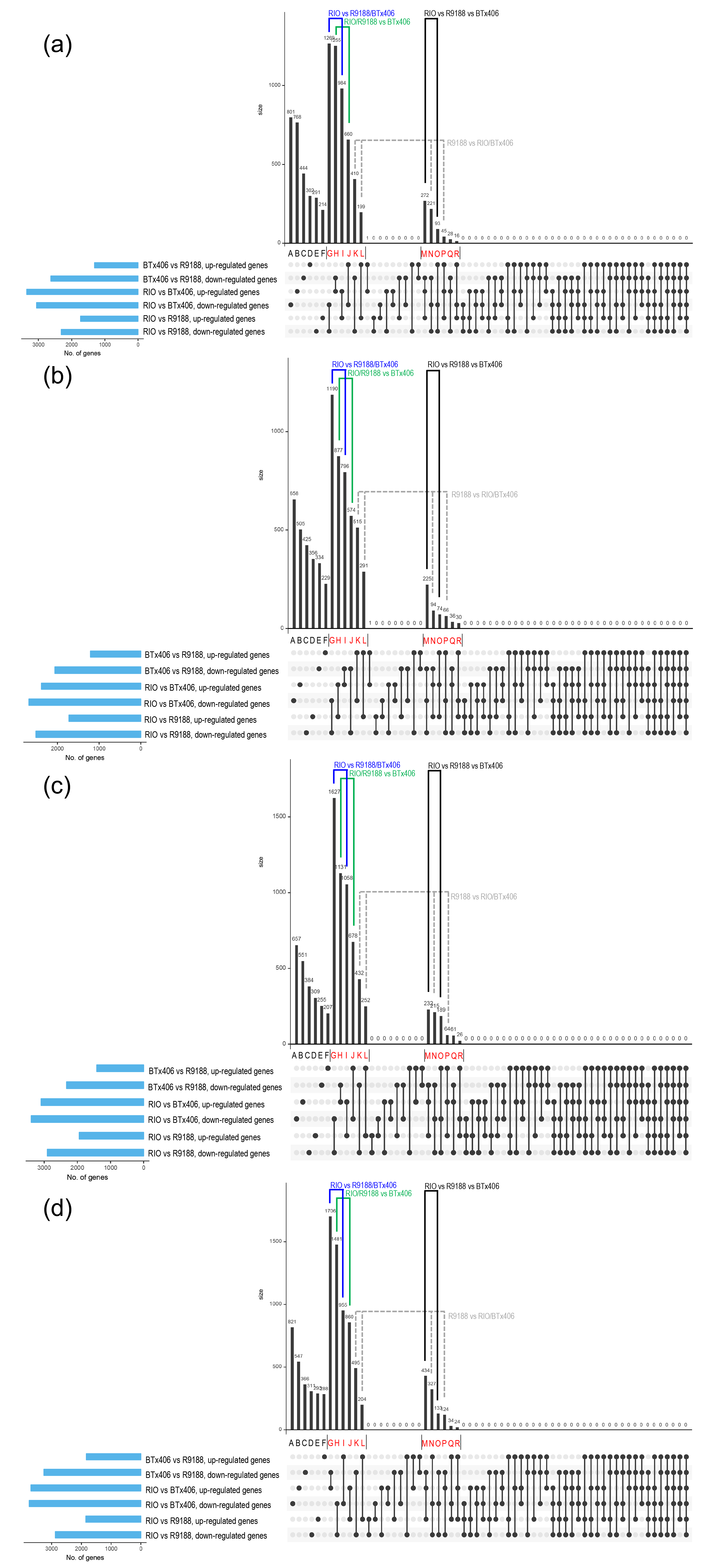


**Figure S11** Identification of DEGs representing the differences of RIO vs R9188/BTx406, RIO/R9188 vs BTx406 and RIO vs R9188 vs BTx406 by using upset plot visualization. Upset plot visualization of the intersections between up- and down-regulated gene sets for the comparison groups: RIO vs BTx406, RIO vs R9188 and R9188 vs BTx406 at T1 (a), T2 (b), T3 (c) and T4 (d) time points.


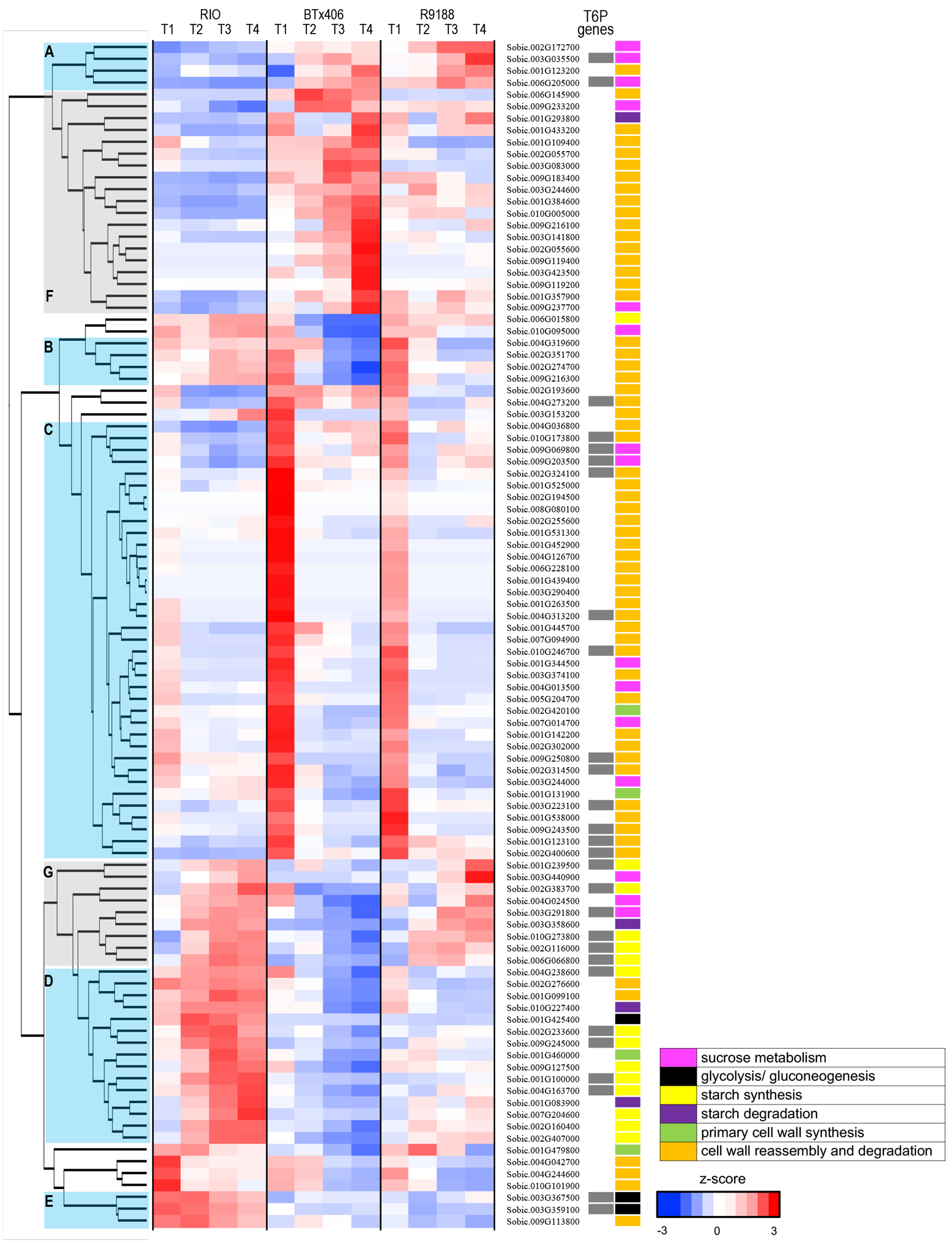


**Figure S12** Hierarchical clustering of the genes associated with enriched major CHO functional terms by using the second analysis approach. Gene expression (RPKM) levels were transformed to z-score across genotypes and time points and colored blue, white and red to represent low, moderate and high expression, respectively. Based on the clustering results and gene expression patterns, seven clusters of genes were identified. Cluster A: up-regulated in R9188/BTx406 but low expressed in RIO; Cluster B: down-regulated in R9188/BTx406 but unchanged in RIO; Cluster C: down-regulated in R9188/BTx406 but slightly down-regulated in RIO; Cluster D: up-regulated in RIO but not in R9188/BTx406; Cluster E: highly-expressed in RIO but low expressed in R9188/BTx406; Cluster F: up-regulated only in BTx406, reflecting the transcriptomic difference of RIO vs R9188 vs BTx406; Cluster G: up-regulated in RIO/R9188 but low expressed in BTx406, reflecting the transcriptomic difference of RIO/R9188 vs BTx406. Clusters A-E reflect the transcriptomic differences of RIO vs R9188/btX406. T6P-regulated genes are labeled in the “T6P genes” column. The gene function is annotated as in Data S4and S5 and color-coded.


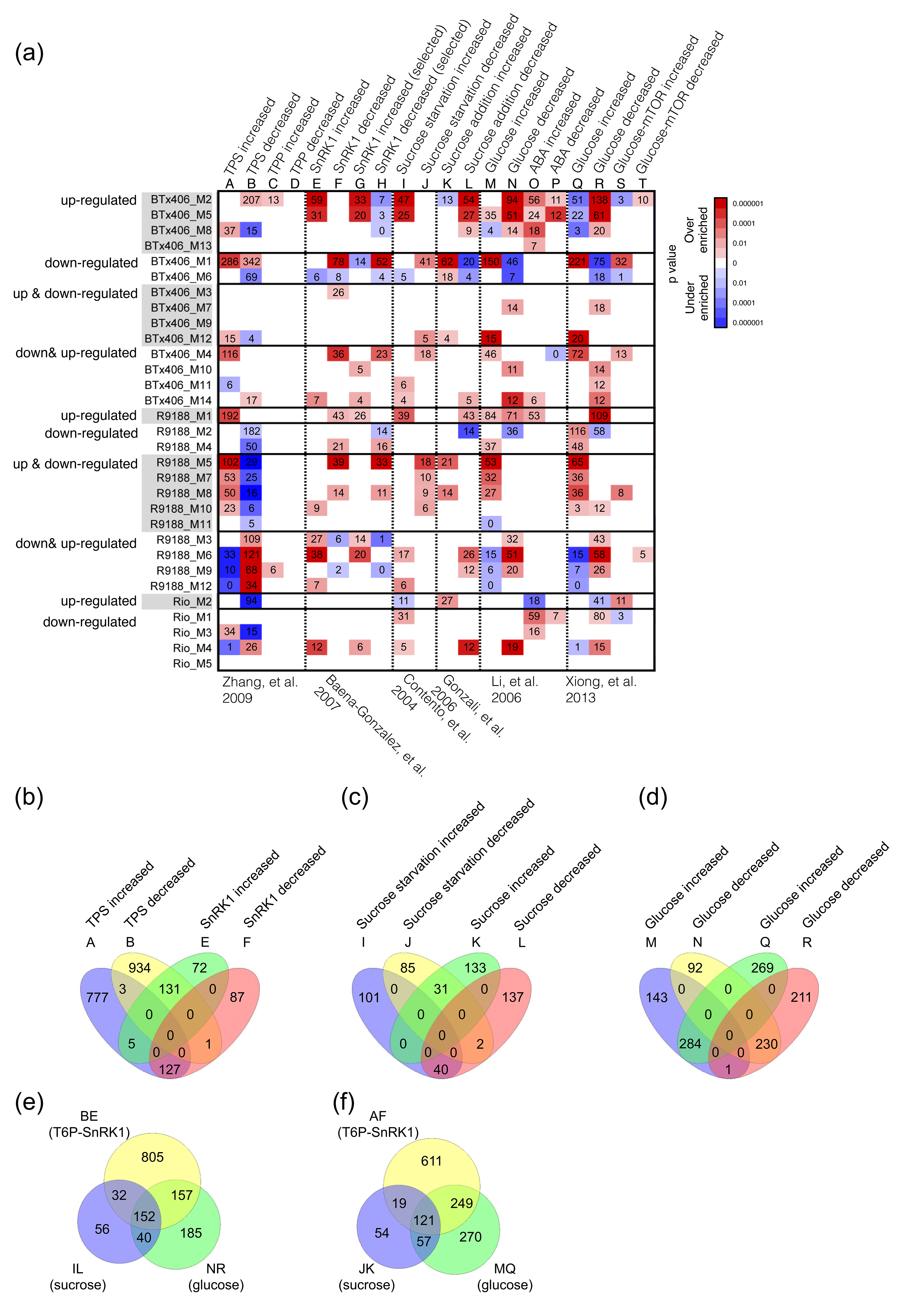


**Figure S13 Pair-wise comparison of the sorghum co-expression modules with public available gene sets responsive/regulated by sugar signaling in Arabidopsis.**

**(a)** Heatmap plot showed that several modules in sorghum were significantly overlapped with gene sets inducible/ repressible by T6P signaling, SnRK1, sucrose and glucose in Arabidopsis. Significant over-enrichment between gene sets was indicated by red, while significant under-enrichment was indicated by blue. The *p* value was shown by the shade of colors. The number of overlapped genes was shown in heatmap.

**(b-f)** To simplify the overlapping patterns which could be due to the redundancy between *Arabidopsis* gene sets, the T6P- and SnRK1- related gene sets **(b)**, and sucrose starvation and sucrose regulated gene sets **(c)**, as well as two glucose gene sets **(d)** were compared, respectively.

To further remove redundancy and generate non-redundant *Arabidopsis* gene sets which may be responsive to or regulated by T6P-SnRK1, sucrose and glucose, the combined gene sets were analyzed by Venn diagram, yielding 6 non-redundant gene sets (**e** and **f**). These six non-redundant gene sets represented T6P-SnRK1 inducible / repressible gene sets, sucrose inducible/ repressible gene sets (other than through the T6P-SnRK1 pathway), and glucose inducible /repressible gene sets.


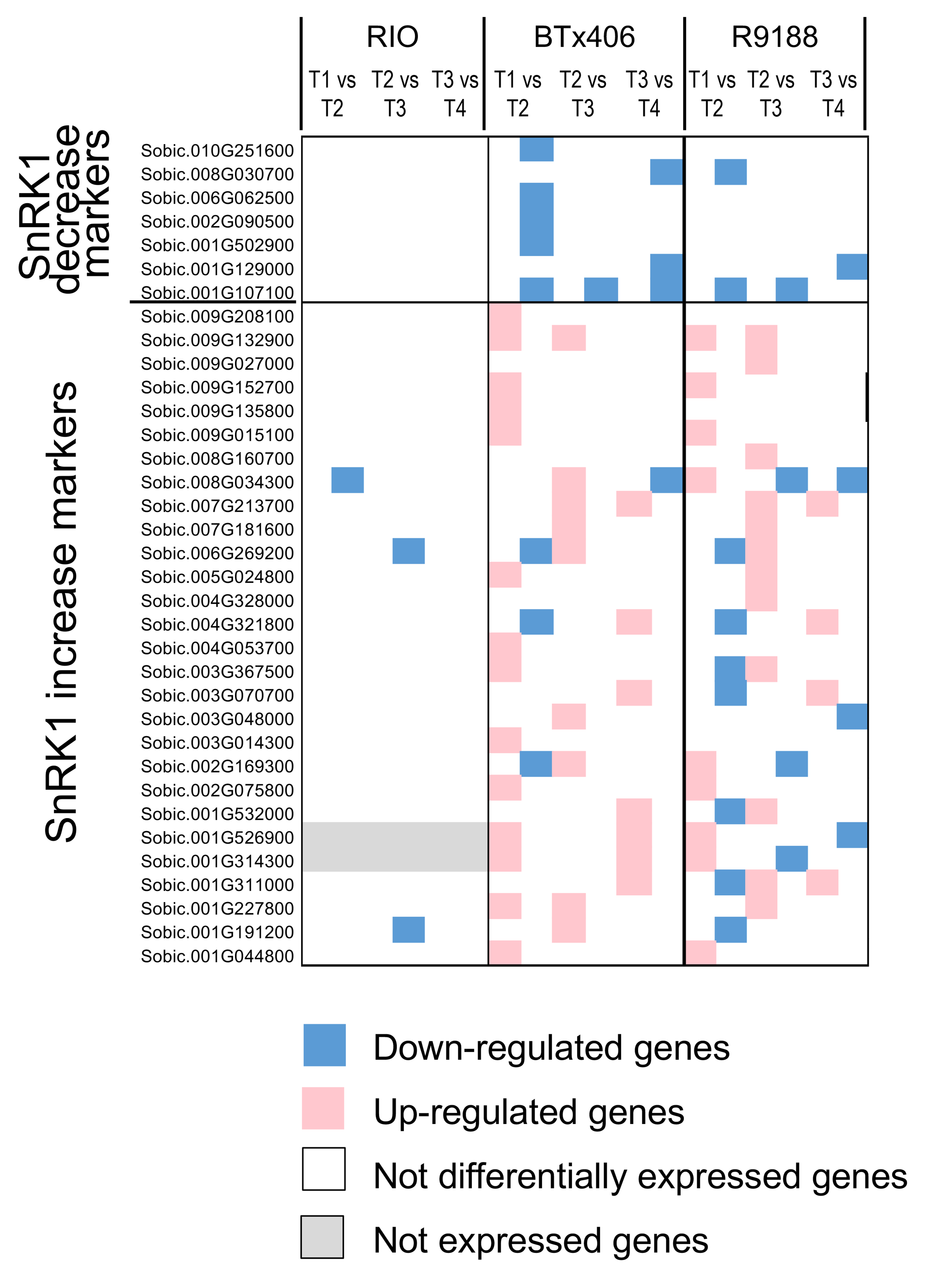


**Figure S14** SnRK1 marker gene expression previously identified in Oszvald et al. (2018); Baena-Gonzalez et al. (2007); Zhang et al. (2009); Martinez-Barajas et al. (2011) in RIO compared to BTx406 and R9188.


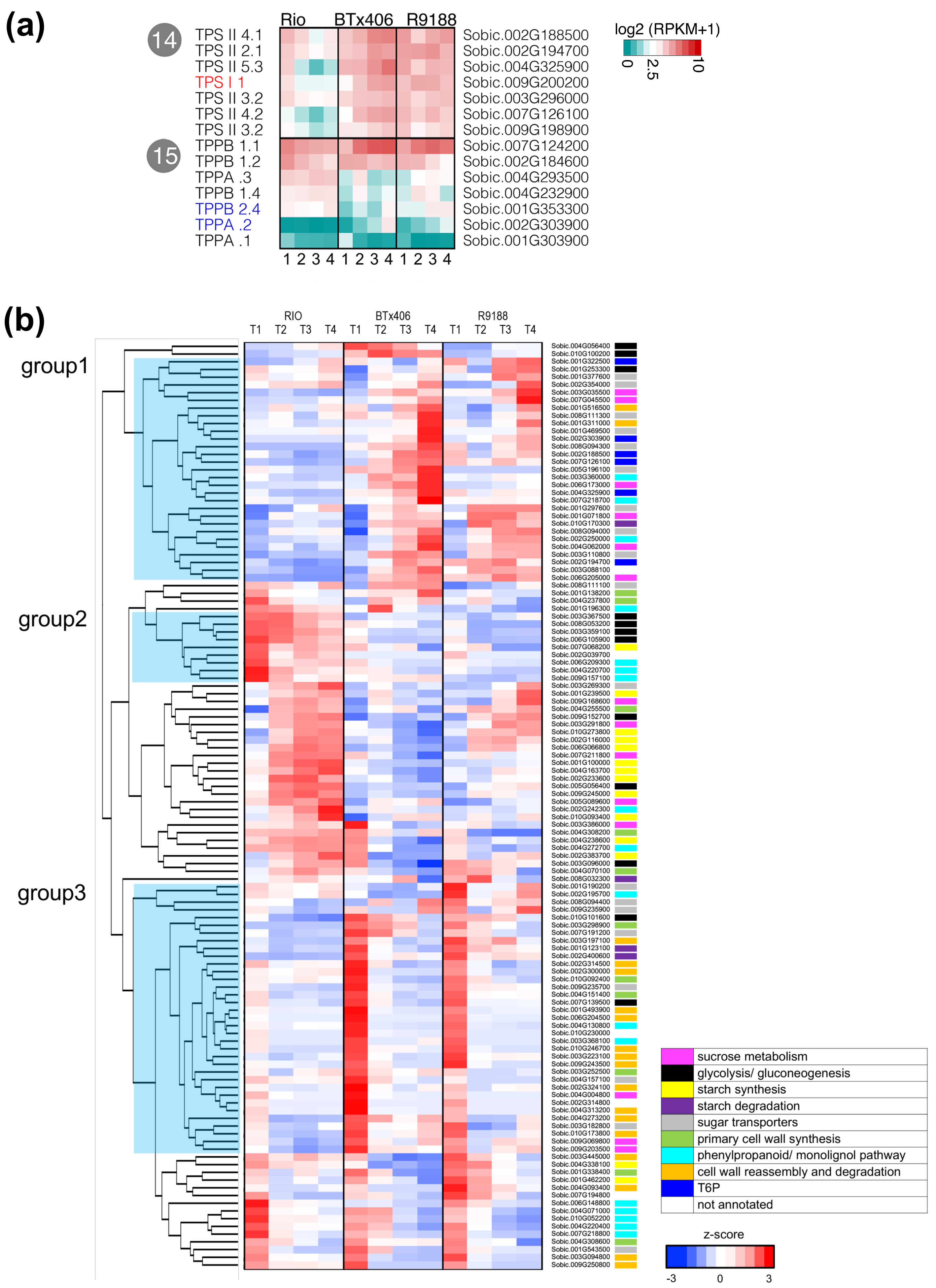


**Figure S15 (a)** Heat map of the DEGs in trehalose biosynthetic pathway. **(b)** Hierarchical clustering of T6P-regulated genes which are associated with primary metabolism and sugar transport. Gene expression (RPKM) levels were transformed to z-score across genotypes and time points and colored blue, white and red to represent low, moderate and high expression, respectively. Hierarchical clustering identified three clusters of genes representing the transcriptomic difference of RIO vs R9188/BTx406. Group 1: genes up-regulated in R9188/BTx406 but unchanged and low expressed in RIO; Group 2: genes down-regulated and highly expressed in RIO but low expressed in R9188/BTx406; Group 3: genes down-regulated in R9188/BTx406 but not in RIO. The functions of genes are annotated as in Data S4 and S5 and color-coded.


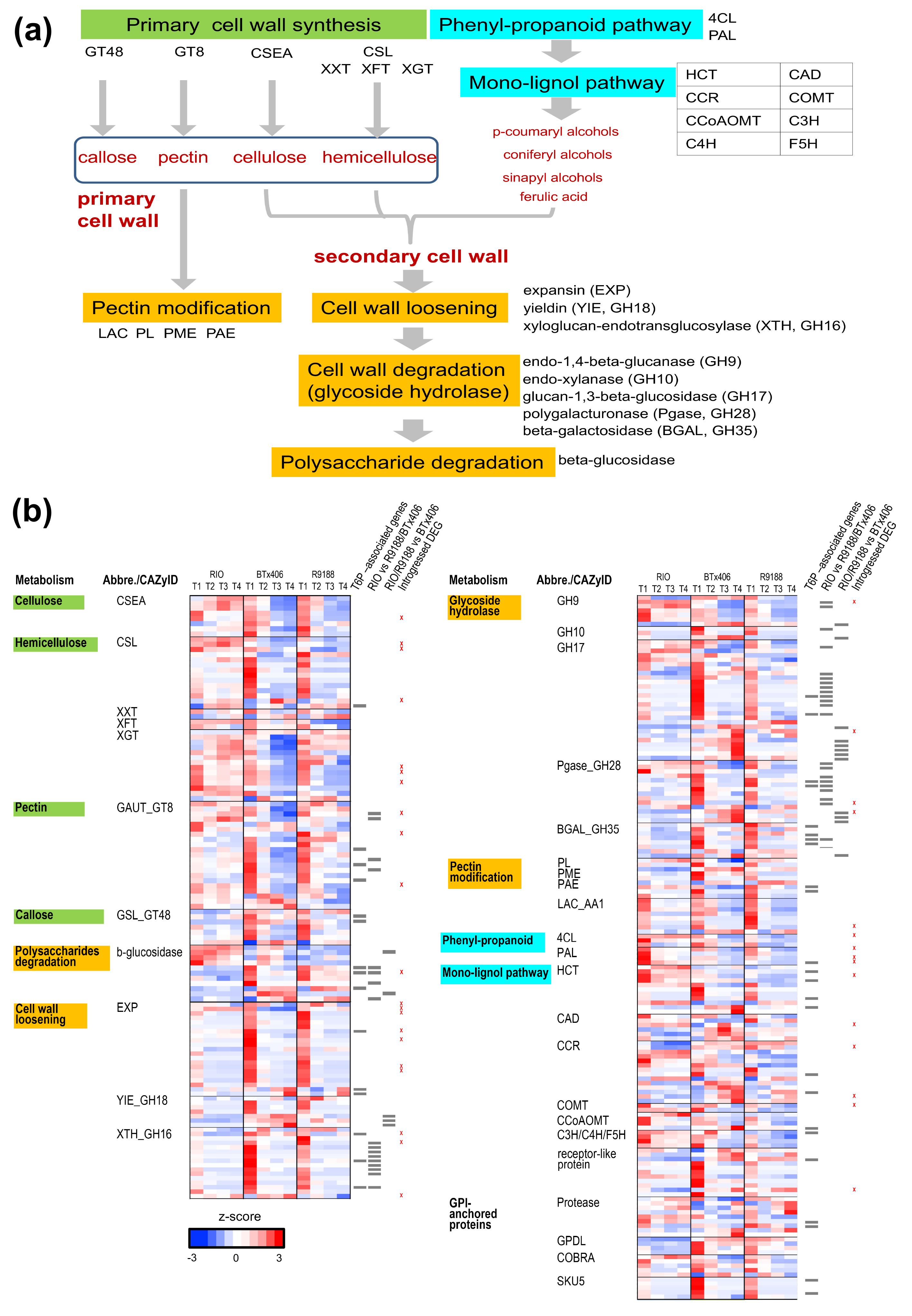


**Figure S16 Expression profiles of cell wall related genes in sorghum.**

**(a)** Diagram summarizing the genes related to cell wall metabolism. Gene families responsible for primary cell wall synthesis are labeled in green. Gene families responsible for lignin synthesis (including phenyl-propanoid synthesis and mono-lignol synthesis pathways) are labled in light blue. Gene families labeled in yellow are responsible for cell wall reassembly and degradation, including pectin modification, cell wall loosening, glycoside hydrolase and polysaccharide degradation. Compounds involved in cell wall metabolism are highlighted in red.

**(b)** Expression patterns of cell wall metabolic genes between RIO, R9188 and BTx406. Gene expression (RPKM) levels were transformed to z-score across genotypes and time points and colored blue, white and red to represent low, moderate and high expression, respectively. Cell wall metabolic genes are ordered first by gene family and second by hierarchical clustering. T6P-regulated genes are labeled. Genes located in the introgression regions of R9188 are highlighted with red “X”. Genes associated with differences of RIO vs R9188/BTx406 and RIO/R9188 vs BTx406 were determined according to Figure S12. Only genes differentially expressed in at least one genotype were shown in the heatmap.

**Enzyme abbreviations:** CesA, cellulose synthase; CSL, cellulose synthase-like; XXT, xyloglucan xylosyl transferases and galactomannan galtransferases; XFT, xyloglucan fucosyltransferases; XGT, xyloglucan galactosyltransferases; GAUT, homogalacturonan α-1,4- galacturonosyltransferases; GSL, glucan synthase-like (callose synthases); EXP, expansins; YIE, yieldins; XTH, xyloglucan endotransglucosylases /hydrolases; PL1, pectate and pectin lyases; PL4, rhamnogalacturonan I lyases; PME, pectin methyl esterases; PAE, pectin acetyl esterases; LAC, laccases; GH9, endo- 1,4-β-glucanases; GH10, endo-xylanases; GH17, glucan1,3- β -glucosidases; Pgase, polygalacturonases; BGAL, β -Galactosidases.


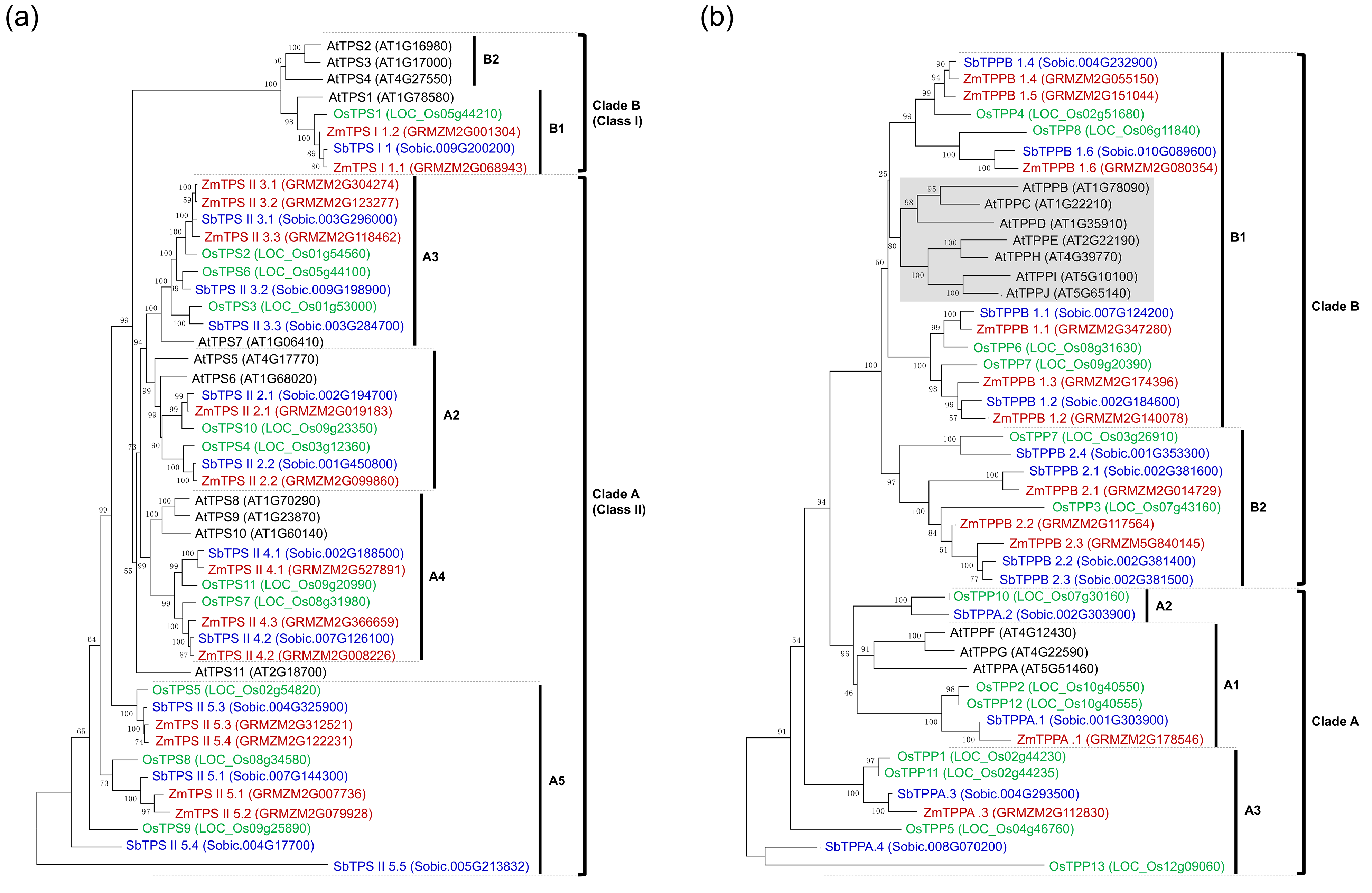


**Figure S17 Phylogenetic analysis of TPS (A) and TPP (B) genes from sorghum (blue), maize (red), rice (green) and *Arabidopsis* (black).**

Clades and subclades were indicated and an *Arabidopsis*-specific group of TPP was shaded in grey.


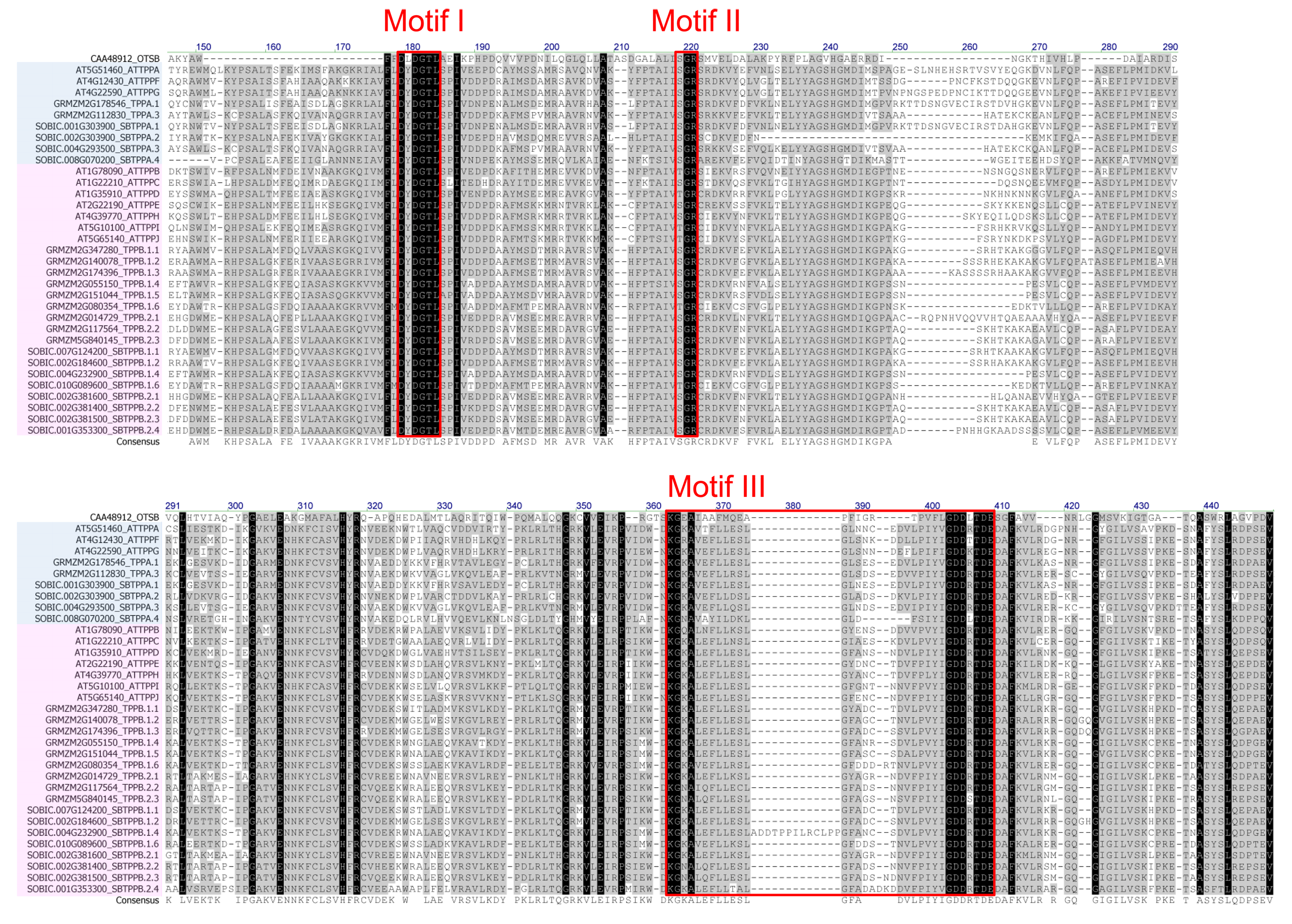


**Figure S18 Multiple sequence alignment for the TPP gene family revealed high conservation in the TPP domain.**

TPP sequences from *E.coli*, *Arabidopsis*, rice, maize and sorghum were used. Only the sequences of TPP domain are shown. The red box indicated the three conserved motifs necessary for TPP enzyme function. Unlike TPP-like domains in TPS genes, TPP genes showed strikingly high conservation in their TPP domains.


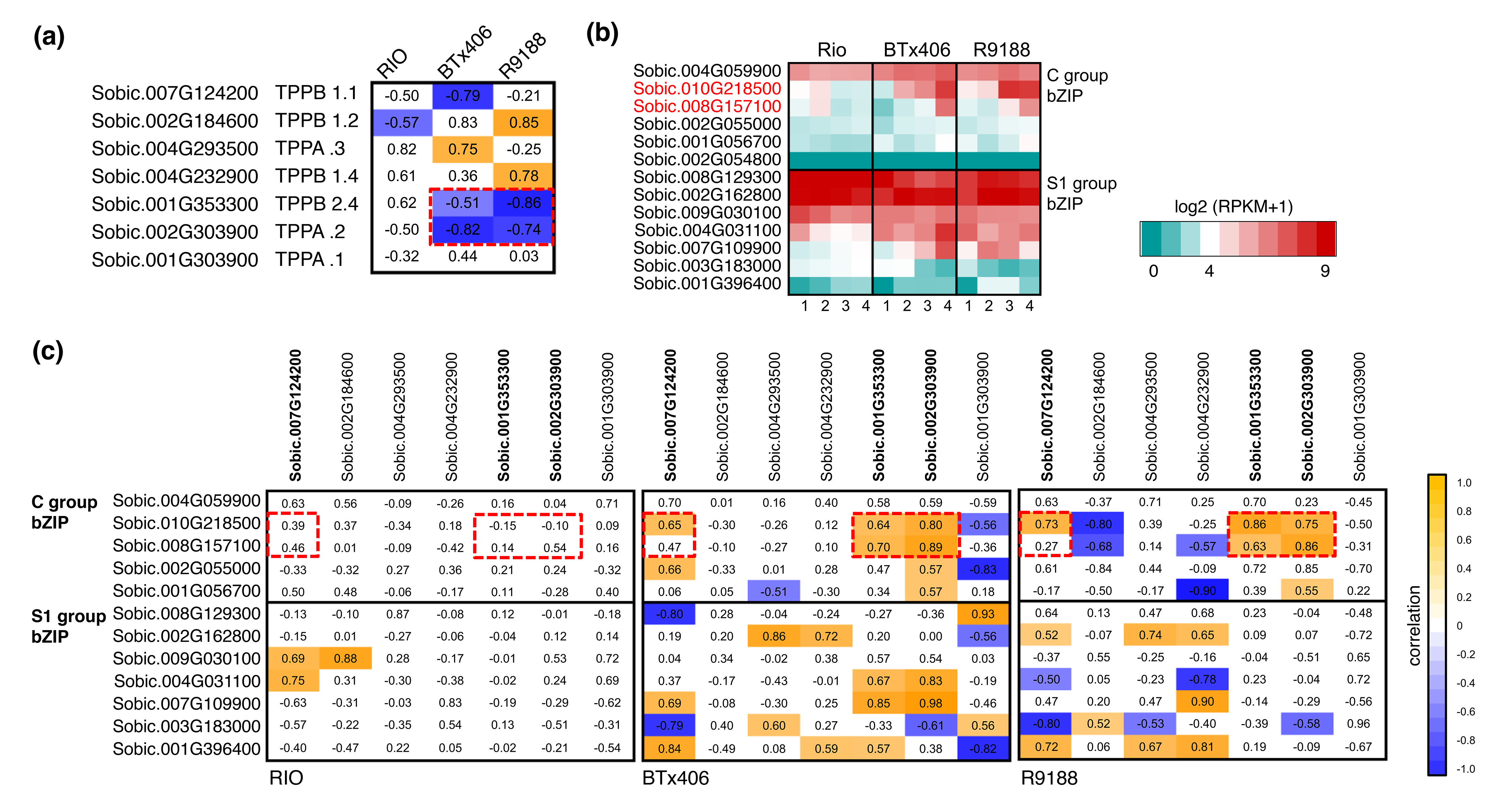


**Figure S19 Expression profiles of the C/S1 groups of bZIP and correlation analysis of the TPP family and C/S1 groups of bZIP in sorghum.**

**(a)** Correlation analysis between sorghum *TPP* genes and T6P in RIO, BTx406 and R9188 identified that two *TPP* genes were correlated with T6P abundances in BTx406 and R9188, and also showed differential expression between RIO and BTx406/R9188 (Figure1; Supplementary Table 5). Given the differentially expression patterns of the TPPB 1.1 (Sobic.007G124200; Figure 1) and its high expression levels, this gene was included in the following correlation analysis even through it didn’t show strong correlation with T6P in R9188.

**(b)** Expression patterns of C/S1 groups of bZIP in sorghum stems.

**(c)** Correlation analysis between the C/S1 *bZIP* and *TPP* genes identified that two C group *bZIP*s (Sobic.010G218500 and Sobic.008G157100) were correlated with T6P-associated *TPP* genes and showed differential regulation patterns between RIO and BTx406/R9188. In particular, Sobic.008G157100 was introgressed from BTx406 into R9188. These two bZIPs were highlighted in red. Three *TPP* genes potentially regulating T6P are labeled in bold.

Pearson correlation coefficiency (*r*) were calculated and shown in heatmap. If a gene was not detected as a DEG, its correlations with other genes or T6P were not considered and are not shown by the color scale in heat maps. Only correlations from DEGs with a minimum |*r*| value of 0.5 were shown.


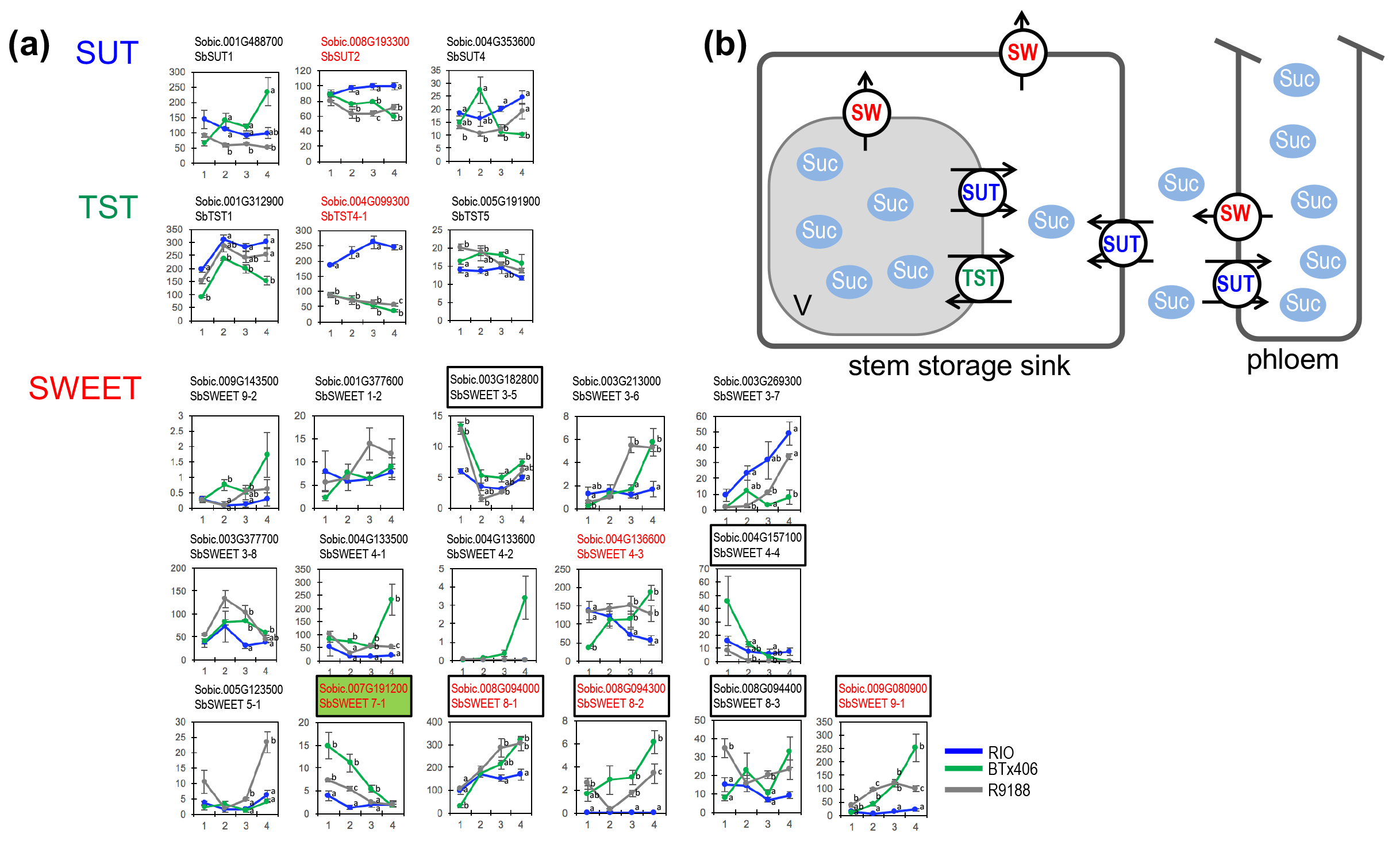


**Figure S20 Expression profiles of sugar transporter genes during sugar accumulation (a) and a proposition of a hypothetic model illustrates the roles of different sugar transporters in sorghum internode (b).**

**(a)** The expression levels of three gene families encoding sugar transporters, only DEGs were shown. Sucrose Transporters (SUT), Tonoplast Sugar Transporters (TST), Sugars Will Eventually be Exported Transporters (SWEET). For each gene, the expression differences between genotypes at the same time points were tested using ANOVA followed by *post hoc* Tukey’s test (*p*<0.05) and statistical differences are presented using letter display. The SbSWEET7-1 was introgressed from BTx406 into R9188, highlighted in green background. Clade III SWEETs in sorghum were shown in black box according to a previous study (Mizuno et al. 2016). Based on the expression dynamics between the genotypes, phylogenetic relationship and properties of transporters, the genes that could play roles in sugar accumulation were highlighted in red. The only SWEET gene introgressed from BTx406 into R9188 is highlighted in green background. The nomenclature of SUT, SWEET and TMT was according to previous studies (Bihmidine et al., 2015, 2016; Mizuno et al., 2016).

(b) In sorghum internode, sucrose may possibly be released from phloem from SWEET (SbSWEET4-3 or SbSWEET8-1) to the apoplasm and SbSUTs may play roles in uptake sucrose into stem storage parenchyma cells (sink) and/or retrieving sucrose leaked from phloem. Based on the association of expression levels and their sucrose concentration between genotypes, it is hypothesized that SbSUT2 may be the transporter for uptaking sucrose into stem storage parenchyma cells. SbTSTs (possibly SbTST4-1) may play roles in uptaking sucrose into storage vacuole. SWEET transporters (likely SbSWEET7-1, 8-1, 8-2 and 9-1) may also play roles in release sucrose into cytoplasm to maintain cytosolic sucrose levels for cell metabolism. SWEET is abbreviated as “SW”. V: vacuole; Suc: sucrose.

**Table S1. Sorghum trait descriptions.**

| Trait | Trait names/  synonyms | Trait descriptions | Ontology source | Term ID |
| --- | --- | --- | --- | --- |
| Brix | Stem juice sugar content | Sugar content in sorghum stem juice | Crop ontology | CO_324:0000196 |
| Brix | total water soluble content | A metabolite content related trait (TO:0000281) which is the concentration of water soluble components in a plant, as determined by refractive index. | Plant ontology | TO:0020088 |
| sucrose concentration | sucrose content | The amount of sucrose present in the plant or the plant part. | Plant ontology | TO:0000328 |
| glucose concentration | glucose content | Amount of glucose present in the plant or plant part. | Plant ontology | TO:0000300 |
| fructose concentration | fructose content | Amount of fructose present in the plant or plant part. | Plant ontology | TO:0006005 |

**Table S2. Quality control (QC) precision values of the 14 compounds analyzed by targeted metabolic profiling.**

The criteria for targeted metabolic profiling is coefficient of variance (%CV) ≤ 20% for intra- and inter- run QC precision. The increased precision values of galactinol and stachyose are due to lack of labeled internal standards.

| Metabolite | Intra-run CV (%) | Maximum of intra-run CV | Minimum of intra-run CV | Inter-run CV (%) |
| --- | --- | --- | --- | --- |
| Adenosine | 1.9 ± 0.7 | 2.9 | 1.3 | 2.7 |
| Glucose 1-phosphate | 6.3 ± 1.6 | 8.1 | 4.7 | 7.4 |
| Glucose 6-phosphate | 4.8 ± 1.2 | 6.3 | 3.3 | 5.4 |
| Sucrose 6-phosphate | 5.1 ± 2.4 | 7.2 | 1.6 | 6.5 |
| Trehalose 6-phosphate | 2.8 ± 0.9 | 3.9 | 1.8 | 3.7 |
| UDPG | 3.1 ± 1.4 | 5.2 | 2.0 | 4.4 |
| Galactinol | 12.1 ± 9.7 | 24.5 | 2.9 | 15.5 |
| Inositol | 10.7 ± 4.3 | 15.5 | 5.9 | 18.6 |
| Sorbitol | 2.6 ± 2.2 | 5.7 | 0.9 | 4.7 |
| Stachyose | 9.0 ± 4.0 | 14.1 | 4.7 | 26.3 |
| Trehalose | 5.8 ± 2.0 | 8.6 | 4.2 | 6.2 |
| Fructose | 4.7 ± 2.0 | 6.7 | 2.9 | 4.7 |
| Glucose | 3.0 ± 0.9 | 4.3 | 2.3 | 4.4 |
| Sucrose | 2.4 ± 0.2 | 2.6 | 2.2 | 2.5 |

**Table S3. Pairwise correlations calculated from any two of the six replicates for metabolome analysis.** (Data shown as mean ± standard deviation)

| Genotype | Time point | Correlation |
| --- | --- | --- |
| BTx406 | T1 | 0.86 ± 0.03 |
|  | T2 | 0.75 ± 0.05 |
|  | T3 | 0.75 ± 0.06 |
|  | T4 | 0.86 ± 0.05 |
|  | T5 | 0.90 ± 0.03 |
| R9188 | T1 | 0.81 ± 0.05 |
|  | T2 | 0.76 ± 0.06 |
|  | T3 | 0.81 ± 0.05 |
|  | T4 | 0.82 ± 0.05 |
|  | T5 | 0.88 ± 0.03 |
| RIO | T1 | 0.82 ± 0.04 |
|  | T2 | 0.84 ± 0.06 |
|  | T3 | 0.86 ± 0.04 |
|  | T4 | 0.82 ± 0.07 |
|  | T5 | 0.88 ± 0.02 |

**Table S4. Summary of RNA-seq mapping results.**

| Genotype | Time point | Replicate | Total raw reads | Mapped reads | % Mapped reads | Unique mapped reads | % unique mapped reads | SRA accession |
| --- | --- | --- | --- | --- | --- | --- | --- | --- |
| Rio | 1 | Rep1 | 54,874,288 | 48,368,027 | 88.1 | 13,840,650 | 28.6 | SRR6188852 |
| Rio | 1 | Rep2 | 50,993,248 | 44,729,442 | 87.7 | 17,562,142 | 39.3 | SRR6188853 |
| Rio | 1 | Rep3 | 50,636,744 | 44,393,475 | 87.7 | 24,912,316 | 56.1 | SRR6188854 |
| Rio | 2 | Rep1 | 45,987,314 | 32,627,854 | 70.9 | 12,753,900 | 39.1 | SRR6188855 |
| Rio | 2 | Rep2 | 70,966,026 | 62,615,629 | 88.2 | 23,576,118 | 37.7 | SRR6188848 |
| Rio | 2 | Rep3 | 59,314,178 | 52,912,536 | 89.2 | 21,112,812 | 39.9 | SRR6188849 |
| Rio | 3 | Rep1 | 65,847,632 | 58,260,562 | 88.5 | 22,789,914 | 39.1 | SRR6188850 |
| Rio | 3 | Rep2 | 60,737,226 | 52,447,763 | 86.4 | 21,165,426 | 40.4 | SRR6188851 |
| Rio | 3 | Rep3 | 54,800,364 | 49,328,350 | 90.0 | 19,313,216 | 39.2 | SRR6188846 |
| Rio | 4 | Rep1 | 61,274,984 | 53,296,877 | 87.0 | 25,730,360 | 48.3 | SRR6188847 |
| Rio | 4 | Rep2 | 55,142,514 | 49,546,979 | 89.9 | 21,193,878 | 42.8 | SRR6188828 |
| Rio | 4 | Rep3 | 66,500,078 | 59,094,784 | 88.9 | 22,201,454 | 37.6 | SRR6188829 |
| BTx406 | 1 | Rep1 | 60,459,958 | 50,060,333 | 82.8 | 41,752,720 | 83.4 | SRR6188830 |
| BTx406 | 1 | Rep2 | 53,408,128 | 49,429,773 | 92.6 | 19,468,878 | 39.4 | SRR6188831 |
| BTx406 | 1 | Rep3 | 44,729,116 | 39,979,143 | 89.4 | 18,368,606 | 45.9 | SRR6188832 |
| BTx406 | 2 | Rep1 | 53,178,908 | 48,920,241 | 92.0 | 26,491,158 | 54.2 | SRR6188833 |
| BTx406 | 2 | Rep2 | 37,638,226 | 33,985,425 | 90.3 | 15,901,070 | 46.8 | SRR6188834 |
| BTx406 | 2 | Rep3 | 43,236,530 | 39,114,039 | 90.5 | 18,356,078 | 46.9 | SRR6188835 |
| BTx406 | 3 | Rep1 | 59,887,648 | 55,468,329 | 92.6 | 30,767,288 | 55.5 | SRR6188820 |
| BTx406 | 3 | Rep2 | 23,365,530 | 20,136,494 | 86.2 | 11,436,426 | 56.8 | SRR6188821 |
| BTx406 | 3 | Rep3 | 55,139,178 | 50,180,813 | 91.0 | 25,496,606 | 50.8 | SRR6188841 |
| BTx406 | 4 | Rep1 | 46,401,418 | 40,031,525 | 86.3 | 12,649,848 | 31.6 | SRR6188840 |
| BTx406 | 4 | Rep2 | 44,615,056 | 38,630,919 | 86.6 | 19,741,324 | 51.1 | SRR6188843 |
| BTx406 | 4 | Rep3 | 50,048,850 | 44,532,775 | 89.0 | 23,138,282 | 52.0 | SRR6188842 |
| R9188 | 1 | Rep1 | 53,101,964 | 40,055,433 | 75.4 | 33,478,490 | 83.6 | SRR6188837 |
| R9188 | 1 | Rep2 | 49,758,666 | 45,358,736 | 91.2 | 41,658,560 | 91.8 | SRR6188836 |
| R9188 | 1 | Rep3 | 65,540,334 | 58,725,714 | 89.6 | 52,532,870 | 89.5 | SRR6188839 |
| R9188 | 2 | Rep1 | 57,338,700 | 49,365,887 | 86.1 | 44,479,334 | 90.1 | SRR6188838 |
| R9188 | 2 | Rep2 | 33,497,424 | 26,146,766 | 78.1 | 22,936,514 | 87.7 | SRR6188845 |
| R9188 | 2 | Rep3 | 63,734,518 | 54,621,499 | 85.7 | 46,729,570 | 85.6 | SRR6188844 |
| R9188 | 3 | Rep1 | 47,545,346 | 42,839,571 | 90.1 | 39,558,666 | 92.3 | SRR6188826 |
| R9188 | 3 | Rep2 | 54,986,642 | 49,997,401 | 90.9 | 46,743,168 | 93.5 | SRR6188827 |
| R9188 | 3 | Rep3 | 59,254,582 | 51,269,787 | 86.5 | 44,681,632 | 87.2 | SRR6188824 |
| R9188 | 4 | Rep1 | 47,926,820 | 41,960,991 | 87.6 | 37,068,888 | 88.3 | SRR6188825 |
| R9188 | 4 | Rep2 | 51,189,792 | 45,744,413 | 89.4 | 40,880,948 | 89.4 | SRR6188822 |
| R9188 | 4 | Rep3 | 48,167,306 | 41,757,531 | 86.7 | 38,820,194 | 93.0 | SRR6188823 |

**Table S5. Pairwise correlations of bioreplicates used in the RNA-seq analysis**

| Genotype | Time point | R1 vs R2 | R1 vs R3 | R2 vs R3 |
| --- | --- | --- | --- | --- |
| Rio | T1 | 0.927 | 0.920 | 0.964 |
|  | T2 | 0.969 | 0.970 | 0.971 |
|  | T3 | 0.978 | 0.978 | 0.986 |
|  | T4 | 0.981 | 0.971 | 0.983 |
| BTx406 | T1 | 0.963 | 0.969 | 0.985 |
|  | T2 | 0.949 | 0.989 | 0.955 |
|  | T3 | 0.977 | 0.983 | 0.978 |
|  | T4 | 0.969 | 0.974 | 0.966 |
| R9188 | T1 | 0.972 | 0.977 | 0.989 |
|  | T2 | 0.979 | 0.967 | 0.969 |
|  | T3 | 0.982 | 0.982 | 0.982 |
|  | T4 | 0.982 | 0.973 | 0.977 |

**Table S6. Numbers of expressed genes detected in each genotype and time point.**

| Genotype | No. of expressed genes in T1 | No. of expressed genes in T2 | No. of expressed genes in T3 | No. of expressed genes in T4 | No. of expressed genes in any of the four time points |
| --- | --- | --- | --- | --- | --- |
| RIO | 17,417 | 16,988 | 16,981 | 17,155 | 18,275 |
| BTx406 | 18,028 | 17,223 | 16,984 | 17,009 | 19,727 |
| R9188 | 17,724 | 16,931 | 16,748 | 16,626 | 19,102 |
| total | nd | nd | nd | nd | 21,113 |

nd: not determined.

**Table S7. Number of overlapping genes between introgressed DEGs and R9188 DEGs which might potentially be regulated by the T6P/SnRK1 signaling network.**

| Expression tendency | Modules | T6P-SnRK1 regulated genes | No. of overlapped genes with DEGs from introgression regions |
| --- | --- | --- | --- |
| Up | R9188_M1 | T6P-SnRK1 inducible | 34 |
|  |  | T6P-SnRK1 repressible | 37 |
| Down | R9188_M2 | T6P-SnRK1 inducible | 29 |
|  |  | T6P-SnRK1 repressible | 37 |
|  | R9188_M4 | T6P-SnRK1 inducible | 9 |
|  |  | T6P-SnRK1 repressible | 8 |
| Up & down | R9188_M5 | T6P-SnRK1 inducible | 17 |
|  |  | T6P-SnRK1 repressible | 2 |
|  | R9188_M7 | T6P-SnRK1 inducible | 5 |
|  |  | T6P-SnRK1 repressible | 5 |
|  | R9188_M8 | T6P-SnRK1 inducible | 7 |
|  |  | T6P-SnRK1 repressible | 4 |
| Down & up | R9188_M3 | T6P-SnRK1 inducible | 9 |
|  |  | T6P-SnRK1 repressible | 22 |
|  | R9188_M6 | T6P-SnRK1 inducible | 6 |
|  |  | T6P-SnRK1 repressible | 18 |
|  | R9188_M9 | T6P-SnRK1 inducible | 5 |
|  |  | T6P-SnRK1 repressible | 10 |
|  | R9188_M12 | T6P-SnRK1 inducible | 0 |
|  |  | T6P-SnRK1 repressible | 7 |

**Table S8. Distribution of the SNP effects predicted from BTx406 SNPs and RIO SNPs.**

| **Putative SNP impact** | **Variant type** | **SNPs from BTx406** | **SNPs from RIO** |
| --- | --- | --- | --- |
| **High** | **Total** | **19** | **35** |
|  | Start_lost | 1 | 1 |
|  | Stop_gained | 11 | 22 |
|  | Stop_lost | 0 | 4 |
|  | Stop_lost & splice_region_variant | 1 | 0 |
|  | Splice_donor_variant & intron_variant | 2 | 5 |
|  | Splice_acceptor_variant & intron_variant | 4 | 3 |
| **Moderate** | **Total** | **2596** | **2607** |
|  | Missense_variant & splice_region_variant | 26 | 25 |
|  | Missense_variant | 2570 | 2582 |
| **Low** | **Total** | **3961** | **4001** |
|  | Stop_retained_variant | 6 | 3 |
|  | Synonymous_variant | 3730 | 3726 |
|  | Splice_region_variant & Stop_retained_variant | 0 | 1 |
|  | Splice_region_variant & Synonymous_variant | 43 | 65 |
|  | Splice_region_variant & intron_variant | 47 | 56 |
|  | Splice_region_variant | 33 | 29 |
|  | Initiator_codon_variant | 2 | 0 |
|  | 5’ UTR_premature_start_codon_gained | 100 | 121 |
| **Modifier** | **Total** | **7037** | **7012** |
|  | Intron_variant | 3164 | 3069 |
|  | 5’UTR_variant | 852 | 907 |
|  | 3’UTR_variant | 2860 | 2800 |
|  | Intergenic_region | 161 | 236 |

**Appendix S1.**

**1. Field experiments**

Three sorghum genotypes, BTx406, RIO and R9188, were used. BTx406 is early-flowering and dwarf, whereas RIO is late-flowering and tall with high contents of soluble sugars accumulating in its stem. R9188 is a dwarf converted inbred line developed in Texas (Ritter et al. 2004). It was selected from the BTx406/RIO cross followed by one backcross to RIO (Ritter 2007). The early maturing and dwarf loci were introgressed from BTx406 into R9188. R9188 (accession number PI 656007) was obtained from the United States Department of Agriculture National Plant Germplasm System (USDA-NPGS) and maintained in the lab by self-pollination since 2010.

Field experiments were conducted in the experimental field of the Waksman Institute, Rutgers, The State University of New Jersey in 2014, using a split plot design with three replicates. Each block consisted of three plots, with each sorghum genotype randomly planted in one plot. Each plot consisted of five rows which had 25 plants per row. The sorghum seeds of RIO, BTx406 and R9188 were planted on June 17^th^ and 18^th^ 2014. BTx406 and R9188 had similar flowering date (69 days and 73 days on average, respectively), while RIO flowered late (95 days). To obtain the dynamics of soluble sugars in sorghum stem, five time points were chosen to collect stem tissues for detailed analysis: flag leaf stage (T1), 100% flowering (T2), 10 days after flowering (T3), 15 days after flowering (T4) and 30 days after flowering (T5). In each plot, the three central rows were further divided into five subplots containing nine plants per subplot, with each subplot (nine plants) randomly corresponding to one harvest time point (Figure S1). To obtain tissues for Brix measurement, metabolome, and RNA-seq, the upper internodes (internode No. 2, 3 and 4) were harvested from the nine plants per subplot at each of the five time points. Internodes were numbered from top to bottom. For measuring brix, three central plants per subplot were harvested and two ~1 cm-long sections of internodes were cut, with one section stored on ice for stem juice extraction and brix measurement and another immediately frozen in liquid nitrogen for metabolome and RNA-seq (Figure S1). For BTx406 and R9188, stem tissues from internodes No.2 to No.6 were sampled for brix measurement, whereas for RIO, internodes No.2 to No.12 were harvested. Except for the three plants used for measuring Brix, only upper internodes from the rest six plants per subplot were sampled, immediately frozen in liquid nitrogen and stored at -80℃ until processing. Tissues from each plant were stored individually.

Since sweet sorghum RIO flowered later than BTx406 and R9188 and experienced colder environmental conditions, its grain development appeared to be slower compared to those of BTx406 and R9188. Thermo time (TT) was estimated as the difference between the mean daily temperature and a base temperature of 10.8℃(Lafarge and Tardieu 2002). The thermos time from flowering to T3, T4 and T5 stages were 136, 192, 272 ℃d for BTx406, 136, 177 and 260 ℃d for R9188, and 73, 95 and 154 ℃d for RIO. Being consistent with the thermos time estimation, BTx406 and R9188 reached to soft and hard dough stages at T4 and T5, respectively, while RIO was in milk stage at T4 and in later stage of soft dough at T5.

**2. Brix measurement**

Samples of internode tissue were collected and transferred back to lab on ice. The juice from internode samples was extracted immediately according to Calvino et al. (2008). In addition, the concentrations of sucrose, glucose and fructose were determined for the upper internode samples (see “Metabolomics”). A list of traits, descriptions and their corresponding crop/plant ontology terms are provided in Table S1 (Shrestha et al. 2012; Cooper et al. 2018).

**3. Metabolomics**

The six plants in each subplot were used for metabolic profiling except for the three central plants used for Brix measurement. Among these six plants, three plants were pooled together to form a biological replicate for metabolome analysis. All the sampled tissues were maintained at -80℃ until processed. Equal amounts of internode tissues from three plants were sliced, pooled and ground in liquid nitrogen with pestle and mortar. The ground sample was divided into two fractions; one 100-mg fraction for the global unbiased metabolic profiling and one 300-mg fraction for targeted metabolic profiling. Metabolome sample preparation and analysis was performed at Metabolon Inc. (Research Triangle Park, NC) under a service contract with Syngenta.

**3.1. The untargeted metabolomic analysis.**

***Overview of the untargeted metabolomics analysis***

The global unbiased metabolomic platform is composed of four independent platforms: UHPLC-MS/MS optimized for basic species, UHPLC-MS/MS optimized for acidic species, polar LC platform (UHPLC(HILIC)-MS/MS) and GC-MS (Evans et al. 2009; Ohta et al. 2009) (Appendix S1). Briefly, each sample was extracted in methanol with recovery standards using an automated MicroLab STAR system (Hamilton). The resulting extract was divided into five fractions, with four fractions subjected to the four platforms, respectively, and one fraction reserved for backup. For LC methods, the analysis was carried out by using a Water Acquity UHPLC coupled to a Thermo Scientific Q-Exactive mass spectrometer equipped with an electrospray ionization source and Orbitrap mass analyzer. For GC-MS, samples were derivatized using bis-trimethyl-silyl-triflouroacetamide and then analyzed on a Thermo-Finnigan Trace DSQ fast-scanning single-quadrupole mass spectrometer. Known chemicals were identified by comparison to Metabolon’s library entries of purified standard compounds based on retention time/index (RI), mass to charge ratio (m/z), and MS/MS spectral data. A full description of the untargeted metabolomics method can be found under Supplemental Information. The missing values were imputed with the observed minimum for each specific metabolite. For data visualization, the raw area counts for each compound were median scaled (Data S2). Principle component analysis (PCA) and Pearson’s correlations between samples were performed with the metabolomics toolbox COVAIN (Sun et al., 2012) (Figure 1c). Significant differential metabolites were determined by both Welch two-sample t-test and COVAIN (details in Appendix S1).

***Sample accessioning.***

All samples were stored at – 80 ℃ on receipt and maintained at – 80 ℃ until processed. Each sample was accessioned into the Metabolon LIMS system with a unique identifier, which was used for tracking all sample handling, tasks, results, etc. The Metabolon LIMS system assigned all portions and all derived aliquots of samples their own unique identification numbers to track the relationships of these samples and their analysis tasks.

***Sample preparation.***

Samples were automatically prepared and extracted using methanol by MicroLab STAR system (Hamilton) to remove protein fraction and to maximize small molecule recovery. For quality control (QC) purpose, recovery standards were added before extraction. The extracted sample was divided into five aliquots: one for UHPLC-MS/MS (+ESI) analysis, one for UHPLC-MS/MS (-ESI) analysis, one for the LC polar platform, one for the GC-MS analysis and one aliquot stored for backup. To remove organic solvent, the samples were treated briefly by the TurboVap (Zymark) under nitrogen. For LC-based analyses, the samples were stored under nitrogen overnight, whereas for GC-MS analysis, the samples were dried under vacuum overnight.

***Quality assurance and quality control.***

To provide accurate and consistent metabolic profiling results, a variety of QC samples and standards were analyzed together with the experimental samples. For QC samples, a pooled matrix sample (also known as “CMRTX”) generated by taking a small volume of each experimental sample (or alternatively, use of pool of well-characterized human plasma, “MRTX”) were used as a technical replicate throughout the data set. Aliquots of ultra-pure water and solvents used in extraction served as process blank and solvent blank to remove any signals derived from artifacts, respectively. For QC standards, recovery standards were used to assess variability and verify performance of extraction and instrumentation; internal standards were used to evaluate the variability and performance of instruments; derivatization standards were used for assessing the variability of derivatization of samples for GS-MS platform.

For QC of the targeted metabolic profiling, the analysis was performed in 96-well plate format including six replicates of quality control (QC) samples and two calibration curves. The criteria for targeted metabolic profiling is %CV ≤ 20% for intra- and inter- run QC precision, which was evaluated by the six QC replicates (Table S2).

***Ultra-high performance liquid chromatography/mass spectroscopy (UHPLC-MS/MS).***

The LC-MS/MS platforms have been described in detail elsewhere (Evans et al., 2009; Ohta et al., 2009). The LC-MS/MS platforms were performed with a Waters ACQUITY UHPLC and a Thermo Scientific Q-Exactive high resolution/accuracy mass spectrometer coupled with a heated electrospray ionization (HESI-II) source and Orbitrap mass analyzer. The extracted samples were reconstituted in acidic or basic LC-compatible solvents. One aliquot of samples was analyzed using conditions optimized for acidic positive ion; the other aliquot was analyzed using conditions optimized for basic negative ion. The two platforms were performed in two independent injections using separate dedicated columns (Waters UHPLC BEH C18-2.1 x 100 mm, 1,7 µm). To ensure the consistency of injection and chromatographic performance, eight or more injection standards at fixed concentrations were used in LC-based platforms. The acidic extracts were eluted from a C18 column using a gradient consisting of water and methanol containing 0.1% formic acid, whereas the basic extracts were eluted from a C18 column using a gradient consisting of water and methanol containing 6.5 mM ammonium bicarbonate. A third aliquot for LC polar platform were analyzed using negative ionization conditions and was gradient eluted from HILIC column (Waters BEH Amide 2.1 x 150 mm, 1,7 µm) using water and acetonitrile containing 10 mM ammonium formate. The MS analysis alternated between MS and data-dependent MS2 scans using dynamic exclusion, and the raw data were collected ranging from 80-1000 m/z.

***Gas chromatography-mass spectroscopy (GC-MS).***

The GC-MS platform has been described in detail previously (Ohta et al., 2009). For the GC-MS platform, samples were vacuum-dried for over 18 h followed by derivatization under nitrogen using bis-trimethyl-silyl-triflouroacetamide. A 5% diphenyl/ 95% dimethyl-polysiloxane fused silica column (20 m x 0.18 mm ID; 0.18 µm film thickness) were used to separate the derivatized samples with helium as carrier gas and a temperature ramp from 60 ℃ to 340 ℃ in a 17.5 min period. The samples were analyzed on a Thermo Finnigan Trace DSQ fast-scanning single quadrupole mass spectrometer (MS) with an electron impact ionization. The MS was operated at unit mass resolving power with a scan range from 50 to 750 m/z.

***Accurate mass determination (LC/MS) and MS/MS fragmentation (LC/MS/MS) for structural elucidation.***

The LC-MS based platforms employ a Waters ACQUITY UHPLC and a Thermo-Finnigan OrbiElite mass spectrometer, which had a linear ion-trap frontend and an orbitrap mass spectrometer backend. Accurate mass measurements could be made on the parent ion and fragments with typical mass error less than 5 ppm. Fragmentation spectra were targeted.

***Bioinformatics.***

The informatics system includes four major components: the LIMS, the software for data collection and peak-identification, the software for QC, data processing and compound identification and a collection of data interpretation and visualization tools. The hardware and software foundations for these components were the LAN backbone and a database server running Oracle 10.2.0.1 Enterprise edition.

***LIMS.***

The Metabolon LIMS system enables fully auditable lab automation through a secure, easy-to-use and highly specialized system. The functions of the Metabolon LIMS system include sample accessioning, sample preparation, instrumental analysis and reporting and advanced data analysis, with subsequent software systems grounded in the LIMS data structures. Moreover, the LIMS system has been modified to leverage and interface with in-house information extraction and data visualization systems and other instrumentation and data analysis software from third parties.

***Data Extraction and Compound Identification.***

To build a web-service platform for Metabolon’s in-house hardware and software systems, the Microsoft’s .NET technologies were utilized and run on high-performance application servers and fiber-channel storage arrays in clusters. The Metabolon’s systems were used for raw data extraction, peak-identification and QC process. Biochemicals were identified by comparison to library entries of purified standards or recurrent unknown entities. Metabolon’s compound library is based on authenticated standards and contains information of all molecules in the library, including the retention time/index (RI), mass to charge ratio (m/z), and chromatographic data (MS/MS spectral data). In the Metabolon’s in-house library, more than 3300 commercially available purified standard compounds have been registered for distribution to both the LC-MS and GC-MS platforms for determination of their analytical characteristics. In addition, structurally unnamed biochemicals, which had been identified by their recurrent chromatographic and mass spectral data, have been registered in the library. These compounds could be identified in future if a matching purified standard is acquired.

To accurately identify biochemicals, three criteria were applied: retention index within a narrow RI window, accurate mass match to the library entries (+/- 0.005 amu), and the MS/MS forward and reverse scores between the experimental data and authentic standards. The MS/MS scores are based on a comparison of the ions present in the experimental spectrum to the ions present in the library spectrum. The use of all three criteria can be effective in distinguishing and differentiating biochemicals.

***Curation.***

To ensure the high quality of datasets for statistical analysis and data interpretation, a variety of curation processes were performed. They were designed to ensure data accuracy and consistency and to remove those representing system artifacts, mis-assignments and background noises. Proprietary visualization and interpretation software were employed by Metabolon’s data analysts to confirm the consistency of peak identification among samples. Metabolon data analysts checked and corrected, if necessary, library matching for each compound.

***Metabolite Quantification and Data Normalization.***

The area-under-the-curve (AUC) was used for peak quantification. To correct variations due to instrument inter-day tuning differences for studies spanning multiple days, data normalization was performed. Briefly, each compound was corrected in run-day blocks by registering the medians to equal one and normalizing each data point proportionately (termed as “block correction”).

***Instrument and process variability.***

To reflect instrument variation, several internal standards were added to each sample just prior to injection. Then the median relative standard deviations (RSD) of these internal standards were used for determining the platform variability. Additionally, the RSD of the pooled matrix samples (CMTRX) were also measured to reflect the total variability in the process of samples and in the quantification of endogenous metabolites within these experimental samples. The median RSD values were 5% for internal standards and 9% for endogenous metabolites.

***Determination of significant differential metabolites.***

To identify dynamic changes of metabolic pathways, metabolite abundance was pair-wise compared between time points within the varieties by Welch two-sample t-test with a stringent p-value <0.005 as a cutoff. The metabolome results were also subject to ANOVA analysis by COVAIN (p<0.05). The metabolites passed the two filters were considered as significant differential metabolites, while the metabolites failed to pass the filters were considered as no significant changes along the time course. The significant differential metabolites were generally classified based on their trends: up-regulated, down-regulated and other trends (for instance, up-and-down or down-and-up).

**4. Transcriptome sequencing using Illumina Nextseq 500 platform.**

The nine plants per subplot were pooled together to represent a biological replicate for RNA-seq and the samples for each time point per genotype were performed in triplicates, corresponding to three blocks in the field design. A total of 12 stem samples for RNA-seq were collected with three replicates, including four samples collected at T1, T2, T3, and T4 time points from each of the three genotypes. Frozen internode tissues were pulverized into fine powder in liquid nitrogen with a mortar and pestle. For each sample, equal amounts of tissues from internode #2, #3 and #4 were mixed together for RNA extraction. Total RNA was isolated using TRIzol reagent and purified using a PureLink RNA Kit (Ambion, Life Technologies).

Standard protocols for the Illumina Nextseq 500 platform were used for construction of the sorghum mRNA libraries. RNA-seq libraries were sequenced to generate 150-bp pair-end reads. For sequence quality control, cutadapt and FASTX-Toolkit (http://hannonlab.cshl.edu/fastx_toolkit/) were used (Martin, 2011). First, adaptors were cut, the low-quality base pairs (Phred quality ≤14) trimmed from the 3’ end of each sequence and the filtered sequence discarded, when it was shorter than 35 bp. The quality-filtered reads were mapped to the sorghum reference genome of BTx623 (Sbicolor_v2.1_255) using TopHat v2.0.14 with a maximum mismatch of 15 bp and default settings for other parameters (Paterson et al., 2009; Trapnell et al., 2012). Only uniquely-mapped reads were retained and the read counts that aligned to the 33,032 gene models annotated in Phytozome were calculated by using the ‘HTseq’ software (http://www-huber.embl.de /users/anders/HTSeq/) (Anders et al. 2015). RPKM (reads per kilobase of exon per million mapped sequence reads) values were calculated for each gene model. Genes that met the following criteria were considered as expressed in a time point: (1) at least 10 reads mapped to a gene in each of the three replicates; (2) the mean RPKM at a time point should be ≥1. The Spearman correlation coefficients between biological replicates were calculated using log2-transformed RPKM+1 values (Supplementary Tables 2 &3). Gene expression levels (RPKM) were also normalized to z-score across genotypes and time points with the equation z_i_ = (x_i_ -mean)/SD, where xi is the expression level of a given gene in individual samples, mean is the averaged expression level of the gene, and SD is the standard deviation of RPKM values of the gene. For hierarchical clustering, average linkage clustering was performed with the R package “amap” using Pearson’s correlation as a distance measure and RPKM-normalized z-score as input. The differentially expressed genes (DEGs) were determined with DEseq and edgeR, respectively, by comparing the read count data between samples (Anders and Huber 2010; McCarthy et al., 2012). For DEseq, the significance threshold for defining a DEG was fold change ≥ 2 and *q* value < 0.05. For edgeR, the significance threshold was fold change ≥ 2 and a false discovery rate (*FDR*)-adjusted *P* value < 0.05. For each genotype, the genes identified by either DEseq or edgeR with their default settings and the above-mentioned cutoffs were considered as DEGs and combined to generate a list of DEGs. We employed two independent and complementary approaches to analyze the transcriptome data: (i) Co-expression networks were constructed for each genotype by using the DEGs generated from pair-wise comparison between time points within the same genotypes, and then the enriched biological functions were used to represent co-expression modules and compared between genotypes; (ii) DEGs were calculated between genotypes within the same time point and the differences in RIO vs R9188/BTx406 and RIO/R9188 vs BTx406 were dissected.

**3. Weighted gene co-expression network analysis and module preservation analysis.**

Co-expression network analysis was performed using the R package WGCNA (Langfelder and Horvath 2008). To identify genotype-specific patterns of highly-correlated genes, co-expression networks for each genotype were constructed independently. DEGs were calculated for each genotype by comparing any two of the four time points within the same genotypes, resulting in 3,250, 8,336 and 9,996 DEGs in RIO, R9188 and BTx406, respectively (pair-wise comparison). The expression matrixes of DEGs in RIO, R9188 and BTx406 were used for co-expression network analysis individually (Supplementary Dataset 3). Briefly, expression correlation between genes were calculated using a robust biweight-midcorrelation method (Langfelder and Horvath 2012) and raised to a soft threshold power, in which the co-expression network fitted to scale-free topology and had low mean connectivity (Supplementary Fig. 7). Next, a signed-hybrid weighted correlation network was used to identify modules of interconnected genes with high topological overlap (TO). Co-expression modules were defined as branches of a hierarchical clustering tree, which was conducted using dynamic tree cut method with a minimum module size of 50 genes. The expression pattern of each module was summarized by calculating it as the first principle component of the standardized expression patterns of the module genes (referred to as module eigengene, Fig. 2). Pairs of closely related modules (module eigengene correlation > 0.85) were merged. The transformation of expression correlation values to TO co-expression values effectively captures the relationships among neighborhoods of genes, making it a more robust and accurate approach than traditional clustering methods, which is simply based on similarities among gene expression (Allen et al., 2012).

To quantify the intramodular connectivity of genes in each module, module membership (kME) was calculated as the correlation between gene expression profile and its module eigengene. It has been demonstrated that the module membership is highly correlated with the intramodular connectivity, referred to as eigengene-based connectivity (https://labs.genetics.ucla.edu/horvath/ CoexpressionNetwork/Rpackages/WGCNA/Tutorials). Genes with the highest kME values are referred to as module hub genes, which are highly connected, located inside the module and may play important roles in biology (Langfelder et al., 2013). Metabolite profiles were also related to modules using module eigengene values.

***Module preservation analysis.***

Co-expression networks and modules were characterized to address whether modules were robustly identified in each genotype by resampling 10 out of the 12 (~83%) RNAseq libraries in each genotype and calculating the module preservation parameters for each module 20 times (Supplementary Fig. 8). Here, two composite module preservation statistics were used, median rank and Z_summary_. Median rank is a rank-based measure considering both module density and connectivity, and does not depend on module size, whereas Z_summary_ is a permutation-based statistic also measuring both module density and connectivity and might be affected by module size (Langfelder and Horvath 2011). Z_summary_ > 2 indicates moderate preservation and Z_summary_ > 10 means high preservation, whereas on the contrary the smaller a median rank is, the more preserved a module is. The significance for Z_summary_ and median rank between a module and non-module genes (grey module) was determined using Welch’s *t*-test.

**4. Functional annotations for characterizing RNA-seq data.**

The kME distribution of each module was visualized (Supplementary Fig.10). Genes with kME ≥ 0.7 were defined as module genes (ranging from 81% to 97% of genes among modules), whereas genes with kME< 0.7 were marginal genes. Then both module genes and hub genes were functionally characterized and enriched by hypergeometric tests using clusterProfiler (Yu et al., 2012) with all expressed genes in their own genotypes as backgrounds. To control false discovery rate, functional terms with a *P* value < 0.05 and a Benjamini-Hochberg-adjusted *P* value (*q* value) < 0.2 were significantly enriched. The minimum numbers of mapped genes by a functional term was set at 3. The following sorghum annotation resources were used for functional enrichment analysis.

***Gene ontology (GO) analysis.***

GO annotations were downloaded from the Sbicolor_v2.1_255 reference genome (Phytozome) and AgriGO, respectively (Du et al., 2010). Both GO annotations were merged into one non-redundant GO annotation file for GO enrichment analysis. Only the biological process (BP) and molecular function (MF) GO terms were kept, as these two categories are more informative than the cellular component category.

***MapMan annotation.***

The sorghum MapMan annotation was downloaded (http://mapman.gabipd.org/web/) and used for functional annotation (Usadel et al., 2009). In MapMan annotation, a transcript’s ID is assigned to a MapMan pathway (represented by a hierarchical bincode), so that we first converted transcript IDs into their gene IDs. In addition to the original MapMan annotation, Mapman annotation were simplified as follows: all genes were assigned to all corresponding parental bincodes and only kept the 2^nd^ level MapMan annotations to capture the brief changes in functional classifications. The annotated bincodes with the functional terms of “not assigned, unknown” or “not assigned, no ontology” were excluded in this study. Both the original MapMan annotation and 2^nd^ level MapMan annotation were used for functional enrichment, respectively.

***Metabolism gene annotations.***

To understand the changes of genes involved in metabolism in our RNA-seq dataset, the metabolism annotations from KEGG and Plant Metabolic Network (PMN) (Chae et al., 2014) were used, which were downloaded from the Sorghum Functional Genomics Database (sorghumFDB) (Tian et al., 2016).

***Transcription factor (TF) annotation.***

Transcription factor annotations for sorghum were downloaded from GRASSIUS (Yilmaz et al., 2009) and sorghum FDB (Tian et al., 2016), the latter of which collected transcription factor information from PlantTFDB (Jin et al., 2014) and PlnTFDB (Riano-Pachon et al., 2007). These annotations were merged together non-redundantly and used to identify TFs in each module, containing 2,537 TFs from 94 families.

***Annotation of target genes: Starch, sucrose metabolism and glycolysis.***

Annotations for genes related to sucrose metabolism, starch synthesis/degradation and some steps of glycolysis were identified from different resources: PMN, KEGG and the literature (Campbell et al., 2016). The genes reported by literature mainly belong to starch synthesis and sucrose metabolism genes, and homologs of these gene families were further searched in the sorghum genome with BLAST in Phytozome. A total of 188 sorghum gene models were annotated in this category, including 69 gene models related to sucrose metabolism, 28 to starch synthesis, 59 to starch degradation and 32 to glycolysis (Supplementary Dataset 5).

***Annotation of target genes: Putative sugar transporters.***

Genes encoding *Sucrose Transporters* (*SUTs*) and *Sugars Will Eventually be Exported Transporters* (*SWEETs*) were collected from literature sources (Bihmidine et al., 2015; Mizuno et al., 2016). Genes encoding *Tonoplast Sugar Transporters* (*TSTs*, also known as *Tonoplast Monosaccharide Transporters*) were according to Bihmidine et al. (2016).

***Annotation of target genes: Cell wall-related genes.***

Cell wall related genes involved in polysaccharide synthesis, reassembly and degradation were obtained from additional literature sources (Rai et al., 2016), as were three genes families functioning in monolignol pathway, 4-coumarate:CoA ligase (4CL), cinnamyl alcohol dehydrogenase (CAD) and cinnamyl-CoA reductase (CCR) (Saballos et al., 2009, 2012; Barakat et al., 2011). The other families of cell wall related genes were collected from Cell Wall Genomics (https://cellwall.genomics.purdue.edu/) (Carpita et al., 2001).

**5. Enrichment Analysis of sugar-related gene sets.**

Literature sources were used for gene lists positively and negatively correlated to sugar signals in *Arabidopsis*. Six experiments were identified: Experiment 1, genes up- or down-regulated by T6P determined in *Arabidopsis* (gene sets A, B, C and D) (Zhang et al., 2009); experiment 2, *Arabidopsis* SnRK1 (KIN10) inducible and repressible genes (gene sets E, F, G and H) (Baena-Gonzalez et al., 2007); experiment 3, inducible and repressible genes by sucrose starvation in *Arabidopsis* suspension cells (gene sets I and J) (Contento et al., 2004); experiment 4, inducible and repressible genes by sucrose starvation in *Arabidopsis* seedlings (gene sets K and L) (Gonzali et al., 2006); experiment 5, glucose and ABA responsive genes in Arabidopsis seedlings (gene sets M, N, O and P) (Li et al., 2006), and experiment 6, glucose inducible and repressible genes, determined by comparing wildtype and tor mutant *Arabidopsis* (gene sets Q, R, S and T) (Xiong et al., 2013). All *Arabidopsis* gene sets and the non-redundant gene sets (Supplementary Fig. 15) are provided in Supplementary Dataset 6. To determine if the differentially expressed genes in sorghum genotypes have significant overlap with the *Arabidopsis* sugar/sugar signal related genes, all sorghum gene models were first converted into *Arabidopsis* gene models based on the functional annotation file in Phytozome. The maize gene sets that are regulated by T6P signaling were downloaded from Oszvald et al. (2018). Only sorghum orthologs were kept and used for overlapping test based on the maize-sorghum orthologous information (Zhang et al. 2017). The overlap between sorghum modules and the *Arabidopsis* gene sets were identified and their statistical significance calculated from the hypergeometric distribution (Fig. 3; Supplementary Fig.15).

**6. Identification of genes in trehalose pathway and phylogenetic analysis.**

The trehalose-6-phosphate (TPS) and trehalose-6-phosphate phosphatase (TPP) families in the trehalose biosynthetic pathway were identified in this study. TPS and TPP genes in sorghum were identified using a name search in Phytozome and BLAST with maize sequences (Henry et al., 2014). Twelve TPS genes and 12 TPP genes were identified in sorghum. Specifically, the gene model Sobic.005G213832 (TPS), which only existed in sorghum v3.1 annotation but not in v2.1 annotation, was used in the following sequence comparison and phylogenetic analysis. The deduced protein sequences of primary transcripts were compared with those of maize, rice, and *Arabidopsis* (Lunn et al., 2007; Ge et al, 2008; Henry et al., 2014). Sequence alignments were conducted using ClustalX and neighbor joining (NJ) phylogenetic trees were generated using MEGA v4 with JTT protein substitution model and 1,000 times bootstrap (Tamura et al., 2007). The clades and subclades in the TPS and TPP phlyogenetic trees were identified based not only on the tree structures but also previously published phlyogenetic studies of TPS and TPP (Vandesteene et al., 2008, 2012; Henry et al., 2014). The nomenclature of sorghum TPS and TPP were in accordance with those in maize. The 1,000 bp sequences upstream from the TPP primary transcripts were retrieved from Phytozome and *cis*-regulatory elements were identified using PlantPAN 2.0 (Chow et al., 2016), showing that several bZIP -binding elements were found in the promoters of the T6P-associated TPP genes (Supplementary Fig. 20).

**7. RNAseq-based SNP calling and Identification of introgression regions.**

Uniquely-mapped reads were used for SNP calling. The SNPs were called relative to the sorghum reference genome (BTx623, Sbicolor_v2.1_255) for each genotype individually by using the mpileup functions in SAMTools software with default parameters (Li, 2011), yielding 992,087, 724,912 and 793,113 raw SNPs in BTx406, RIO and R9188, respectively. A SNP was not allowed to be an ambiguous base (e.g. SNP ≠ “N”). The SNPs from the three genotypes were merged together to produce a union of 2,192,460 raw SNPs. A multiple-step procedure was used to detect high-quality SNPs. First, biallelic and homogeneous SNPs were kept. Homogeneous SNPs were defined as those identified in all three replicated RNA-seq samples per time point and genotype. Second, SNPs with an alternative allele frequency ≥ 95% were filtered, which were considered as homozygous SNPs. Third, the homozygous SNPs in each genotype, for a depth of an alternative allele, at least 4 reads per library were filtered and a total of such a depth of at least 12 reads per time point. Fourth, by comparing SNPs of R9188 with those of RIO and BTx406, we separated SNPs into two groups, group-1 SNPs have the same alleles as RIO whereas group-2 SNPs have the same alleles as BTx406. To identify the R9188 chromosomal regions introgressed from BTx406, the sorghum genome was divided into 100-kb bins and assigned SNP positions into these bins. The SNP density of a group in each bin is significantly higher than random by using bootstrapping simulation for the two SNP groups (SNPs from RIO, and SNPs from BTx406). For each bin, the threshold was set that the real number of SNP should be more than 3 standard deviations above the simulated mean of number of SNP. The parental origins of bins were assigned based on the statistical test and the results were plotted (Fig. 4). Supplementary Dataset 7 shows high-quality SNPs were originating from RIO (14,245 SNPs) and BTx406 (7,003 SNPs). Moreover, the 7,003 SNPs from BTx406 define the chromosomal regions introgressed from BTx406 were represented by 1,602 genes. The high-quality SNPs were annotated based on their genomic locations and their potential impacts were predicted by using the SnpEff software (Cingolani et al., 2012). The introgression regions were visualized by circos plot (Krzywinski et al. 2009; Figure 6). Further, the sugar traits-related QTL regions which were identified in previous studies by using simple sequence repeats (SSR) markers and GWAS were located to the sorghum reference genome by mapping SSR markers closely associated with QTLs or by identifying the GWAS association peaks with 150-kb linkage disequilibrium decay (Figure 6 and Table S9; Natoli et al. 2002; Ritter et al. 2008; Shiringani et al. 2010; Guan et al. 2011; Mocoeur et al. 2015; Burks et al. 2015; Brenton et al. 2016). The 150-kb distance of LD decay in sorghum was used based on several studies in which LD decay estimates were calculated by using the resequencing and genotyping-by-sequencing data (Mace et al. 2013; Morris et al. 2013; Yu et al. 2016). The LD decay estimate used here is higher than previously published values, likely due to low genome coverage of markers and fewer genotypes in previous studies (Hamblin et al. 2005; Bouchet et al. 2012).

**8. Identification of candidate genes in the introgression regions**

To identify candidates involved in the T6P signaling networks and/or central metabolism, a multiple-step filtering procedure was applied. First, Pearson’s correlation co-efficiencies were calculated of the introgressed DEGs for T6P or sucrose in the three genotypes. Specifically, if a gene strongly correlates with the sugar metabolites in a genotype (Pearson’s |r| >=0.6), it must be a DEG in that genotype, otherwise the gene will be removed in further analysis. Second, the genes that exhibited differential correlation patterns between RIO and BTx406/R9188 were identified based on the following distinction: Pearson’s |r| >=0.6 as a strong correlation, -0.3 < r <0.3 as not correlated, and the rest as weak correlation. A differential correlation pattern was defined as a strong correlation in RIO but an either non-correlation or inverse correlation in BTx406 and R9188, and vice versa. Third, genes with low expression levels (maximum RPKM ≤ 5) were removed. Fourth, the expression profiles of candidate genes were manually checked in all three genotypes to avoid any false-positives by calculating correlations. Any genes with similar expression tendencies in all three genotypes were removed.

To identify robust sucrose-associated genes in R9188, only genes showing strong correlation with sucrose in all three genotypes were considered, because similar sucrose abundances between R9188 and RIO from T1 to T4 made it difficult to find genes with differential correlation to sucrose between RIO and BTx406/R9188.

**Supplemental References:**

Allen, J.D., Xie, Y., Chen, M., Girard, L., and Xiao G. (2012) Comparing statistical methods for constructing large scale gene networks. *PLoS ONE*. **7**, e29348.

Anders, S. and Huber, W. (2010) Differential expression analysis for sequence count data. *Genome Biol*. **11**, R106.

Anders, S., Pyl, P. T., and Huber, W. (2015) HTSeq-a Python framework to work with high-throughput sequencing data. *Bioinformatics*. **31**,166-169.

Baena-Gonzalez, E., Rolland, F., Thevelein, J.M., and Sheen, J. (2007) A central integrator of transcription networks in plant stress and energy signaling. *Nature*. **448**,938-943.

Barakat, A., Yassin, N.B., Park, J.S., Choi, A., Herr, J., and Carlson, J.E. (2011) Comparative and phylogenomic analyses of cinnamoyl-CoA reductase and cinnamoyl-CoA-reductase-like gene family in land plants. *Plant Sci*. **181**, 249-257.

Bihmidine, S., Julius, B.T., Dweikat, I., and Braun, D.M. (2016) *Tonoplast Sugar Transporters* (*SbTSTs*) putatively control sucrose accumulation in sweet sorghum stems, *Plant Signaling Behavior*. **11**, e1117721.

Bouchet, S., Pot, D., Deu, M., Rami J.F., Billot, C., Perrier, X., Rivallan, R., Gardes, L., Xia, L., Wenzl, P., Killan, A., Glaszmann, J.C. (2012) Genetic structure, linkage disequilibrium and signature of selection in Sorghum: lessons from Physically anchored DArT markers. *PLoS ONE*, **7**, e33470.

Campbell, B.C., Gilding, E.K., Mace, E.S., Tai, S., Tao, Y., Prentis, P.J., Thomelin, P. *et al.* (2016) Domestication and the storage starch biosynthesis pathway: signatures of selection from a whole sorghum genome sequencing strategy. *Plant Biotech. J*. **14**,2240-2253.

Carpita, N.C., Tierney, M., and Campbell, M. (2001) Molecular biology of the plant cell wall: searching for the genes that define structure, architecture and dynamics. *Plant Mol. Biol*. **47**,1-5.

Cingolani, P., Platts, A., Wang le, L., Coon, M., Nguyen, T., Wang, L., Land, S.J., Lu, X., Ruden, D.M. (2012) A program for annotating and predicting the effects of single nucleotide polymorphisms, SnpEff: SNPs in the genome of Drosophila melanogaster strain w1118; iso-2; iso-3. *Fly (Austin*). **6**,80-92.

Chow, C., Zheng, H.Q., Wu, N.Y., Chien, C.H., Huang, H.D., Lee, T.Y., Chiang-Hsieh, Y.F. *et al.* (2016) PlantPAN 2.0: an update of plant promoter analysis navigator for reconstructing transcriptional regulatory networks in plants. *Nucleic Acids Res*. **44**, D1154-D1160.

Contento, A.L., Kim, S.J., and Bassham, D.C. (2004) Transcriptome profiling of the response of *Arabidopsis* suspension culture cells to Suc starvation. *Plant Physiol*. **135**,2330-2347.

Cooper, L., Meier, A., Laporte, M.A., Elser, J.L., Mungall, C., Sinn, B.T., Cavaliere, D. et al. (2018) The Planteome database: an integrated resource for reference ontologies, plant genomics and phenomics. *Nucleic Acids Res*. **46**, 1168-1180.

Ge, L.F., Chao, D.Y., Shi, M., Zhu, M.Z., Gao, J.P., and Lin, H.X. (2008) Overexpression of the trehalose-6-phosphate phosphatase gene *OsTPP1* confers stress tolerance in rice and results in the activation of stress responsive genes. *Planta*. **228**,191-201.

Gonzali, S., Loreti, E., Solfanelli, C., Novi, G., Alpi, A., and Perata, P. (2006) Identification of sugar-modulated genes and evidence for *in vivo* sugar sensing in *Arabidopsis*. *J. Plant Res*. **119**, 115–123.

Hamblin, M.T., Salas Fernandez M.G., Casa, A.M., Mitchell, S.E., Paterson, A.H., Kresovich, S. (2005) Equilibrium processes cannot explain high levels of short- and medium-range linkage disequilibrium in the domesticated grass Sorghum bicolor. *Genetics*, **171**, 1247-1256.

Krzywinski, M., Schein., J.E., Birol, I., Connors, J., Gascoyne, R., Horsman, D., Jones, S.J. *et al*. (2009) Circos: an information aesthetic for comparative genomics. *Genome Res*. **19**, 1639-1645.

Langfelder, P., Mischel, P.S., and Horvath, S. (2013) When is hub gene selection better than standard meta-analysis? *PLoS ONE*. **8**(4): e61505.

Lafarge, T., Tardieu, F. (2002) A model coordinating the elongation of all leaves of a sorghum cultivar was applied to both Mediterranean and Sahelian conditions. *J. Exp. Botany*. **53**, 715-725.

Li, Y., Lee, K.K., Walsh, S., Smith, C., Hadingham, S., Sorefan, K., Cawley, G. *et al.* (2006) Establishing glucose- and ABA-regulated transcription networks in *Arabidopsis* by microarray analysis and promoter classification using a Relevance Vector Machine. *Genome Res*. **16**, 414–427.

Lunn, J.E. (2007) Gene families and evolution of trehalose metabolism in plants. *Funct. Plant Biol*. **34**, 550-563.

Mace, E.S., Tai, S., Gilding, E.K., Li, Y., Prentis, P.J., Bian, L., Campbell, B.C., Hu, W., Innes, D.J., Han, X., Cruickshank, A., Dai, C., Frere, C., Zhang, H., Hunt, C.H., Wang, X., Shatte, T., Wang, M., Su, Z., Li, J., Lin, X., Godwin, I., Jordan, D., Wang, J. (2013) Whole-genome sequencing reveals untapped genetic potential in Africa’s indigenous cereal crop sorghum. *Nature Communications*. **4**, 2320.

Martin, M. (2011) Cutadapt removes adapter sequences from high-throughput sequencing reads. *EMBnet J*. **17**,10–12.

Martin, A.P., Palmer, W.M., Brown, C., Abel, C., Lunn, J.E., Furbank, R.T., and Grof, C.P. (2016) A developing *Setaria viridis* internode: an experimental system for the study of biomass generation in a C4 model species. *Biotechnol. Biofuel*. **9**,45.

McCarthy, D.J., Chen, Y., and Smyth, K.G. (2012) Differential expression analysis of multifactor RNA-Seq experiments with respect to biological variation. *Nucleic Acids Res*. **40**(10),4288-4297.

Morris, G.P., Ramu, P., Deshpande, S.P., Hash, C.T., Shah, T., Upadhyaya, H.D., Riera-Lizarazu, O., Brown, P.J., Acharya, C.B., Mitchell, S.E., Harriman, J., Glaubitz, J.C., Buckler, E.S., Kresovich, S. (2013) Population genomic and genome-wide association studies of agroclimatic traits in sorghum. *Proc. Natl. Acad. Sci. USA.* **110**, 453-458.

Rai, K.M., Thu, S.W., Balasubramanian, V.K., Cobos, C.J., Disasa, T., and Mendu, V. (2016) Identification, characterization, and expression analysis of cell wall related genes in *Sorghum bicolor* (L.) Moench, a food, fodder, and biofuel crop. *Front. Plant Sci*. **7**,1287.

Ritter, K., Chapman, S., Jordan, D., Godwin, I., McIntyre, L. (2004) Investigating the use of sweet sorghum as a model for sugar accumulation in sugarcane. 4^th^ International Crop Science Congress, Brisbane, Austrlia.

Ritter, K. (2007) An investigation into the genetics and physiology of sugar accumulation in sweet sorghum as a potential model for sugarcane. University of Queensland.

Saballos, A., Ejeta, G., Sanchez, E., Kang, C., and Vermerris, W. (2009) A Genome wide analysis of the cinnamyl alcohol dehydrogenase family in sorghum [*Sorghum bicolor* (L.) Moench] identifies *SbCAD2* as the *brown midrib6* gene. *Genetics*. **181**, 783-795.

Saballos, A., Sattler, S.E., Sanchez, E., Foster, T.P., Xin, Z., Kang, C., Pedersen, J.F. *et al.* (2012) *Brown midrib2* (*Bmr2*) encodes the major 4-coumarate: coenzyme A ligase involved in lignin biosynthesis in sorghum (*Sorghum bicolor* (L.) Moench). *Plant J*. **70**, 818-830.

Shrestha, R., Matteis, L., Skofic, M., Portugal, A., McLaren, G., Hyman, G., Elizabeth, A. (2012) Bridging the phenotypic and genetic data useful for integrated breeding through a data annotation using the Crop Ontology developed by the crop communities of practice. *Front. Physiol*. **3**, 326.

Sun, X., and Weckwerth, W. (2012) COVAIN: a toolbox for uni- and multivariate statistics, time series and correlation network analysis and inverse estimation of the differential Jacobian from metabolomics covariance data. *Metabolomics*. **8**, S81–S93.

Tamura, K., Dudley, J., Nei, M., and Kumar, S. (2007) MEGA4: molecular evolutionary genetics analysis (MEGA) software version 4.0. *Mol Biol Evol*, **24**: 1596–1599.

Trapnell, C., Roberts, A., Goff, L., Pertea, G., Kim, D., Kelley, D.R., Pimentel, H. *et al.* (2012) Differential gene and transcript expression analysis of RNA-seq experiments with TopHat and Cufflinks. *Nat Protocols*. **7**, 562-579.

Xiong, Y., McCormack, M., Li, L., Hall, Q., Xiang, C., and Sheen, J. (2013) Glucose–TOR signaling reprograms the transcriptome and activates meristems. *Nature*. **496**,181-187.

Yu, X., Li, X., Guo, T., Zhu, C., Wu, Y., Mitchell, S.E., Roozeboom, K.L., Wang, D., Wang, M., Pederson, G.A., Tesso, T.T., Schnable, P.S., Bernardo, R., Yu, J. (2016) Genomic prediction contributing to a promising global strategy to turbocharge gene banks. *Nature Plants*. **2**, 16150.

Zhang, Y., Primavesi, L.F., Jhurreea, D., Andralojc, P.J., Mitchell, R.A., Powers, S.J., Schluepmann, H. *et al.* (2009) Inhibition of SNF1-related protein kinase1 activity and regulation of metabolic pathways by trehalose-6-phosphate. *Plant Physiol*. **149**,1860-1871.

Zhang, Y., Ngu, D.W., Carvalho, D., Liang, Z., Qiu, Y., Roston, R.L., and Schnable, J.C. (2017) Differentially regulated orthologs in sorghum and the subgenomes of maize. *Plant Cell*. **29**, 1938-1951.
