# Supplementary material for: Transcriptome and metabolome reveal distinct carbon allocation patterns during internode sugar accumulation in different sorghum genotypes
Source: Plant Biotechnol J. 2018 Sep 15;17(2):472–87. doi: 10.1111/pbi.12991 (PMC6335075; doi:10.1111/pbi.12991)
Supplement: Supplementary file 2 — Table S9 Analysis of the TPS and Haloacid Dehalogenase (HAD) domains and conserved amino acids required for TPS activities in deduced TPS proteins from E. coli, yeast, Arabidopsis, rice, maize and sorghum. Table S10 Details of forty‐seven differentially expressed candidate genes identified in the introgressed regions of R9188 with expression levels and annotation information. Table S11 Information about QTLs associated with plant height, flowering time, glucose concentration, juice volume, juice weight, non‐fibrous carbohydrates and sugar contents, which were identified by previous studies. [file PBI-17-472-s003.pdf]

Title: Transcriptome and metabolome reveal distinct carbon allocation patterns during internode sugar accumulation in different sorghum genotypes.

Authors: Yin Li, Wenqin Wang, Yaping Feng, Min Tu, Peter E. Wittich, Nicholas J. Bate, Joachim Messing

Running head: transcriptome and metabolome in sorghum internode

TableS9. Analysis of the TPS and Haloacid Dehalogenase (HAD) domains and conserved amino acids required for TPS activities in the deduced TPS proteins from E.coli, yeast, Arabidopsis, rice, maize and sorghum.

**Domain**	**TPS domain**																					**HAD domain**		
**substrate/motif**	**UDPG**													**G6P**								**T6P**		
substrate/motif	Uracil	Ribose	Ribose	Phosphate	Phosphate	Phosphate	Phosphate	Phosphate	Glucose	Glucose	Glucose	Glucose	Glucose	Glucose	Glucose	Glucose	Glucose	Phosphate	Phosphate	Phosphate	Phosphate	Motif I	Motif II	Motif III
otsA	F340	G22	E370	R263	K268	N365	L366	V367	H155	Q186	D362	M364	N365	R10	W86	D131	I156	R10	Y77	H133	R301	n	n	n
ScTPS1	I	G	E	R	K	N	L	V	H	H	D	M	N	R	W	D	T262	R	Y	H	R	n	n	n
ScTPS2	I	A	E	R	R	N	T	T	H	Q	D	M	N	Q	W	D	A	Q	W	Y	A	y	y	y
AtTPS1	L446	G120	E476	R369	K374	N471	L472	V473	H261	H292	D468	M470	N471	R100	W185	D237	T262	R100	Y176	H239	R407	n	y	n
AtTPS2	V	G	E	R	K	N	L	V	H	H	D	M	N	R	W	D	S	R	Y	H	R	n	n	n
AtTPS3	I	G	E	R	K	N	L	V	H	H	D	M	N	S	W	D	T	S	Y	H	R	n	n	n
AtTPS4	V	G	E	R	K	N	L	V	H	H	D	L	N	R	W	D	S	R	Y	H	R	n	n	n
AtTPS5	L	S	E	D	K	N	L	I	H	H	D	M	N	Q	W	D	S	Q	Y	H	R	n	y	y
AtTPS6	L	S	E	D	K	N	L	I	H	H	D	M	N	E	W	D	S	E	Y	H	R	y	n	y
AtTPS7	V	S	E	D	K	N	L	T	H	H	D	M	N	R	W	D	S	R	Y	H	R	y	y	y
AtTPS8	V	S	K	D	K	N	L	V	H	H	D	M	N	M	W	D	S	M	Y	H	R	y	y	y
AtTPS9	V	S	K	D	K	N	L	V	H	H	D	M	N	M	W	D	S	M	Y	H	R	y	y	y
AtTPS10	V	S	K	D	K	N	L	V	H	H	D	M	N	F	W	D	S	F	Y	H	R	y	y	y
AtTPS11	V	S	K	D	K	N	L	V	H	H	D	M	N	Q	W	D	S	Q	Y	H	R	n	y	y
OsTPS1	L	G	E	R	K	N	L	V	H	H	D	M	N	R	W	D	T	R	Y	H	R	n	y	n
OsTPS5	V	A	I	D	K	N	R	I	H	H	D	L	N	R	W	D	S	R	Y	H	R	y	y	y
OsTPS7	I	A	E	D	K	N	L	V	H	Q	D	M	N	F	W	D	S	F	Y	H	R	y	y	n
OsTPS8	M	A	F	D	K	N	R	I	H	H	D	L	N	R	W	D	S	R	Y	H	R	y	y	y
OsTPS2	V	S	E	D	K	N	L	T	H	H	D	M	N	Q	W	D	S	Q	Y	H	R	y	y	y
OsTPS6	V	S	E	D	K	N	L	T	H	H	D	M	N	Q	W	D	S	Q	Y	H	R	y	y	y
OsTPS11	I	Q	E	D	K	N	L	I	H	Q	D	M	N	F	W	D	S	F	Y	H	R	y	y	n
OsTPS3	L	S	E	D	K	N	L	T	H	H	D	M	N	T	W	D	S	T	Y	H	G	y	y	y
OsTPS4	L	S	E	D	K	N	L	I	H	H	D	M	N	H	W	D	S	H	Y	H	R	y	y	y
OsTPS9	V	A	F	D	K	N	R	I	H	H	D	L	N	R	W	D	S	R	Y	H	R	y	y	y
OsTPS10	L	S	E	D	K	N	L	I	H	H	D	M	N	Q	W	D	S	Q	Y	H	R	y	n	y
ZmTPS I 1.1	L	G	E	R	K	N	L	V	H	H	D	M	N	R	W	D	T	R	Y	H	R	n	y	n
ZmTPS I 1.2	L		E	R	K	N	L	V			D	M	N								R	n	y	n
ZmTPS II 2.1	L	S	E	D	K	N	L	I	H	H	D	M	N	Q	W	D	S	Q	Y	H	R	y	n	
ZmTPS II 2.2	L	S	E	D	K	N	L	I	H	H	D	M	N	Q	W	D	S	Q	Y	H	R	y	y	y
ZmTPS II 3.1	V	S	E	D	K	N	L	T	H	H	D	M	N	Q	W	D	S	Q	Y	H	R	y	y	y
ZmTPS II 3.2	V	S	E	D	K	N	L	T	H	H	D	M	N	Q	W	D	S	Q	Y	H	R	y	y	y
ZmTPS II 3.3	V	S	E	D	K	N	L	T	H	H	D	M	N	Q	W	D	S	Q	Y	H	R	y	y	y
ZmTPS II 4.1	I	Q	E	D	K	N	L	I	H	Q	D	M	N	F	W	D	S	F	Y	H	R	y	y	n
ZmTPS II 4.2	I	A	E	D	K	N	L	V	H	Q	D	M	N	F	W	D	S	F	Y	H	R	y	y	n
ZmTPS II 4.3	I	S	E	D	K	N	L	V	H	Q	D	M	N	F	W	D	S	F	Y	H	R	y	y	n
ZmTPS II 5.1																						n	n	y
ZmTPS II 5.2	L	A	I	D	N	N	R	T	H	Q	D	L	N	R	W	D	S	R	Y	H	R	y	y	y
ZmTPS II 5.3	V	T	I	D	K	N	R	I	H	H	D	L	N	R	W	D	S	R	Y	H	R	y	y	y
ZmTPS II 5.4	V	T	I	D	K	N	R	I	H	H	D	L	N	R	W	D	S	R	Y	H	R	y	y	y
SbTPS I	L	G	E	R	K	N	L	V	H	H	D	M	N	R	W	D	T	R	Y	H	R	n	y	n
SbTPS II 2.1	L	S	E	D	K	N	L	I	H	H	D	M	N	Q	W	D	S	Q	Y	H	R	y	n	y
SbTPS II 2.2	L	S	E	D	K	N	L	I	H	H	D	M	N	Q	W	D	S	Q	Y	H	R	y	y	y
SbTPS II 3.1	V	S	E	D	K	N	L	T	H	H	D	M	N	Q	W	D	S	Q	Y	H	R	y	y	y
SbTPS II 3.2	V		E	D	K	N	L	T	H	H	D	M	N		W	D	S		Y	H	R	y	y	y
SbTPS II 3.3	L	S	E	D	K	N	L	T	H	H	D	M	N	T	W	D	S	T	Y	H	G	y	y	y
SbTPS II 4.1	I	Q	E	D	K	N	L	I	H	Q	D	M	N	F	W	D	S	F	Y	H	R	y	y	n
SbTPS II 4.2	I	A	E	D	K	N	L	V	H	Q	D	M	N	F	W	D	S	F	Y	H	R	y	y	n
SbTPS II 5.1	L	A	I	D	K	N	R	I	H	H	D	L	N	R	W	D	S	R	Y	H	R	y	y	y
SbTPS II 5.3	V	T	I	D	K	N	R	I	H	H	D	L	N	R	W	D	S	R	Y	H	R	y	y	y
SbTPS II 5.4	V	A	F	D	K	N	R	V			D	L	N	R	W	D		R	Y	H	R	n	n	n
SbTPS II 5.5	D		K						F					R			S	R		H	R	n	n	n
**Functional AA Summary**	**F /I /V /L**	**G**	**E**	**R**	**K**	**N**	**L**	**V**	**H**	**Q/H**	**D**	**M/L**	**N**	**R**	**W**	**D**	**I /S /T**	**R**	**Y**	**H**	**R**			

Explanation:

The domain analysis table is color-coded: TPS in Arabidopsis, rice, maie and sorghum are shown in green, blue, yellow and white backgrounds.

Grey backgrounds indicate the amino acids in the TPS domain or the motifs in the HAD domain(a.k.a. TPP domain) are not conserved and probably non-functional.

For the TPS domain, functional amino acids is shown in red, otherwise in black; Any grey grids without a amino acid is due to lacking of the conserved motif.

For the HAD domain, whether the motif is conserved is indicated by letter ("y" = conserved; "n" = not conserved).

TableS10. Details of twenty-nine differentially-expressed candidate genes that were associated with T6P and located in the introgression region of R9188.

geneID	RPKM_RIO_T1	RPKM_RIO_T2	RPKM_RIO_T3	RPKM_RIO_T4	RPKM_BTx406_T1	RPKM_BTx406_T2	RPKM_BTx406_T3	RPKM_BTx406_T4	RPKM_R9188_T1	RPKM_R9188_T2	RPKM_R9188_T3	RPKM_R9188_T4	T6P_cor_RIO	T6P_cor_BTx406	T6P_cor_R9188	Arabidopsis_BLAST hits	Arabidopsis_BLAST hits_Annotation	Rice_BLAST hits	Rice_BLAST hits_Annotation	TF	CW_subtype	CW_type	Hormone1	Hormone2	SugarGene	T6P	Transport	QTL_check	Categories
	Expression levels												Correlation with T6P			Annotation of homologs in Arabidopsis and rice				Annotation in sorghum								Whether the genes located in reported QTLs	
Sobic.001G225800	5	2	2	3	2	2	6	43	4	2	8	9	0.002	-0.792	-0.947	AT1G04500	CCT motif family protein	LOC_Os10g32900.1	CCT motif family protein, expressed	Orphans	na	na	na	na	na	na	na	1	Transcriptional/Post-transcriptional regulation
Sobic.004G186500	6	5	4	6	4	3	4	9	3	0	1	2	0.190	-0.802	-0.613	AT1G47480	alpha/beta-Hydrolases superfamily protein	LOC_Os02g35940.1	gibberellin receptor GID1L2, putative, expressed	na	na	na	na	na	na	na	na	1	metabolism
Sobic.004G191700	9	19	4	10	2	5	6	26	5	5	5	13	-0.683	-0.776	-0.727	AT5G61430	NAC domain containing protein 100	LOC_Os02g36880.3	No apical meristem protein, putative, expressed	NAC	na	na	na	na	na	na	na	1	Transcriptional/Post-transcriptional regulation
Sobic.004G230900	3	2	2	3	4	3	5	12	3	2	4	6	-0.048	-0.869	-0.966	AT1G22330	RNA-binding (RRM/RBD/RNP motifs) family protein	LOC_Os02g51890.1	RNA recognition motif containing protein, putative, expressed	na	na	na	na	na	na	na	na	1	Transcriptional/Post-transcriptional regulation
Sobic.004G265800	17	10	9	13	7	16	18	23	17	12	33	59	0.145	-0.725	-0.897	AT1G26190	Phosphoribulokinase / Uridine kinase family	LOC_Os02g47860.2	uridine kinase, putative, expressed	na	na	na	na	na	na	na	na	1	metabolism
Sobic.004G293000	7	5	3	3	5	2	4	7	5	4	5	8	-0.654	-0.762	-0.840	AT4G00170	Plant VAMP (vesicle-associated membrane protein) family protein	LOC_Os02g44300.1	MSP domain containing protein, expressed	na	na	na	na	na	na	na	na	1	signaling transduction
Sobic.004G300300	51	19	17	41	6	11	10	16	2	0	1	5	0.272	-0.650	-0.839	AT2G46680	homeobox 7	LOC_Os02g43330.1	homeobox associated leucine zipper, putative, expressed	HD-ZIP	na	na	na	na	na	na	na	1	Transcriptional/Post-transcriptional regulation
Sobic.002G271600	8	10	3	5	5	19	56	123	4	10	33	30	-0.824	-0.902	-0.718	AT1G01200	RAB GTPase homolog A3	LOC_Os09g35860.1	ras-related protein, putative, expressed	na	na	na	na	na	na	na	na	1	signaling transduction
Sobic.002G287400	7	7	6	7	3	5	6	7	3	4	6	8	-0.151	-0.681	-0.821	AT1G76350	Plant regulator RWP-RK family protein	LOC_Os09g37710.1	NIN, putative, expressed	RWP-RK	na	na	na	na	na	na	na	1	Transcriptional/Post-transcriptional regulation
Sobic.002G303900	0	0	0	0	0	1	2	9	0	1	2	4	0	-0.823	-0.661	AT5G51460	Haloacid dehalogenase-like hydrolase (HAD) superfamily protein	LOC_Os02g44230	CPuORF22 - conserved peptide uORF-containing transcript, expressed	na	na	na	na	na	na	TPP	na	1	metabolism
Sobic.002G304000	55	84	73	82	41	153	257	408	92	114	243	312	0.128	-0.858	-0.834	AT5G64370	beta-ureidopropionase	LOC_Os07g30170.1	nitrilase, putative, expressed	na	na	na	na	na	na	na	na	1	metabolism
Sobic.002G322000	570	1225	1653	994	73	225	85	43	89	1209	688	227	0.096	0.779	0.707	AT1G61800	glucose-6-phosphate/phosphate translocator 2	LOC_Os07g34006.1	transporter family protein, putative, expressed	na	na	na	na	na	na	na	glucose-6-phosphate/phosphate translocator 2	1	transport
Sobic.002G329600	324	230	141	63	197	60	65	80	132	14	40	25	-0.780	0.285	-0.033	AT4G05180	photosystem II subunit Q-2	LOC_Os07g36080.1	oxygen evolving enhancer protein 3 domain containing protein, expressed	na	na	na	na	na	na	na	na	1	metabolism
Sobic.006G087900	13	8	10	7	7	8	3	4	7	6	1	2	-0.218	0.932	0.765	AT5G47850	CRINKLY4 related 4	LOC_Os04g35890.1	TKL_IRAK_CR4L.3 - The CR4L subfamily has homology with Crinkly4, expressed	na	na	na	na	na	na	na	na	0	signaling transduction
Sobic.006G144600	20	6	7	7	21	12	3	1	15	10	3	2	-0.193	0.817	0.641	AT2G33385	actin-related protein C2B	LOC_Os04g43290.1	ARPC2B, putative, expressed	na	na	na	na	na	na	na	na	0	others: cytoskeleton
Sobic.007G050600	163	215	307	187	61	37	8	5	58	63	22	20	0.109	0.815	0.888	AT3G03050	cellulose synthase-like D3	LOC_Os08g06380.1	CSLF6 - cellulose synthase-like family F; beta1,3;1,4 glucan synthase, expressed	na	1.2.1. CSL_GT2	1.Polysaccharide_synthesis	na	na	na	na	na	0	metabolism
Sobic.007G177000	64	89	77	78	46	98	148	158	69	100	132	150	-0.253	-0.799	-0.655	AT4G35000	ascorbate peroxidase 3	LOC_Os08g43560.1	OsAPx4 - Peroxisomal Ascorbate Peroxidase encoding gene 5,8,9, expressed	na	na	na	na	na	na	na	na	0	metabolism
Sobic.007G178300	28	10	11	9	13	24	10	5	13	18	8	7	-0.273	0.913	0.988	AT4G12350	myb domain protein 42	LOC_Os09g36250.1	MYB family transcription factor, putative, expressed	MYB	na	na	na	na	na	na	na	0	Transcriptional/Post-transcriptional regulation
Sobic.007G181600	20	14	8	8	11	15	32	55	9	8	17	17	-0.663	-0.926	-0.873	AT5G51970	GroES-like zinc-binding alcohol dehydrogenase family protein	LOC_Os08g43190.2	dehydrogenase, putative, expressed	na	na	na	na	na	na	na	na	0	metabolism
Sobic.007G191200	4	1	2	2	15	11	5	2	7	5	3	2	0.033	0.889	0.675	AT5G13170	senescence-associated gene 29	LOC_Os08g42350.1	nodulin MtN3 family protein, putative, expressed	na	na	na	na	na	na	na	SWEET family	0	transport
Sobic.008G111300	3	4	2	4	6	4	6	12	3	2	4	6	0.108	-0.812	-0.960	AT3G18830	polyol/monosaccharide transporter 5	LOC_Os12g32940.1	transporter family protein, putative, expressed	na	na	na	na	na	na	na	polyol transporter (PLT)	0	transport
Sobic.008G157100	12	21	7	9	3	8	12	75	11	8	20	52	-0.843	-0.768	-0.823	AT5G28770	bZIP transcription factor family protein	LOC_Os12g40920.1	bZIP transcription factor domain containing protein, expressed	bZIP	na	na	na	na	na	na	na	0	Transcriptional/Post-transcriptional regulation
Sobic.008G160700	13	16	14	16	11	23	25	33	17	11	27	45	0.122	-0.696	-0.917	AT1G03090	methylcrotonyl-CoA carboxylase alpha chain, mitochondrial / 3-methylcrotonyl-CoA carboxylase 1 (MCCA)	LOC_Os12g41250.1	methylcrotonoyl-CoA carboxylase subunit alpha, mitochondrial precursor, putative, expressed	na	na	na	na	na	na	na	na	0	metabolism
Sobic.009G142200	6	4	3	4	1	6	12	27	0	0	4	5	-0.231	-0.850	-0.873	AT1G01720	NAC (No Apical Meristem) domain transcriptional regulator superfamily protein	LOC_Os05g34830.1	No apical meristem protein, putative, expressed	NAC	na	na	na	na	na	na	na	0	Transcriptional/Post-transcriptional regulation
Sobic.009G165500	20	15	18	16	30	17	10	11	26	17	10	6	0.008	0.631	0.610	AT5G18520	Lung seven transmembrane receptor family protein	LOC_Os05g38720.1	Lung seven transmembrane receptor domain containing protein, putative, expressed	na	na	na	na	na	na	na	na	0	signaling transduction
Sobic.009G219100	33	49	48	46	17	37	46	53	16	22	33	39	0.070	-0.723	-0.746	AT1G68830	STT7 homolog STN7	LOC_Os05g47560.1	serine/threonine-protein kinase SNT7, chloroplast precursor, putative, expressed	na	na	na	na	na	na	na	na	0	signaling transduction
Sobic.009G226300	16	26	24	27	6	20	54	97	13	16	46	72	0.266	-0.917	-0.848	AT4G13400	2-oxoglutarate (2OG) and Fe(II)-dependent oxygenase superfamily protein	LOC_Os05g48230.1	expressed protein	na	na	na	na	na	na	na	na	0	metabolism
Sobic.009G232200	269	161	290	181	304	130	23	7	197	142	22	19	0.158	0.719	0.701	AT2G20520	FASCICLIN-like arabinogalactan 6	LOC_Os05g48900.1	fasciclin domain containing protein, expressed	na	na	na	na	na	na	na	na	0	metabolism
Sobic.010G033000	6	10	10	9	6	4	3	1	8	7	3	3	0.070	0.690	0.696	AT5G23310	Fe superoxide dismutase 3	LOC_Os06g05110.1	superoxide dismutase, chloroplast, putative, expressed	na	na	na	na	na	na	na	na	0	metabolism

na = not applicable

TableS11. Information about QTLs associated with plant height, flowering time, glucose concentration, juice volume, juice weight, non-fibrous carbohydrates and sugar contents, which were identified by previous studies.

Year of reference	QTL name	chromosome	Left coordinate	Right coordinate	TYPE	Traits	Reference
2015	FD1	Chr02	8,031,702	20,388,713	double markers	flowering date	Anne Mocoeur, et al. 2015
2015	FD2	Chr04	61,178,557	62,286,897	double markers	flowering date	Anne Mocoeur, et al. 2015
2015	FD3	Chr08	9,515,032	46,796,389	double markers	flowering date	Anne Mocoeur, et al. 2015
2010	GLUC1	Chr01	68,494,074	69,632,980	double markers	glucose content	Amukelani Lacrecia Shiringani, et al. 2010
2010	GLUC2	Chr03	7,489,898	55,404,530	double markers	glucose content	Amukelani Lacrecia Shiringani, et al. 2010
2011	JW1	Chr01	16,624,307	50,203,705	double markers	juice weight	Yan-an Guan, et al. 2011
2011	JW2	Chr01	50,068,342	50,645,315	double markers	juice weight	Yan-an Guan, et al. 2011
2011	JW4	Chr07	57,780,614	59,111,746	double markers	juice weight	Yan-an Guan, et al. 2011
2011	JW5	Chr09	57,312,365	58,975,869	double markers	juice weight	Yan-an Guan, et al. 2011
2010	PHT1	Chr01	23,702,809	61,146,468	double markers	plant height	Amukelani Lacrecia Shiringani, et al. 2010
2010	PHT10	Chr08	46,039,745	48,122,969	double markers	plant height	Amukelani Lacrecia Shiringani, et al. 2010
2015	PHT11	Chr09	16,146	2,589,508	double markers	plant height	Anne Mocoeur, et al. 2015
2015	PHT2	Chr01	62,704,567	66,681,750	double markers	plant height	Anne Mocoeur, et al. 2015
2010	PHT3	Chr10	11,029,735	11,037,083	single marker	plant height	Amukelani Lacrecia Shiringani, et al. 2010
2015	PHT4	Chr10	3,630,327	4,593,432	double markers	plant height	Anne Mocoeur, et al. 2015
2010	PHT5	Chr04	48,757,775	51,234,749	double markers	plant height	Amukelani Lacrecia Shiringani, et al. 2010
2010	PHT6	Chr04	58,537,697	61,178,168	double markers	plant height	Amukelani Lacrecia Shiringani, et al. 2010
2015	PHT7	Chr04	57,538,106	58,537,974	double markers	plant height	Anne Mocoeur, et al. 2015
2010	PHT8	Chr06	58,217,493	58,217,656	single marker	plant height	Amukelani Lacrecia Shiringani, et al. 2010
2015	PHT9	Chr07	50,339,917	56,155,869	double markers	plant height	Anne Mocoeur, et al. 2015
2008	SUG1	Chr01	50,203,535	55,065,768	double markers	sugar	Kimberley B. Ritter, et al. 2008
2011	SUG10	Chr03	7,489,898	52,308,712	double markers	sugar	Yan-an Guan, et al. 2011
2002	SUG11	Chr04	51,234,586	67,894,310	double markers	sugar	A. Natoli, et al. 2002
2010	SUG12	Chr04	58,537,697	58,537,974	single marker	sugar	Amukelani Lacrecia Shiringani, et al. 2010
2015	SUG13	Chr04	51,875,816	55,385,999	double markers	sugar	Anne Mocoeur, et al. 2015
2002	SUG14	Chr05	5,686,170	11,099,019	double markers	sugar	A. Natoli, et al. 2002
2008	SUG15	Chr05	1,907,420	11,099,019	double markers	sugar	Kimberley B. Ritter, et al. 2008
2010	SUG16	Chr06	58,217,493	58,217,656	single marker	sugar	Amukelani Lacrecia Shiringani, et al. 2010
2015	SUG17	Chr06	1,822,330	1,908,756	double markers	sugar	Anne Mocoeur, et al. 2015
2011	SUG18	Chr07	57,780,614	59,111,746	double markers	sugar	Yan-an Guan, et al. 2011
2008	SUG19	Chr08	45,992,122	50,413,945	double markers	sugar	Kimberley B. Ritter, et al. 2008
2010	SUG2	Chr01	50,608,251	50,608,477	single marker	sugar	Amukelani Lacrecia Shiringani, et al. 2010
2010	SUG20	Chr08	48,122,810	50,414,003	double markers	sugar	Amukelani Lacrecia Shiringani, et al. 2010
2010	SUG21	Chr08	46,039,745	48,122,969	double markers	sugar	Amukelani Lacrecia Shiringani, et al. 2010
2015	SUG22	Chr08	1,553,582	1,971,577	double markers	sugar	Anne Mocoeur, et al. 2015
2011	SUG3	Chr01	50,068,342	50,645,315	double markers	sugar	Yan-an Guan, et al. 2011
2011	SUG4	Chr01	55,721,520	58,712,796	double markers	sugar	Yan-an Guan, et al. 2011
2010	SUG5	Chr10	11,036,814	47,161,323	double markers	sugar	Amukelani Lacrecia Shiringani, et al. 2010
2002	SUG6	Chr02	5,700,033	61,503,893	double markers	sugar	A. Natoli, et al. 2002
2010	SUG7	Chr02	61,887,961	61,888,140	single marker	sugar	Amukelani Lacrecia Shiringani, et al. 2010
2010	SUG8	Chr02	5,076,675	13,161,221	double markers	sugar	Amukelani Lacrecia Shiringani, et al. 2010
2011	SUG9	Chr02	40,504,003	60,421,867	double markers	sugar	Yan-an Guan, et al. 2011
2016	NFC1_2016_chr04	Chr04	63,201,409	63,401,429	GWAS	n-fibrous carbohydrates (NF	Brenton et al. 2016
2016	NFC2_2016_chr04	Chr04	63,247,613	63,447,623	GWAS	n-fibrous carbohydrates (NF	Brenton et al. 2016
2015	JV_2015_chr06	Chr06	51,666,559	51,901,476	GWAS	Juice volume	Burks et al. 2015
2016	NFC3_2016_chr06	Chr06	4,220,818	4,430,906	GWAS	n-fibrous carbohydrates (NF	Brenton et al. 2016
2016	NFC4_2016_chr06	Chr06	49,673,083	49,884,457	GWAS	n-fibrous carbohydrates (NF	Brenton et al. 2016
